# Supplementary material for: High-throughput thermodynamic fingerprinting of protein–ligand interactions by DNA-directed focal molography
Source: PLoS One. 2026 Oct 22;21(10):e0354008. doi: 10.1371/journal.pone.0354008 (PMC13690234; doi:10.1371/journal.pone.0354008)
Supplement: Supplementary file 1 [file pone.0354008.s001.pdf]

Supporting information

S1. Chemical structures of cAMP analogs

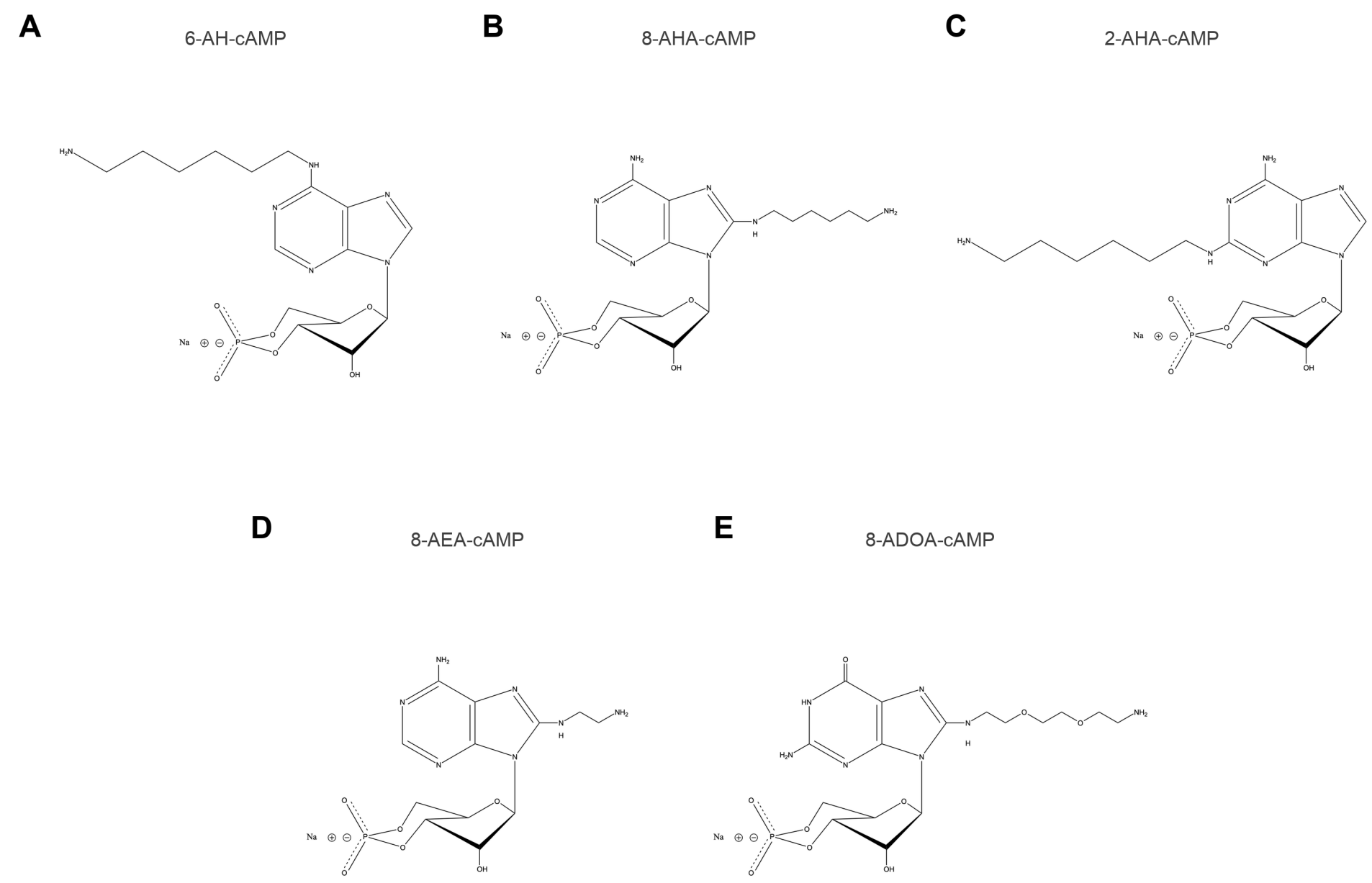

S1 Fig. Chemical structures of the five cAMP analogs used in this study. (A) 6-AH-cAMP, (B) 8-AHA-cAMP, (C) 2-AHA-cAMP, (D) 8-AEA-cAMP, and (E) 8-ADOA-cAMP. All analogs carry a primary amine group enabling covalent conjugation to activated oligonucleotides via NHS-ester chemistry. The modification position (N^6, C2, or C8 of the adenine ring) and linker length and chemistry differ among the analogs.

S2. MACS® Matchmaker focal molography setup

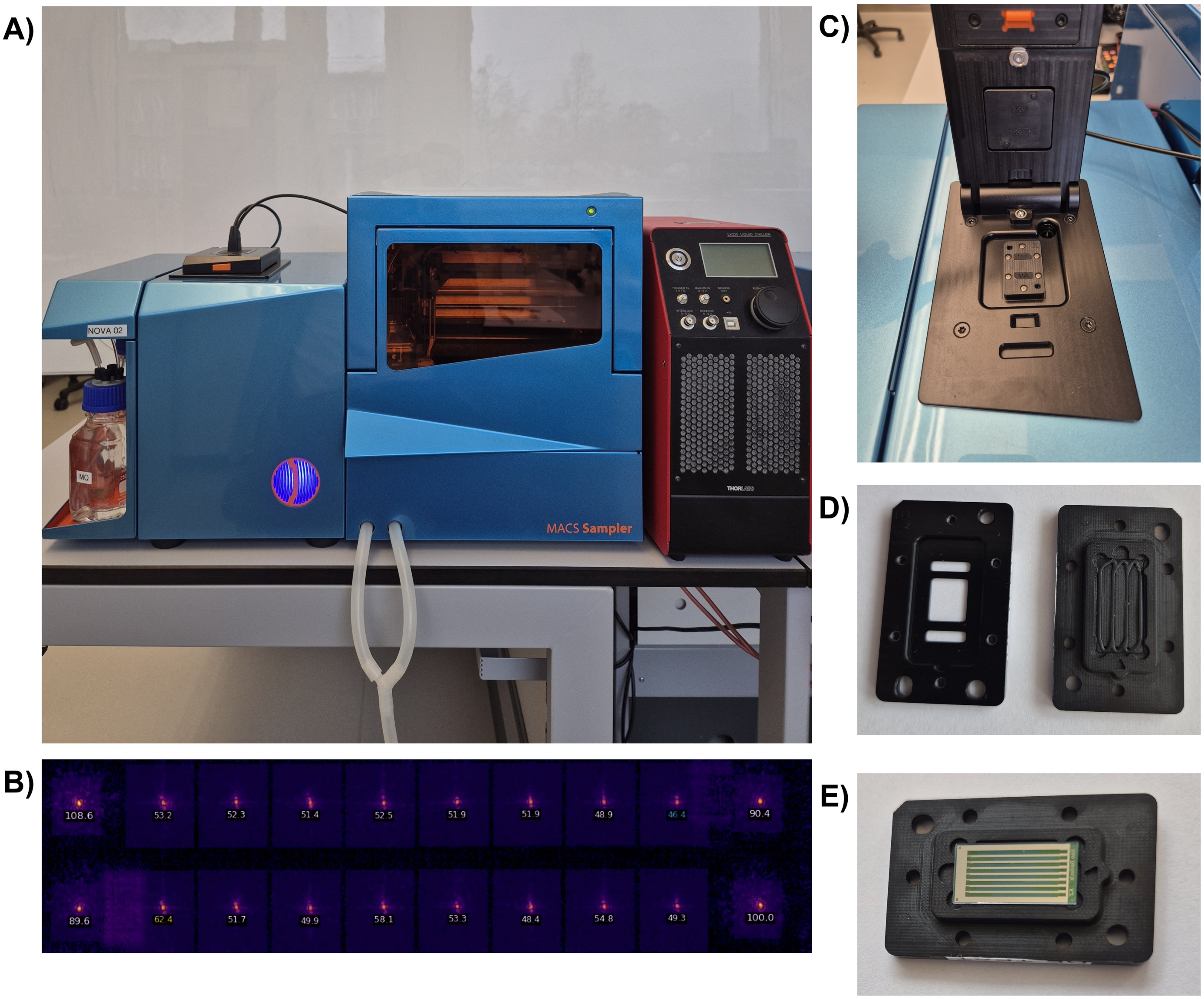

S2 Fig. MACS® Matchmaker focal molography setup. **(A)** From left to right: the MACS Matchmaker main instrument with integrated buffer and pump unit; the MACS Sampler for automated sample injection, accommodating up to two 384-well plates (here configured with two 48-position aluminum grids for glass vials); and the Thorlabs LK220 thermoelectric liquid heater/chiller for precise temperature control of the sample stage. **(B)** Screw-type chip assembly mounted on top of the reader prior to closing the fluidic head. **(C)** Screw-type chip assembly with three-channel flow chamber. The baseplate is made from black anodized aluminum, the fluidic body from polyetheretherketone (PEEK), and the seal from injection-molded silicone. **(D)** Molographic sensor chip placed onto the flow cell assembly. The chip features an 8 × 8 grid with a total of 64 individual molographic measurement spots. **(E)** Typical molographic image processed by the instrument software to extract 16 sensorgrams; the flow cell covers two rows of 8 mollograms each.

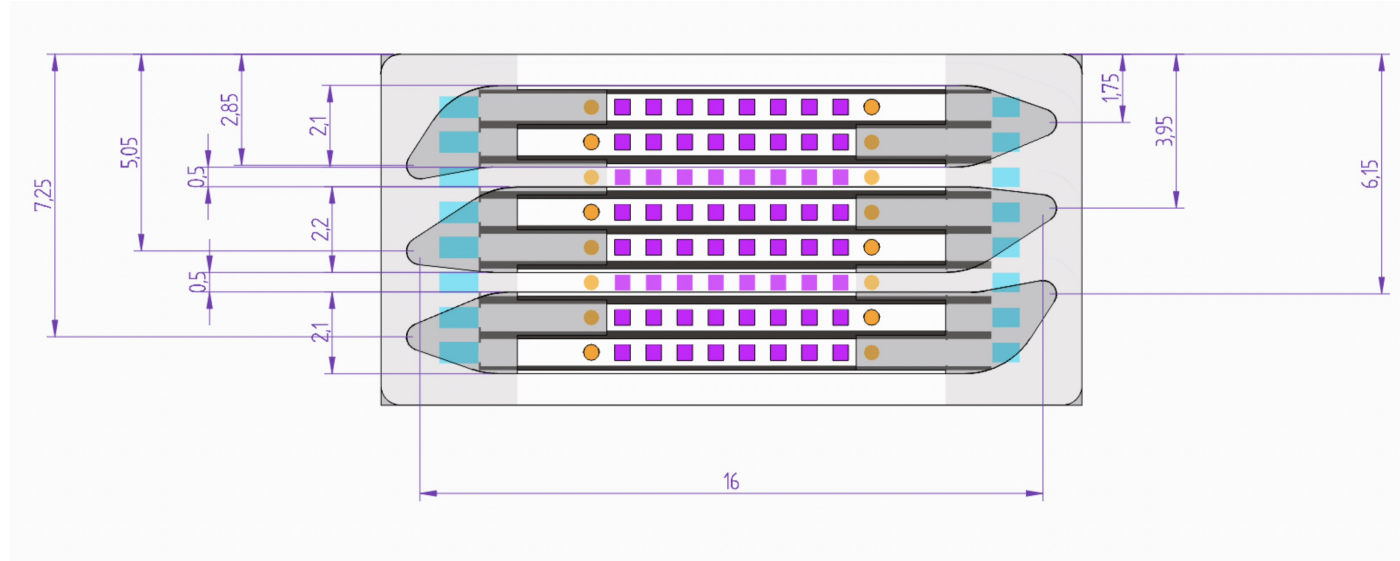

S2 Fig (b). Mechanical dimensions of the MACS® Matchmaker three-channel flow chamber. Each flow channel has a width of 2.1 mm, a height of 100 µm, and a total length of 16 mm from inlet to outlet, spanning two rows of molograms.

S3. Temperature ramp validation

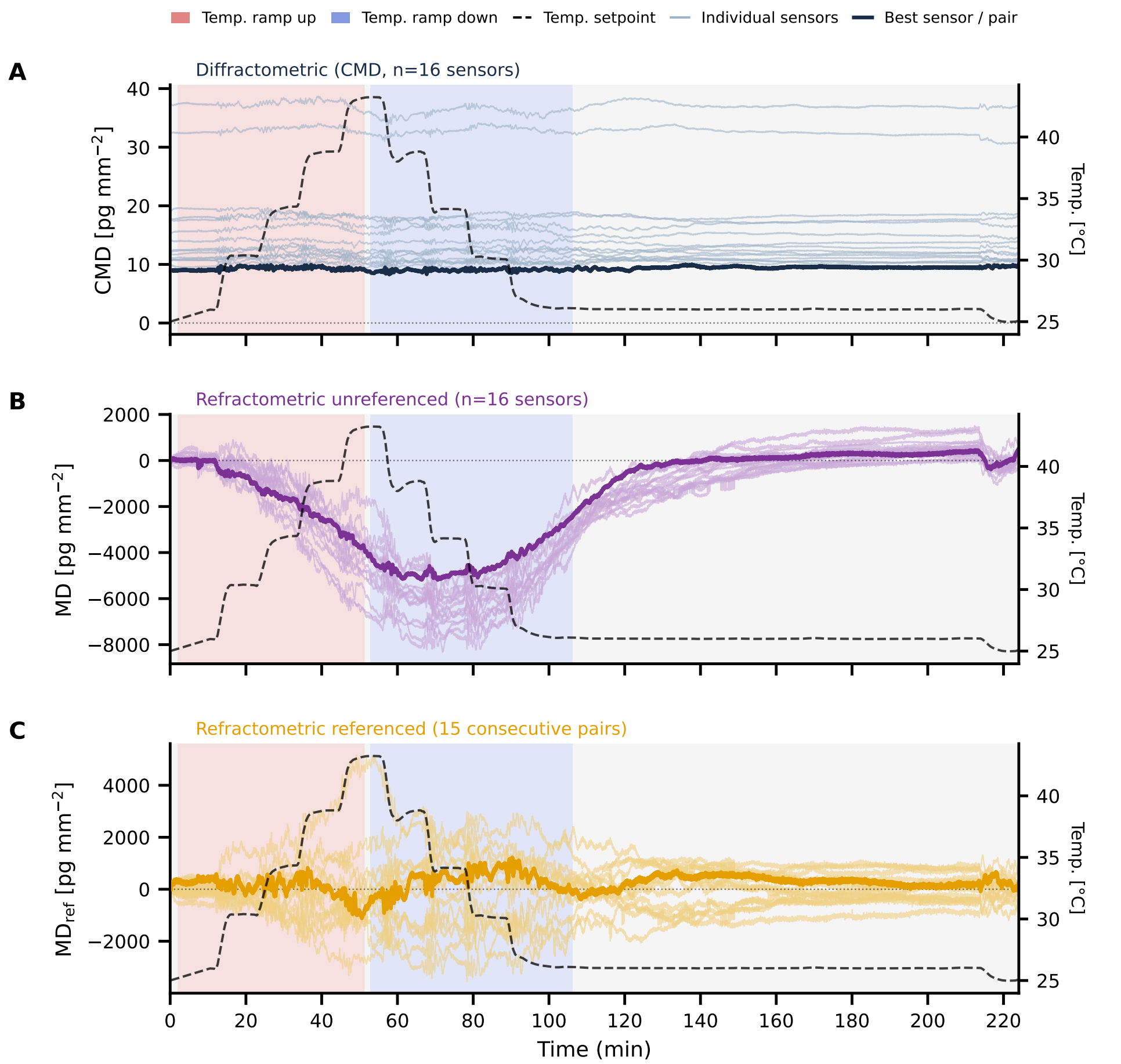

S3 Fig. Diffractometric focal molography is insensitive to temperature; refractometric detection shows large bulk artifacts (MACS® Matchmaker). Buffer-only measurements during a continuous 25–45–25 °C temperature ramp (∼100 min ramp, full 224 min recording shown). **(A)** CMD (coherent mass density, diffractometric): all 16 sensor traces (light) and the best sensor by minimum variance (bold). Signal remains within ±1 pg mm^{-2} throughout the ramp. **(B)** MD (mass density, refractometric, unreferenced): all 16 sensor traces and best sensor. The bulk refractive index shift with temperature produces a large apparent signal change of several thousand pg mm^{-2}. **(C)** MD$_{ref}$ (consecutive sensor-pair subtraction): all 15 pairs (light) and best pair (bold). Active referencing reduces the artifact substantially, but the best pair still spans up to ∼±1200 pg mm^{-2}. Red shading: ramp-up phase; blue shading: ramp-down phase. Dashed black line: temperature setpoint (right axis, all panels).

S4. Specificity and control experiments

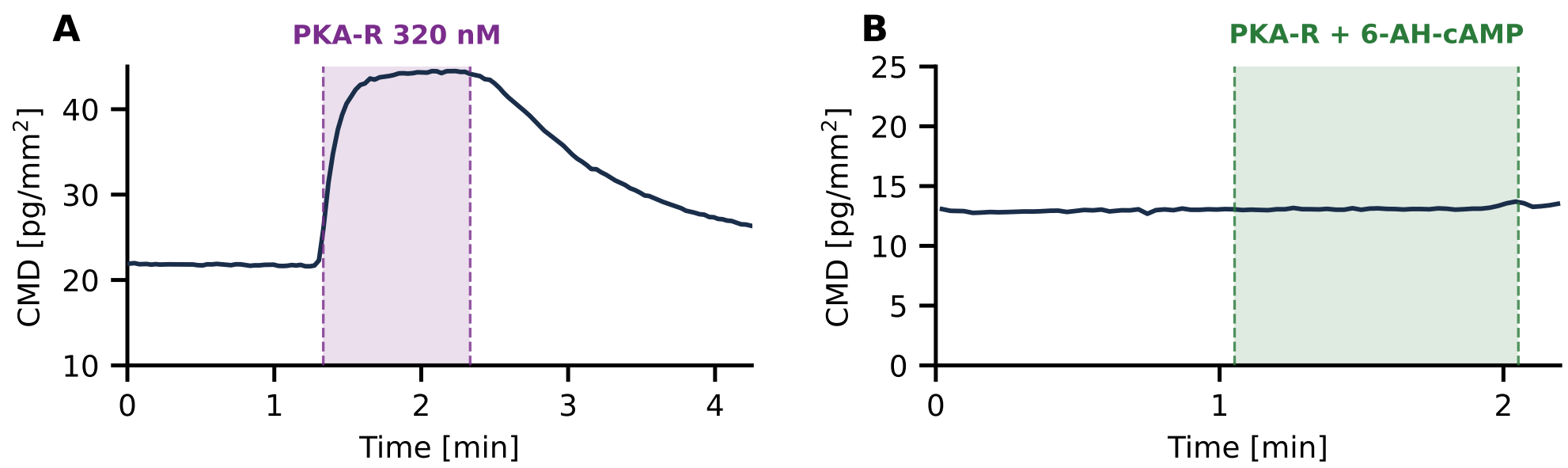

S4 Fig (a). Positive and negative control experiments validating specific PKA-R binding to immobilized cAMP derivatives. (A) Positive control. PKA-R was injected at 320 nM in *buffer A* over the sensor chip surface. This concentration was selected based on a previous study of the same interaction to ensure a detectable signal [1]. The injection resulted in a sharp and substantial increase in coherent mass density (≈20 pg/mm^2 within 30 seconds), consistent with successful and specific binding of free PKA-R to immobilized cAMP derivatives. **(B)** Negative control. A pre-formed PKA-R–cAMP complex (160 nM PKA-R with 1.6 µM 6-AH-cAMP, 1:10 molar ratio) was injected to occupy nearly all PKA-R binding sites before exposure to the chip. The injection produced no significant change in coherent mass density (fluctuations ≈0.5 pg/mm^2, consistent with baseline noise), supporting that the observed binding response arises from free, unbound PKA-R molecules.

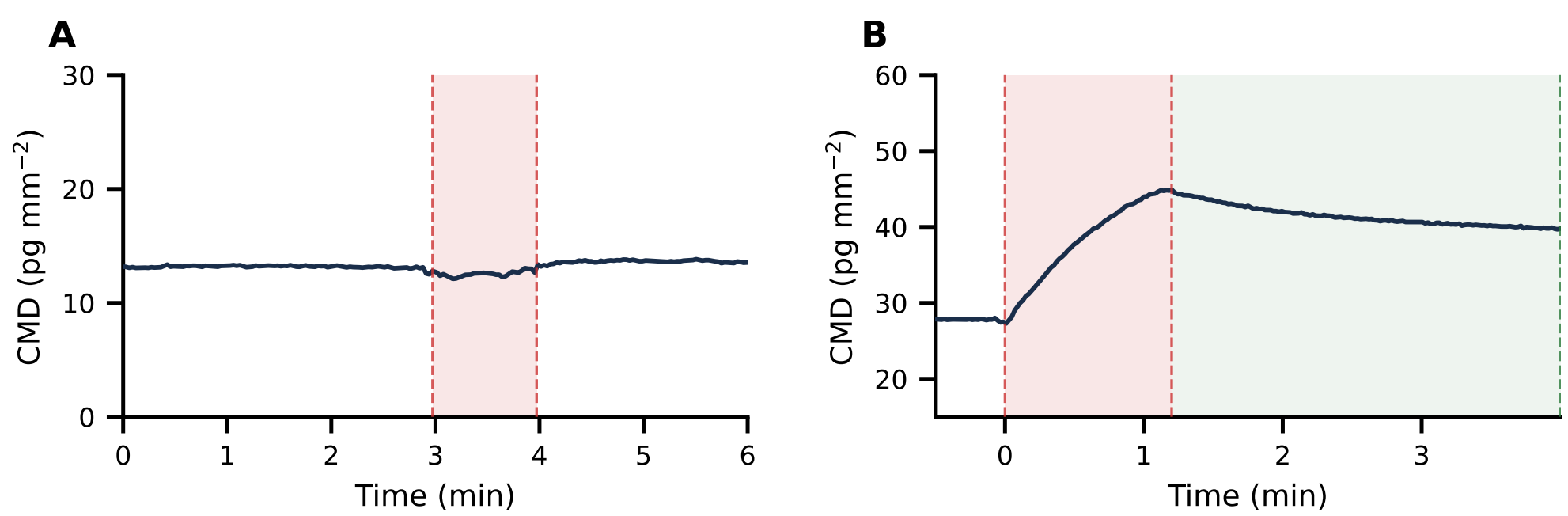

S4 Fig (b). Non-specific binding controls for two human serum preparations. (A) Blank injection of 50% TSH-depleted human serum (Serum 1, lot HD2403001) on the Callisto Pre-series reader over a 6-AH-cAMP-functionalized sensor. No detectable change in coherent mass density was observed (<1 pg mm^{-2}), indicating no detectable non-specific adsorption for this preparation under these conditions. Median of $n = 9$ sensor traces is shown. **(B)** Blank injection of 50% normal human serum (Serum 2, lot U1123331304) at 20°C over a 6-AH-cAMP chip on the MACS® Matchmaker. The coherent mass density reaches approximately 16 pg mm^{-2} and partially recovers upon buffer washout, indicating measurable non-specific adsorption from this preparation. Median of $n = 16$ sensor traces is shown. The contrasting behavior of the two preparations demonstrates that non-specific binding is serum-preparation-specific and must be characterized independently for each experimental context. In both panels, red shading marks the serum injection window; the green shading in (B) additionally marks the subsequent buffer washout phase.

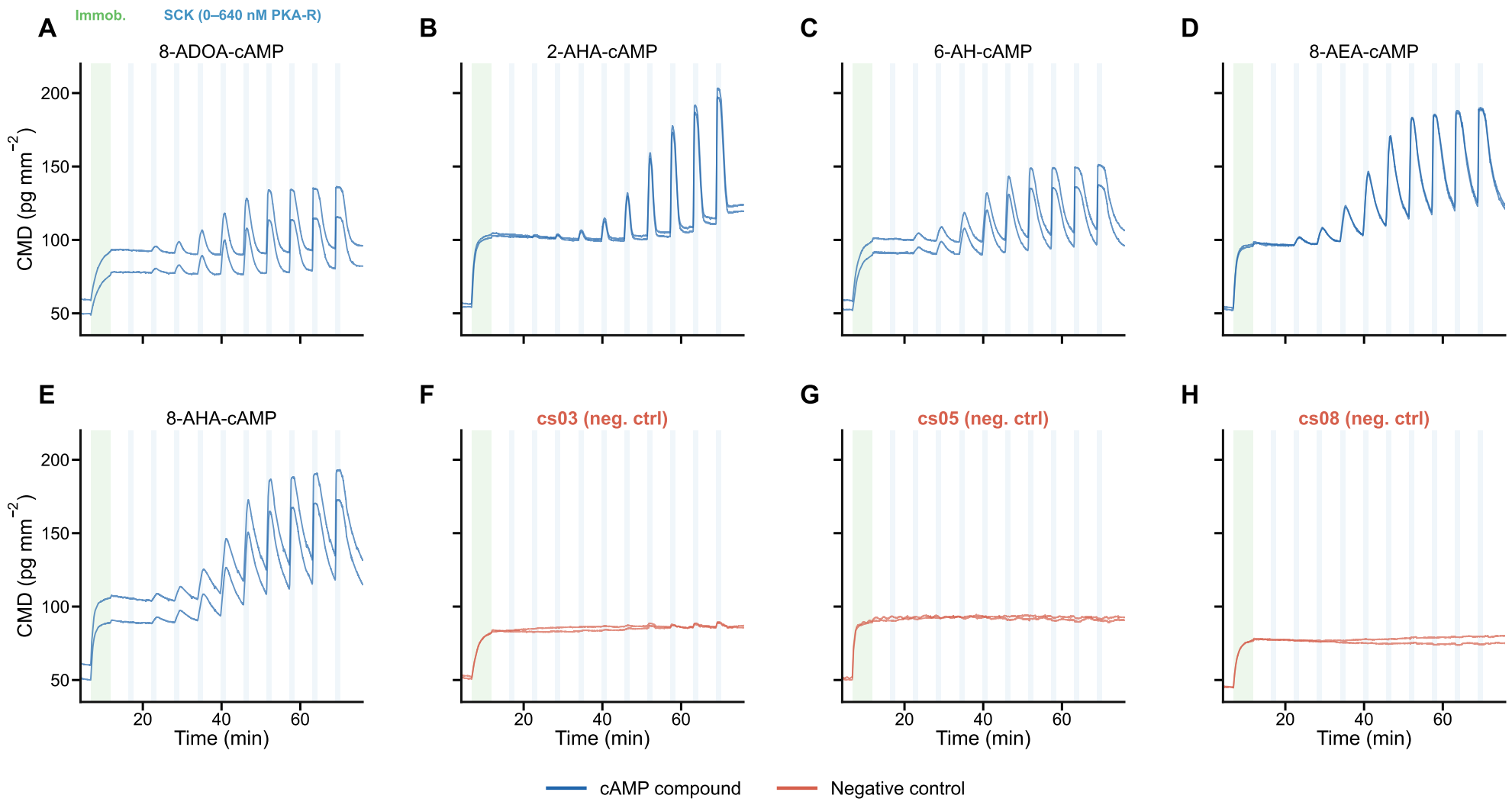

S4 Fig (c). DDI-SCK specificity: active compounds vs. negative control oligonucleotides at 25 °C. Full sensorgrams (immobilization and first SCK cycle) for all eight oligo columns recorded simultaneously on the same chip. **(A–E)** Five cAMP–oligonucleotide conjugates (blue): 8-ADOA-cAMP (A), 2-AHA-cAMP (B), 6-AH-cAMP (C), 8-AEA-cAMP (D), 8-AHA-cAMP (E), all showing concentration-dependent binding to PKA-R (0–640 nM, ascending staircase). **(F–H)** Three negative control oligonucleotides (red): cs03 (F), cs05 (G), cs08 (H), showing no specific PKA-R binding during the SCK phase, supporting that the measured kinetic signal originates from cAMP–PKA-R interaction at the cognate mologram spots. Green shading marks the immobilization injection (200 nM cAMP–oligo mix, 5 min, 10 µL/min); blue shading marks individual PKA-R association injections (72 s each, 150 µL/min). Each panel shows two sensor replicates (rows R4 and R5).

S5. Kinetic measurements in buffer and serum at 20 °C

All 16 sensors (rows R4–R5, columns C1–C8) were fitted individually at 20 °C for each of the four conditions: 6-AH-cAMP in buffer A, 6-AH-cAMP in 50% human serum, 8-AHA-cAMP in buffer A, and 8-AHA-cAMP in 50% human serum. Each panel shows the measured signal, color-coded by PKA-R concentration, with the corresponding 1:1 Langmuir fit (black dashed line). Gray shading indicates the association phase; dotted vertical lines mark the association start and end. For buffer A experiments, the highest concentration (640 nM) was excluded from fitting.

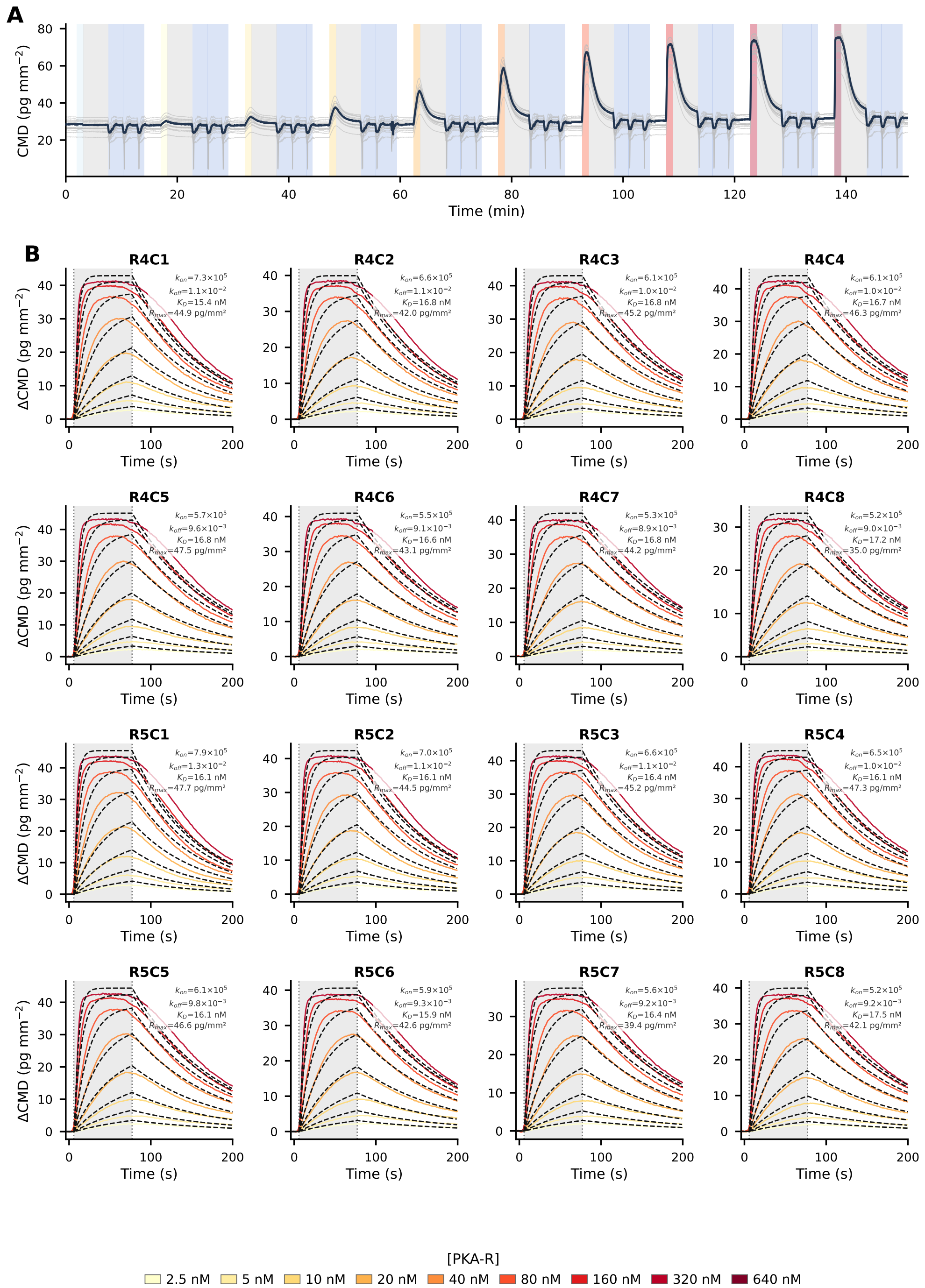

S5 Fig (a). All 16 sensors: 6-AH-cAMP in buffer A, 20 °C. (**A**) Full raw MCK sensorgram showing all 16 sensor traces (light gray) with the median sensor highlighted (dark navy). (**B**) Per-sensor 1:1 Langmuir kinetic fits; ΔCMD signal color-coded by [PKA-R] (2.5–640 nM) with fits (black dashed). 640 nM excluded from fitting.

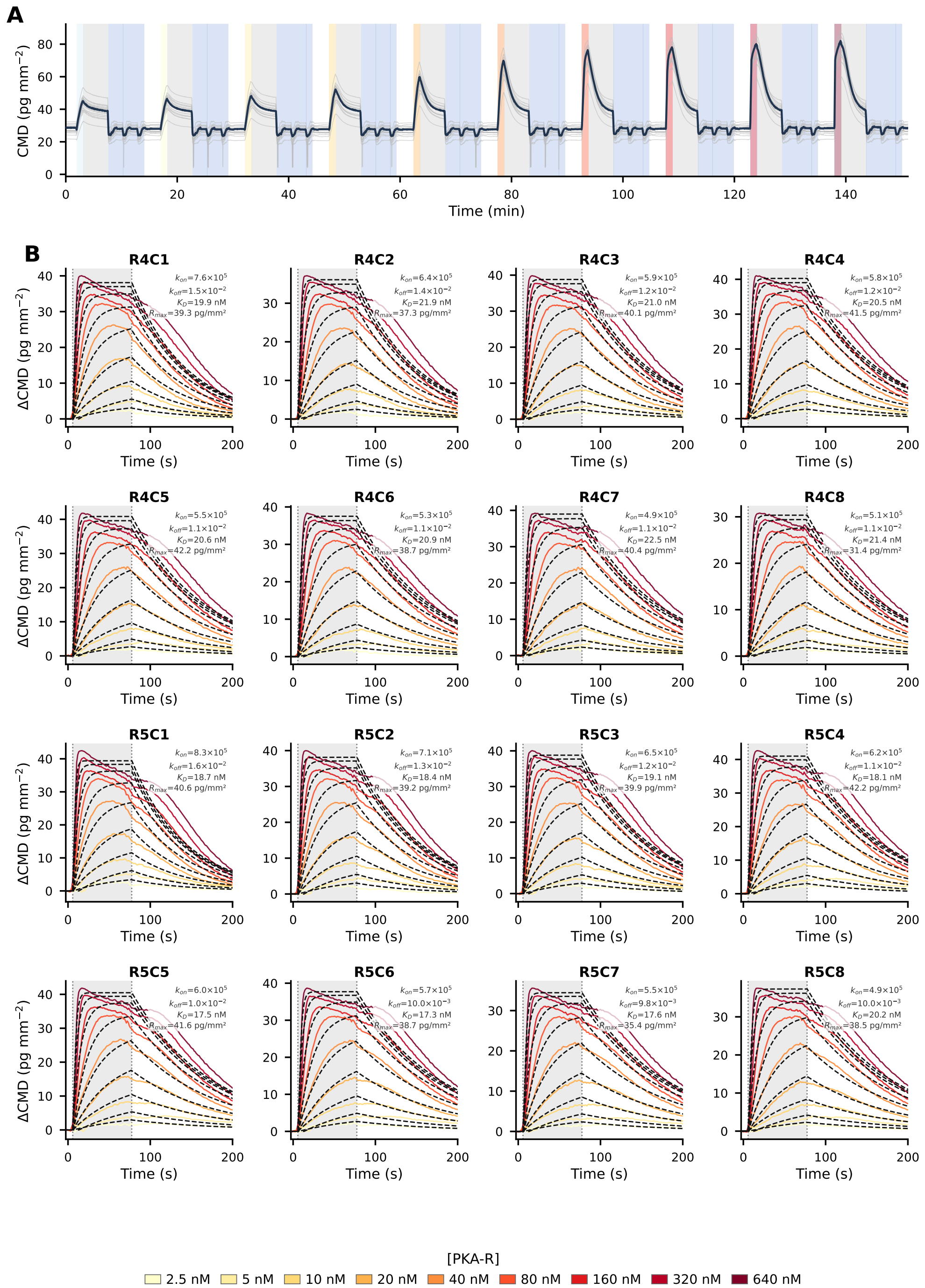

S5 Fig (b). All 16 sensors: 6-AH-cAMP in 50% human serum, 20 °C.
(**A**) Full raw MCK sensorgram showing all 16 sensor traces (light gray) with the median sensor highlighted (dark navy). (**B**) Per-sensor 1:1 Langmuir kinetic fits; ΔCMD signal color-coded by [PKA-R] (2.5–640 nM) with fits (black dashed).

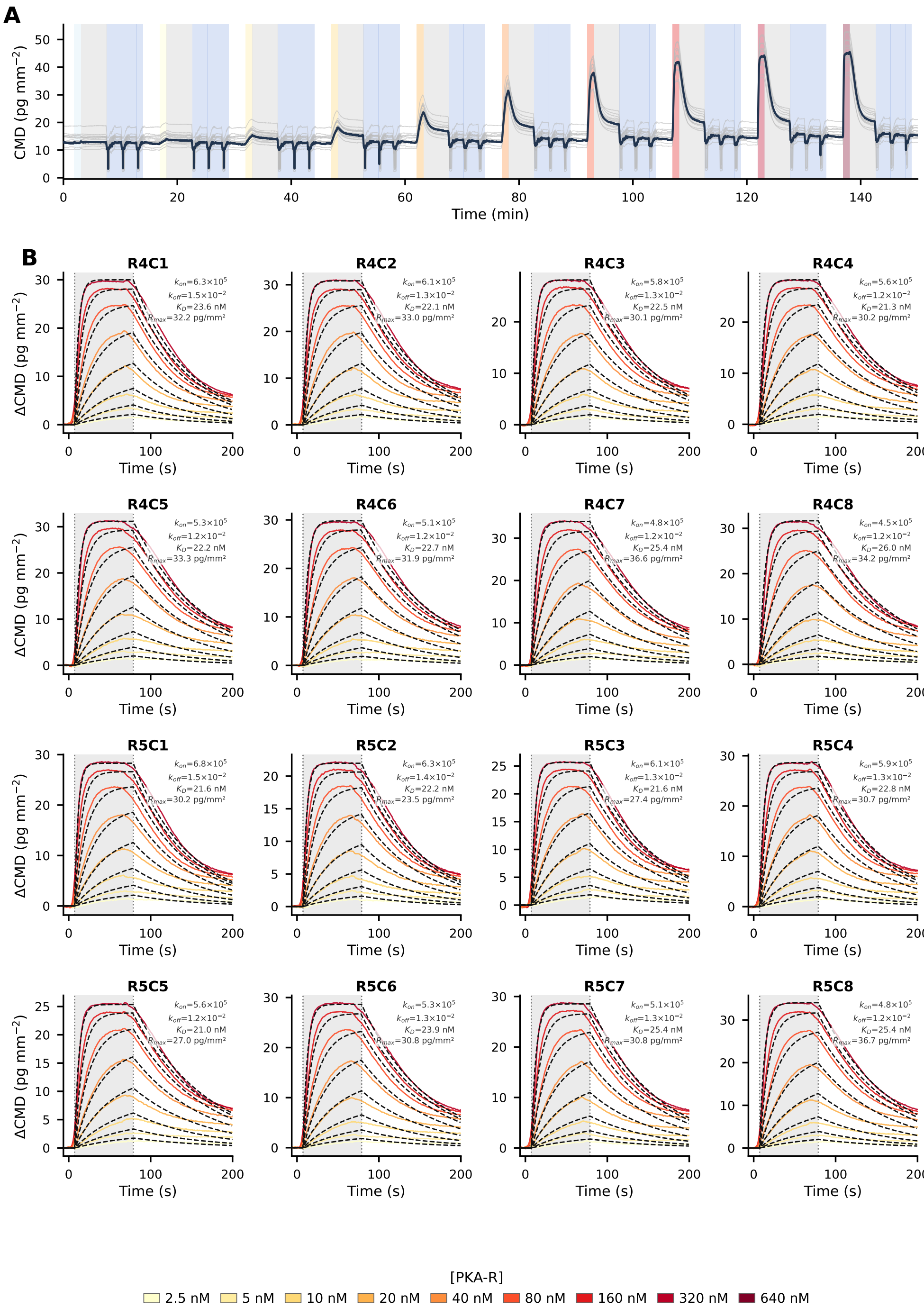

S5 Fig (c). All 16 sensors: 8-AHA-cAMP in buffer A, 20 °C. (**A**) Full raw MCK sensorgram showing all 16 sensor traces (light gray) with the median sensor highlighted (dark navy). (**B**) Per-sensor 1:1 Langmuir kinetic fits; ΔCMD signal color-coded by [PKA-R] (2.5–640 nM) with fits (black dashed). 640 nM excluded from fitting.

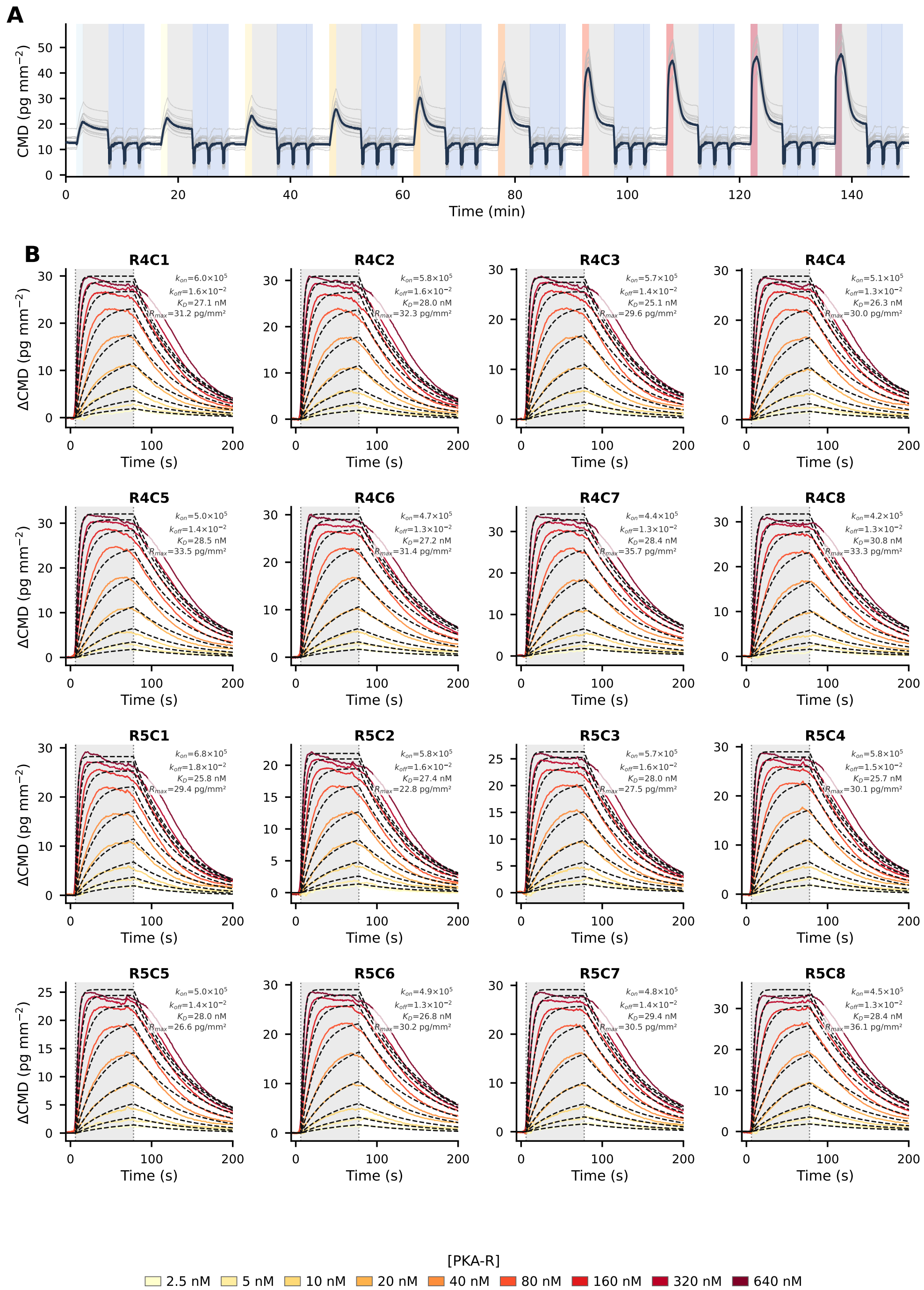

S5 Fig (d). All 16 sensors: 8-AHA-cAMP in 50% human serum, 20 °C. (**A**) Full raw MCK sensorgram showing all 16 sensor traces (light gray) with the median sensor highlighted (dark navy). (**B**) Per-sensor 1:1 Langmuir kinetic fits; ΔCMD signal color-coded by [PKA-R] (2.5–640 nM) with fits (black dashed).

S6. Callisto Pre-series prototype instrument

Preliminary experiments and initial kinetic characterization were performed on a prototype instrument from the “Callisto Pre-series Generation” developed by lino Biotech AG (Adliswil, Zurich, Switzerland). These experiments are included here to document the developmental progression of the focal molography platform and to show that compatibility with serum-like matrices was already evident at this earlier stage. In particular, measurements with a thyroid-stimulating hormone (TSH)-depleted human serum preparation showed no detectable non-specific CMD response, supporting the conclusion that diffractometric focal molography can resolve specific binding in complex biological samples with low matrix interference. Because the later MACS® Matchmaker platform provided higher sensor count, improved statistics, and the fully developed dataset used for temperature-dependent thermodynamic profiling, the Callisto data are presented here as supporting developmental and kinetic evidence rather than as the primary quantitative basis of the study.

The custom-built device is equipped with three data generation channels: a molographic detection channel (785 nm laser) measuring focal spot intensity, a refractometric detection channel measuring the shift of the spot in the focal plane, and a fluorescence microscopy detection channel (660 nm excitation) in TIRF mode [2, 3]. Both lasers are coupled into a dielectric slab waveguide made from Ta_2O_5 [3, 4]. The instrument uses quadruple flow chambers (PEEK, Kalrez seals) covering one row of 9 molograms each (1 mm wide, 100 µm high, 12 mm long). The sensor chip measures 9 mm × 18 mm with 400 µm × 400 µm spots in a 6 × 9 grid (54 spots total). Automated sample delivery used an Alias Autosampler (Spark Holland) with a Hamilton PSD/4 pump; temperature control used a Thorlabs LK220 liquid chiller (±0.1 K). Data were collected at approximately 0.3 Hz using proprietary lino Biotech AG software. A detailed image of the complete experimental setup is shown in S6 Fig (a).

For serum experiments on the Callisto Pre-series, a TSH-depleted processed human serum was used (pediatric donor pool; manufactured 2024-03-20; tested and found negative for anti-HIV 1+2, anti-HCV, HBsAg, HIV 1 and HCV RNA, and syphilis serology; Seqens, lot HD2403001), diluted to 50% in *buffer A*. Blank injections of this preparation produced no detectable non-specific adsorption on the sensor surface (S4 Fig (b)A), in contrast to the normal human serum used on the MACS® Matchmaker (see main text).

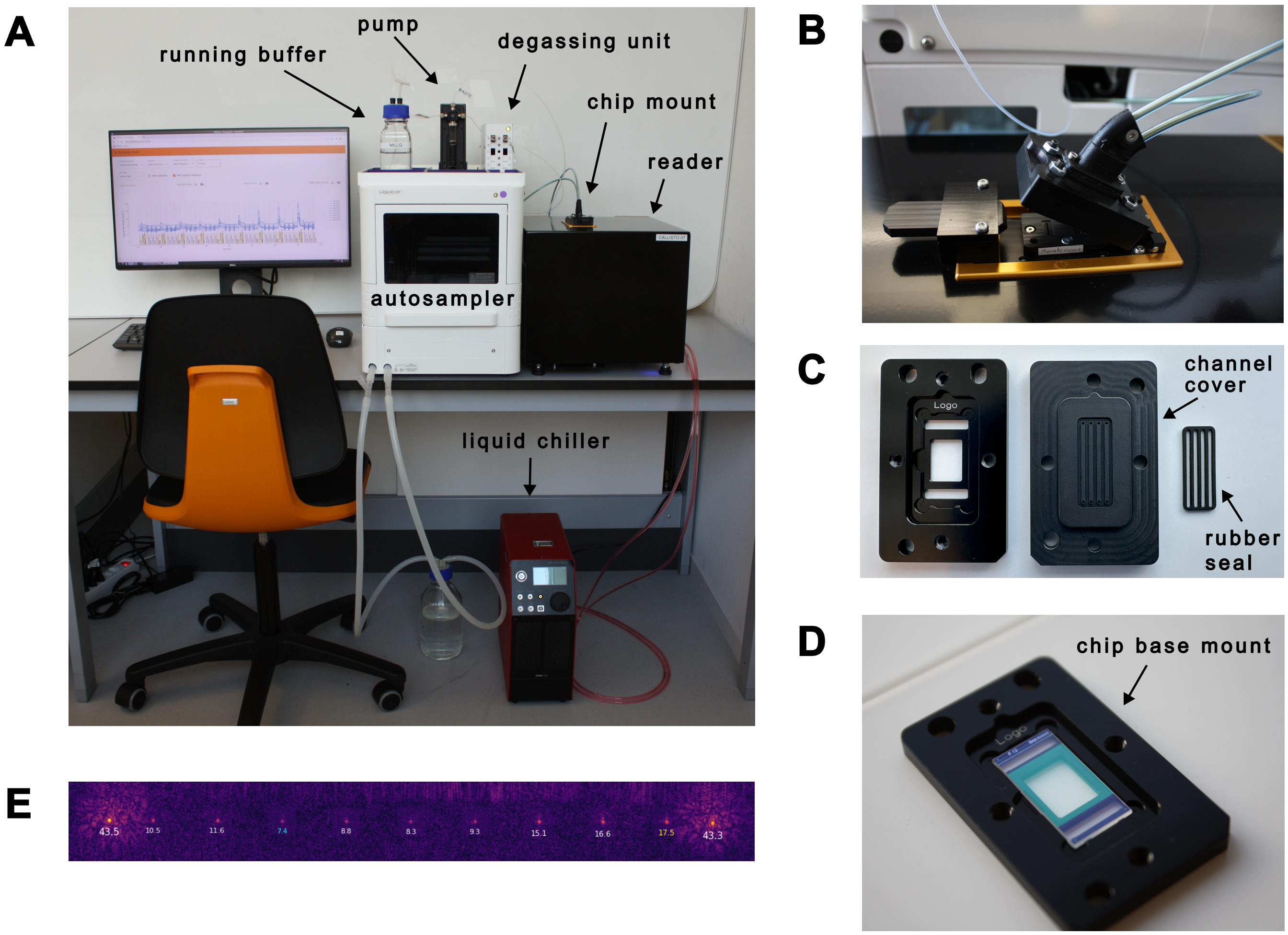

S6 Fig (a). Instrumentation setup for focal molography experiments (Callisto Pre-series prototype). **(A)** Overview of the complete experimental setup, including the reader instrument, autosampler, pump, degassing unit, chip mount, and liquid chiller for precise temperature control. **(B)** Close-up of the chip mount with connected fluidic lines. **(C)** Disassembled view of the flow chamber assembly, showing the aluminum chip base mount (left), PEEK flow chamber with integrated channels (middle), and laser-cut Kalrez rubber seal (right). **(D)** Aluminum chip base mount with the sensor chip mounted. **(E)** Representative molographic readout image from the instrument showing diffracted signal spots along a measurement row (9 spots). The two mologram on the left and right are reference molograms.

S1 Table. Injection sequence for multi-cycle kinetics (MCK) experiments on the Callisto Pre-series prototype. Note the lower flow rates compared to the MACS® Matchmaker (Table 2), reflecting the smaller flow chamber dimensions of the prototype instrument.

Type	Sample	Duration	Flow rate
Baseline	Buffer A	1 min	30 µL/min
Association	PKA-R	1 min	30 µL/min
Dissociation	Buffer A	3 min	200 µL/min
Regeneration	3 M GuHCl / 125 mM NaOH	40 s	300 µL/min
Rinse	Buffer A	1 min	100 µL/min

Kinetic characterization in buffer and human serum on the Callisto Pre-series

To establish an early kinetic benchmark for the PKA-R–cAMP interaction and to assess whether focal molography could resolve binding in serum-like matrices, multi-cycle kinetic (MCK) experiments were conducted on the Callisto Pre-series instrument in both *buffer A* and 50% human serum at 20°C with intermediate regeneration; the full injection sequence is given in S1 Table. PKA-R concentrations ranged from 2.5 nM to 640 nM and intermediate regeneration cycles with 3 M guanidine hydrochloride (GuHCl) and 125 mM NaOH successfully restored the baseline after each cycle. A representative titration series in *buffer A* for the 6-AH-cAMP derivative is shown in S6 Fig (b)A, demonstrating binding responses across all concentrations. The corresponding titration series in 50% human serum produced sensorgrams comparable in shape and absolute signal to those obtained in buffer, providing early evidence that the sensor can detect PKA-R–cAMP interactions in complex media (S6 Fig (b)B). Although minor signal fluctuations were observed in the serum measurements, they were attributed to variable recognition and tracking of the focal spots by the prototype image-processing software rather than to non-specific binding from serum components.

It is important to note that the sensorgrams in buffer and serum do not start at the same coherent mass density or saturate at the same level. This discrepancy arises because each mologram exhibits a slightly different quality (analyte efficiency) due to the reactive immersion lithography process [5]. This does not influence the qualitative comparison of kinetic behavior, as the relative shape of the sensorgrams after proper normalization is independent of mologram quality.

S6 Fig (c) presents a comprehensive panel of kinetic fits for both cAMP derivatives measured under two conditions: *buffer A* and 50% human serum. Each condition is represented by three replicate measurements (labeled i, ii, iii), resulting in 12 kinetic sensorgrams. Overall, the binding responses are consistent in shape in buffer and serum, reinforcing the conclusion that diffractometric focal molography can resolve specific binding in a serum matrix already on the prototype platform. SI Tables S2–S3 summarize the triplicate kinetic measurements, which remain in the nanomolar affinity range under all conditions. These Callisto results provide developmental support for serum compatibility and early kinetic reproducibility, whereas the higher-statistics MACS® Matchmaker measurements reported in the main text form the primary quantitative basis for the final kinetic and thermodynamic analysis.

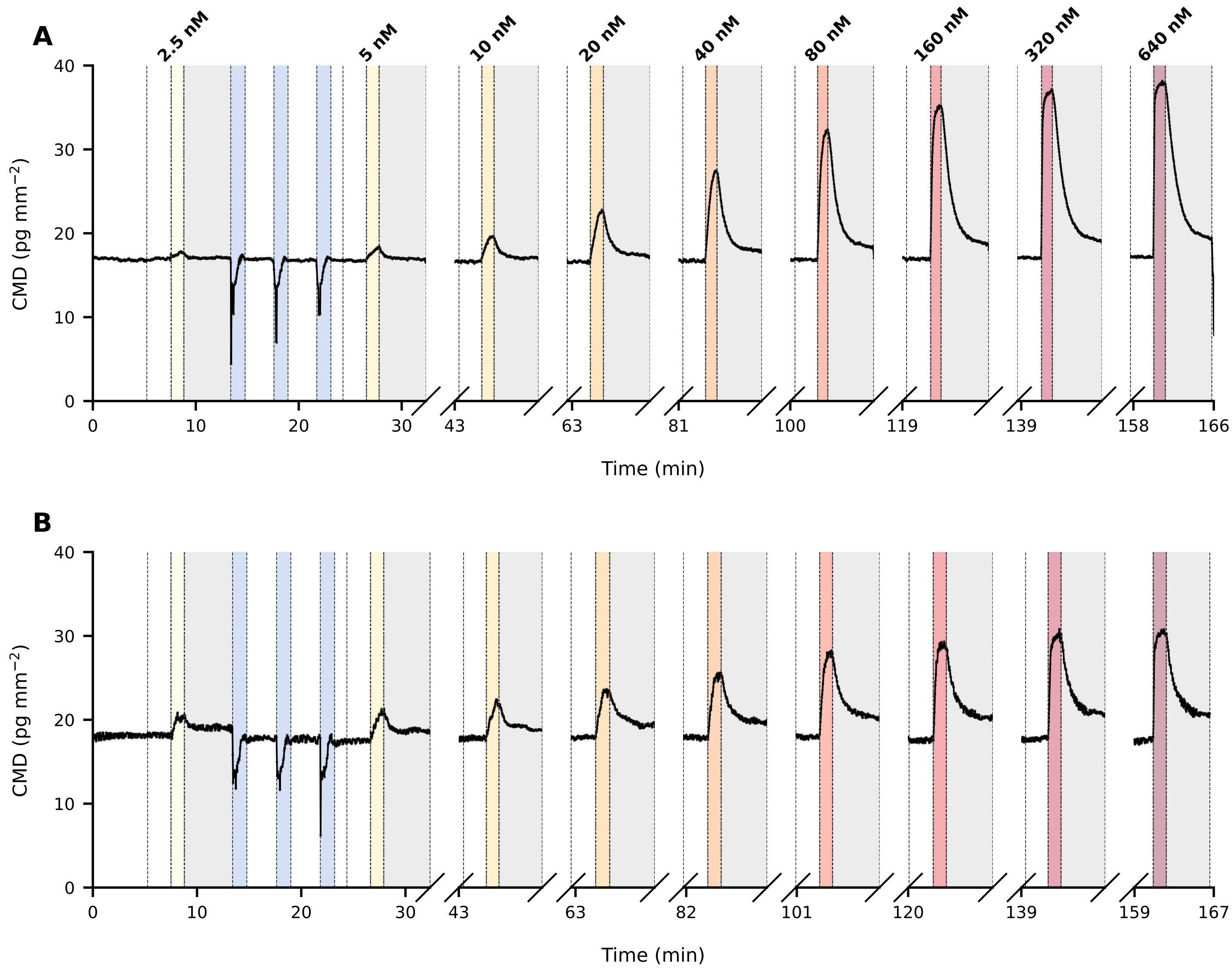

S6 Fig (b). Kinetic experiments in *buffer A* and 50% human serum (Callisto Pre-series reader). Multi-cycle kinetic (MCK) experiments for 6-AH-cAMP at 20°C with PKA-R concentrations from 2.5 nM to 640 nM. Regeneration between cycles used 3 M GuHCl and 125 mM NaOH. **(A)** Titration in *buffer A*. Binding responses across all PKA-R concentrations (highlighted in pink) demonstrate reproducible sensorgrams with complete baseline restoration after regeneration. **(B)** Titration in 50% human serum. Sensorgrams are comparable in shape and absolute signal to those in buffer, supporting detection of PKA-R–cAMP interactions in complex media; the corresponding blank injection showed no detectable non-specific CMD response for the TSH-depleted serum preparation. Minor signal fluctuations are attributed to prototype image processing software.

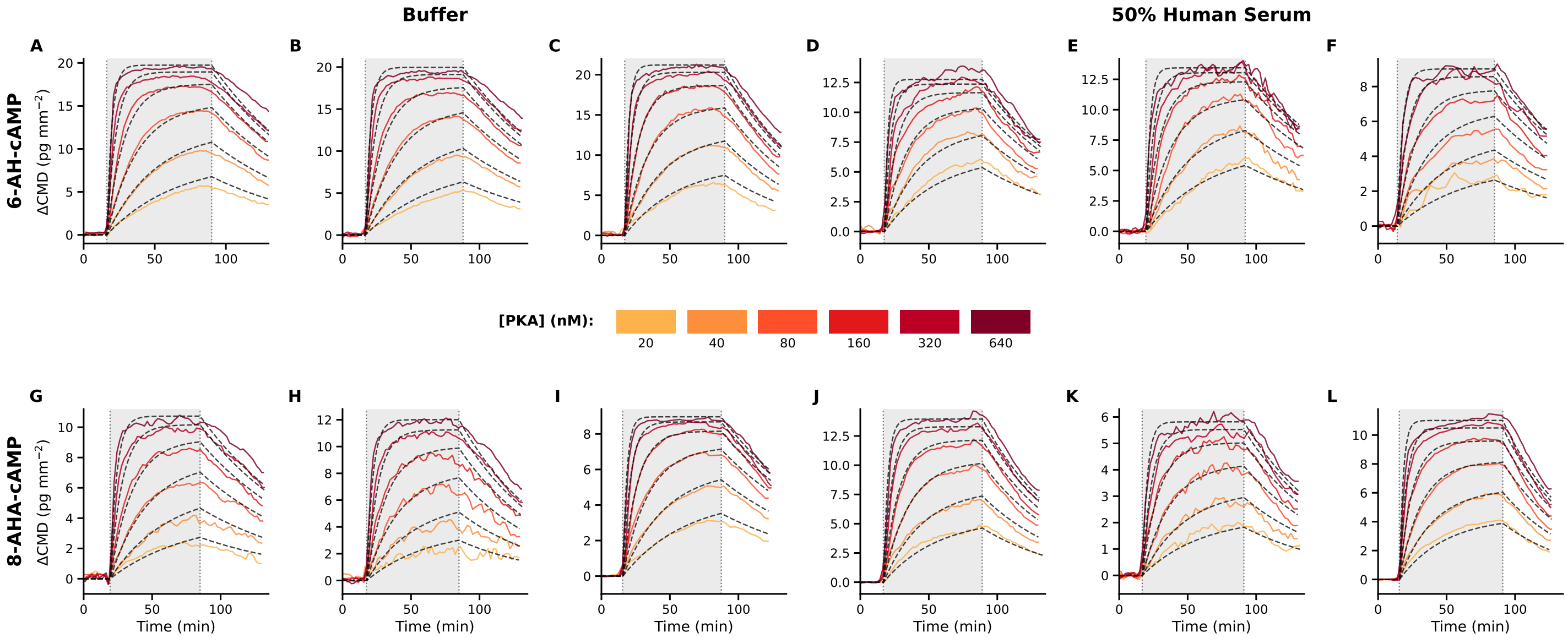

S6 Fig (c). Kinetic fits in *buffer A* and 50% human serum — triplicate measurements (Callisto Pre-series reader). Kinetic fits for 6-AH-cAMP (**A**–**F**) and 8-AHA-cAMP (**G**–**L**) at 20°C in *buffer A* (panels A–C and G–I) and 50% human serum (panels D–F and J–L). Each condition is shown for three independent replicate measurements. Binding data (solid lines) show good agreement with the Langmuir 1:1 binding model (dashed lines). Sensorgrams in buffer and serum may differ in absolute CMD level due to mologram-to-mologram variation in analyte efficiency [5]; this does not affect the extracted kinetic parameters.

S2 Table. Triplicate kinetic measurements in *buffer A* and 50% human serum at 20°C for 6-AH-cAMP and 8-AHA-cAMP (Callisto Pre-series reader).

Ligand	Media	Label	k_{on} [10^5 $\mathbf{M^{-1}s^{-1}}$]	k_{off} [10^{-2} $\mathbf{s^{-1}}$]	K_D [nM]	R_{max} [pg/mm^2]
6-AH-cAMP	Buffer	i)	4.36	1.21	27.8	19.5
		ii)	3.92	1.15	29.3	19.3
		iii)	5.15	1.55	30.3	21.0
	Serum	i)	6.17	1.30	21.1	13.6
		ii)	5.32	1.08	20.4	13.2
		iii)	3.68	1.29	34.9	8.8
8-AHA-cAMP	Buffer	i)	3.12	1.17	37.7	10.5
		ii)	3.34	1.55	46.4	11.8
		iii)	5.20	1.16	22.2	8.8
	Serum	i)	4.77	1.58	33.2	14.0
		ii)	4.24	1.50	35.4	6.0
		iii)	5.61	1.87	33.3	11.1

S3 Table. Mean kinetic parameters with 95% confidence intervals for 6-AH-cAMP and 8-AHA-cAMP in *buffer A* and 50% human serum at 20°C (Callisto Pre-series reader).

**Ligand**	**Media**	k_{on} [10^5 $\mathbf{M^{-1}s^{-1}}$]	k_{off} [10^{-2} $\mathbf{s^{-1}}$]	K_{D} [**nM**]
6-AH-cAMP	Buffer	4.48 (2.93–6.02)	1.30 (0.77–1.84)	29.1 (25.9–32.3)
	Serum	5.06 (1.91–8.20)	1.22 (0.92–1.53)	25.4 (5.1–45.8)
8-AHA-cAMP	Buffer	3.89 (1.05–6.73)	1.29 (0.74–1.85)	35.4 (5.0–65.9)
	Serum	4.87 (3.16–6.59)	1.65 (1.17–2.13)	33.9 (30.9–37.0)

Confidence intervals calculated from three replicates using the t-distribution.

S7. Raw data and sensorgrams for the on-DNA thermodynamic characterization of five cAMP derivatives

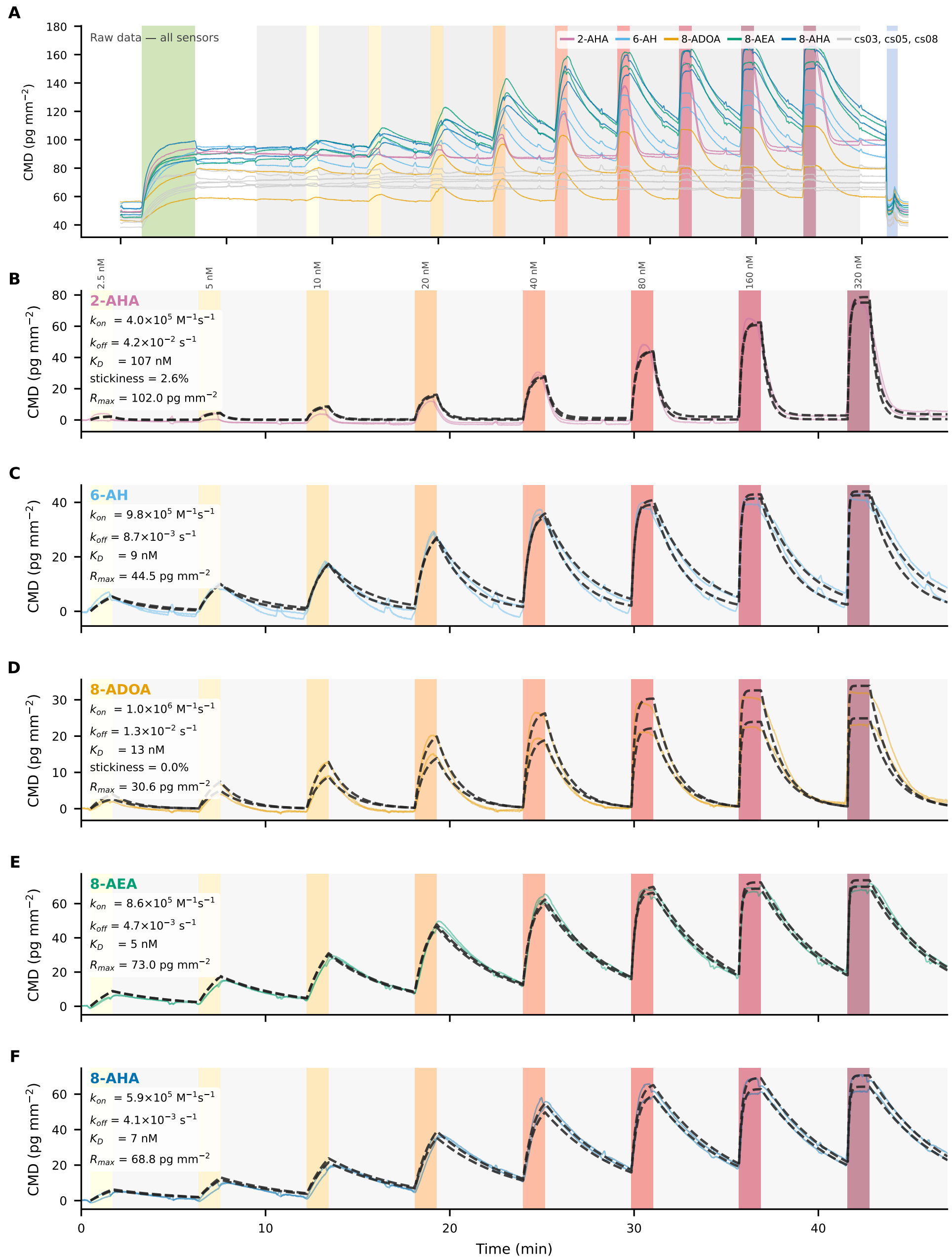

S7 Fig (a) DDI-SCK overview at 15 °C (SCK Copy -2 15deg, SCK cycle 1). **(A)** Raw CMD sensorgrams for all 16 sensors (immobilization, SCK cycle, regeneration). Traces color-coded by compound; gray = control spots (cs03, cs05, cs08). Shading: olive = immobilization, blue = regeneration wash, warm scale = SCK association (low to high concentration), light gray = dissociation. **(B–F)** Per-sensor kinetic traces and Langmuir 1:1 fits (dashed black) at 15 °C; k_{on}, k_{off}, K_D, and $R_{\max}$ annotated in each panel.

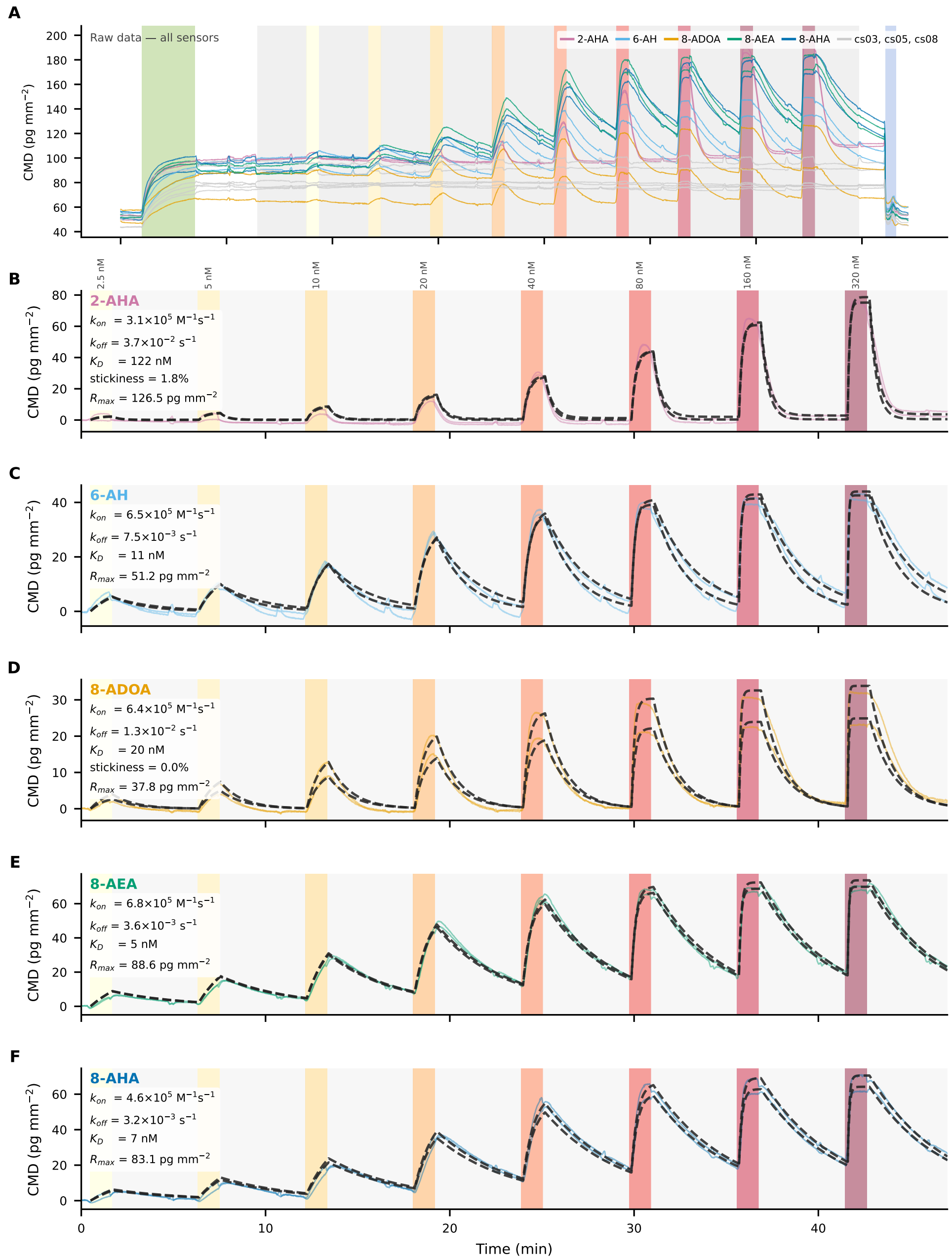

S7 Fig (b) DDI-SCK overview at 15 °C (SCK 15 deg, SCK cycle 1). **(A)** Raw CMD sensorgrams for all 16 sensors (immobilization, SCK cycle, regeneration). Traces color-coded by compound; gray = control spots (cs03, cs05, cs08). Shading: olive = immobilization, blue = regeneration wash, warm scale = SCK association (low to high concentration), light gray = dissociation. **(B–F)** Per-sensor kinetic traces and Langmuir 1:1 fits (dashed black) at 15 °C; k_{on}, k_{off}, K_D, and R_{max} annotated in each panel.

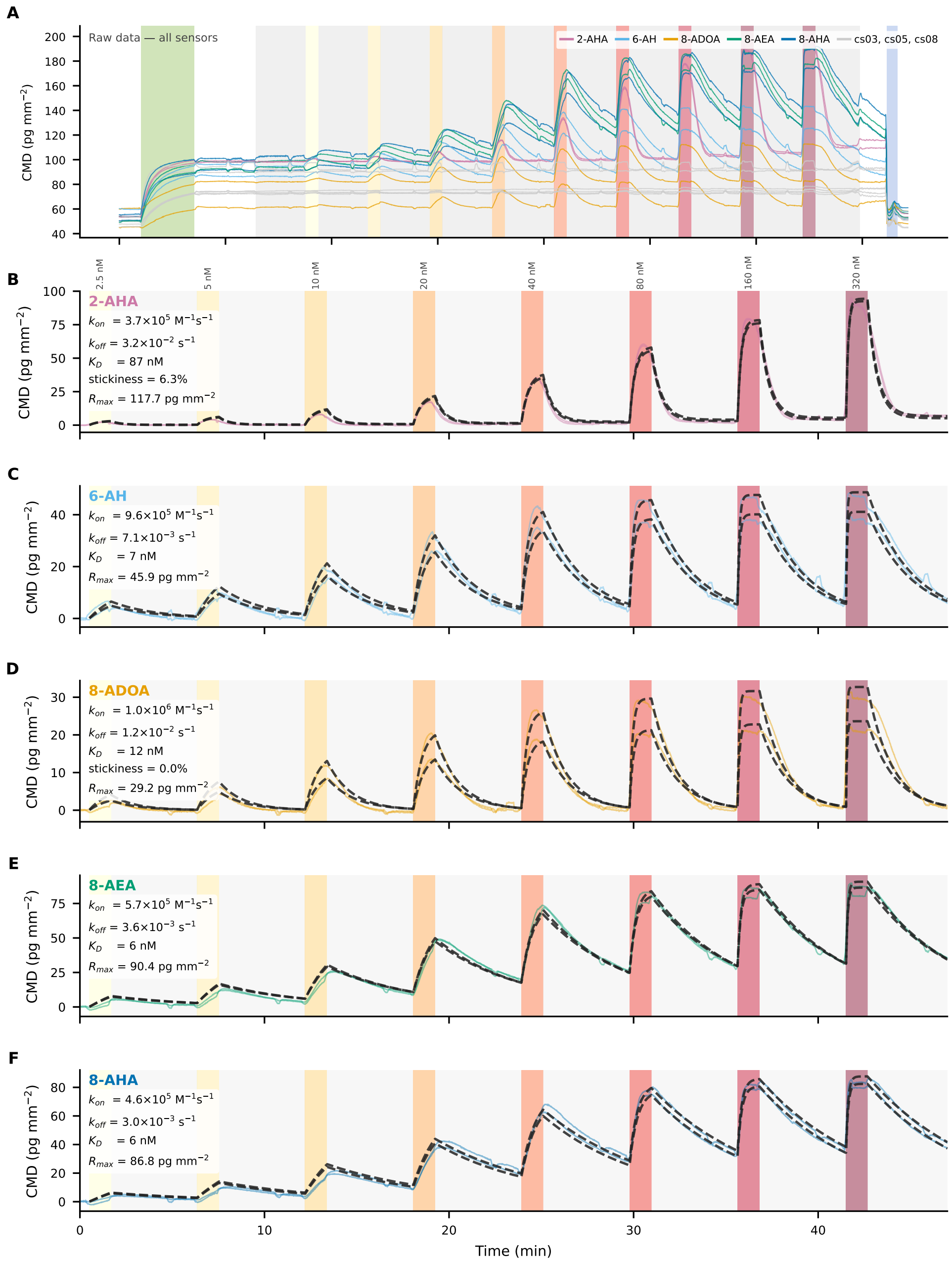

S7 Fig (c) DDI-SCK overview at 15 °C (SCK 15 deg, SCK cycle 2).
(A) Raw CMD sensorgrams for all 16 sensors (immobilization, SCK cycle, regeneration). Traces color-coded by compound; gray = control spots (cs03, cs05, cs08). Shading: olive = immobilization, blue = regeneration wash, warm scale = SCK association (low to high concentration), light gray = dissociation. **(B–F)** Per-sensor kinetic traces and Langmuir 1:1 fits (dashed black) at 15 °C; k_{on}, k_{off}, K_D, and $R_{\max}$ annotated in each panel.

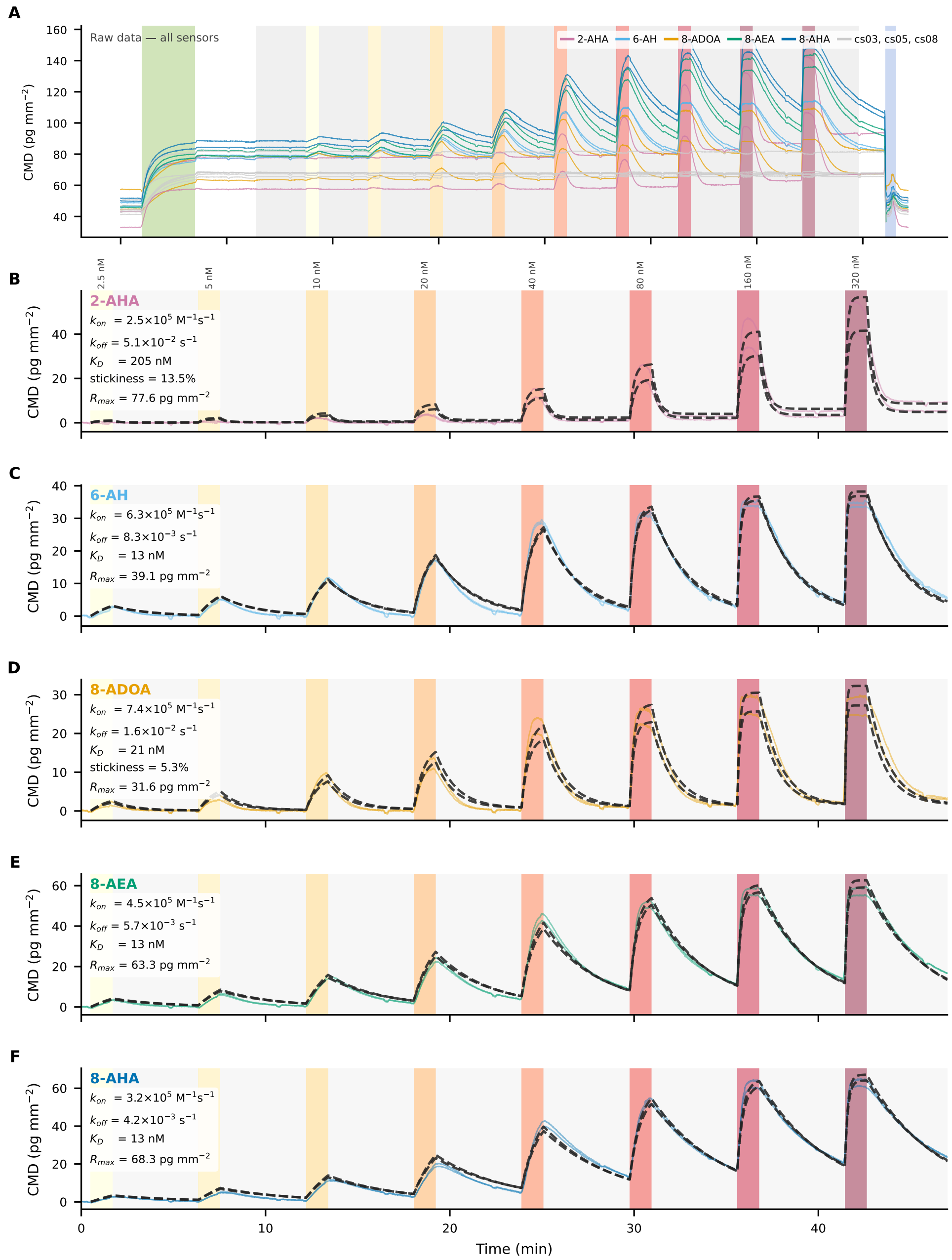

S7 Fig (d) DDI-SCK overview at 15 °C (SCK 3 combined 25 15 30 deg-1, SCK cycle 2). (A) Raw CMD sensorgrams for all 16 sensors (immobilization, SCK cycle, regeneration). Traces color-coded by compound; gray = control spots (cs03, cs05, cs08). Shading: olive = immobilization, blue = regeneration wash, warm scale = SCK association (low to high concentration), light gray = dissociation. **(B–F)** Per-sensor kinetic traces and Langmuir 1:1 fits (dashed black) at 15 °C; k_{on}, k_{off}, K_D, and $R_{\max}$ annotated in each panel.

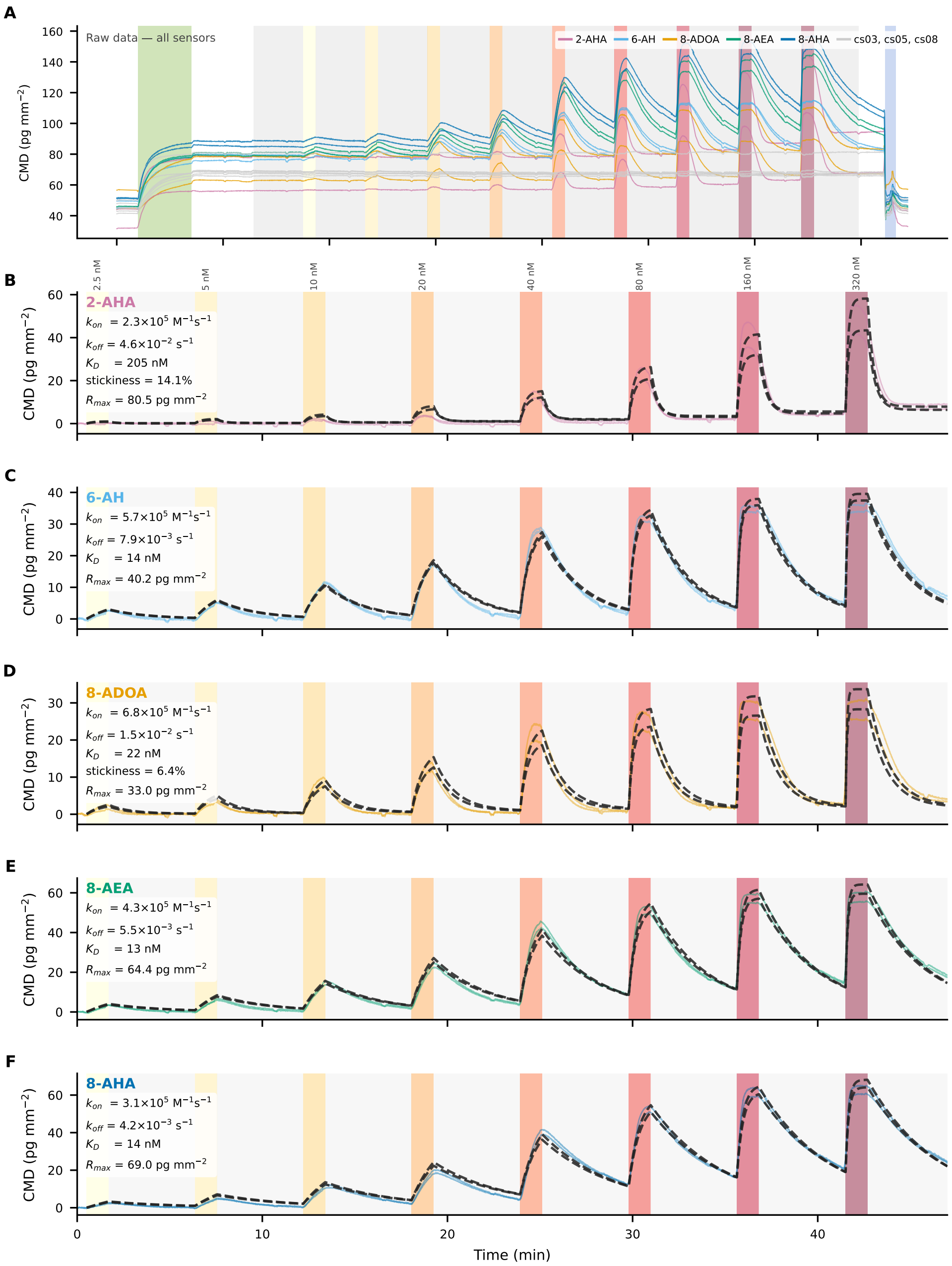

S7 Fig (e) DDI-SCK overview at 15 °C (SCK 3 combined 25 15 30 deg-1, SCK cycle 1). (A) Raw CMD sensorgrams for all 16 sensors (immobilization, SCK cycle, regeneration). Traces color-coded by compound; gray = control spots (cs03, cs05, cs08). Shading: olive = immobilization, blue = regeneration wash, warm scale = SCK association (low to high concentration), light gray = dissociation. **(B–F)** Per-sensor kinetic traces and Langmuir 1:1 fits (dashed black) at 15 °C; k_{on}, k_{off}, K_D, and $R_{\max}$ annotated in each panel.

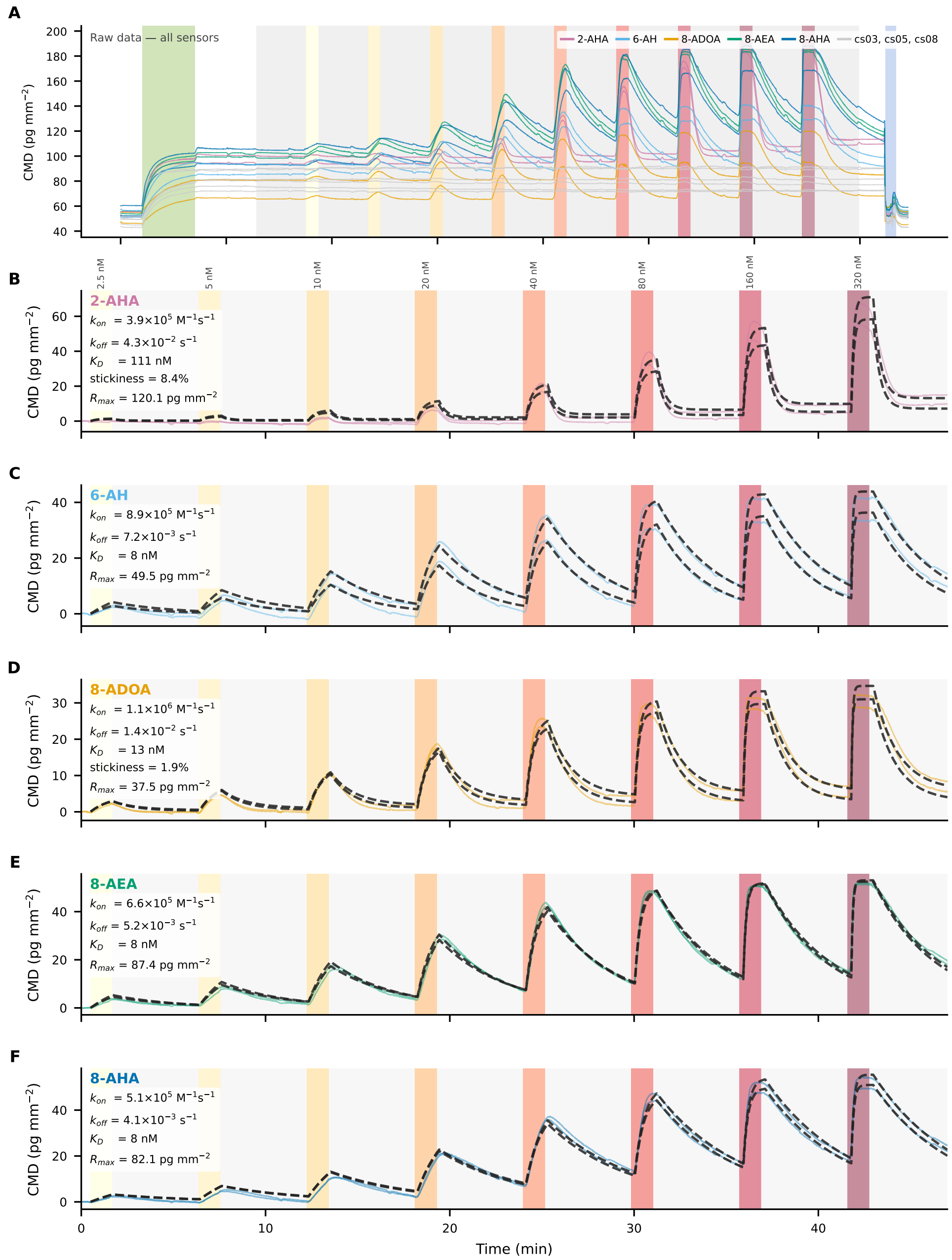

S7 Fig (f) DDI-SCK overview at 20 °C (SCK 3 cycles other way-1 20deg, SCK cycle 1). (A) Raw CMD sensorgrams for all 16 sensors (immobilization, SCK cycle, regeneration). Traces color-coded by compound; gray = control spots (cs03, cs05, cs08). Shading: olive = immobilization, blue = regeneration wash, warm scale = SCK association (low to high concentration), light gray = dissociation. **(B–F)** Per-sensor kinetic traces and Langmuir 1:1 fits (dashed black) at 20 °C; k_{on}, k_{off}, K_D, and $R_{\max}$ annotated in each panel.

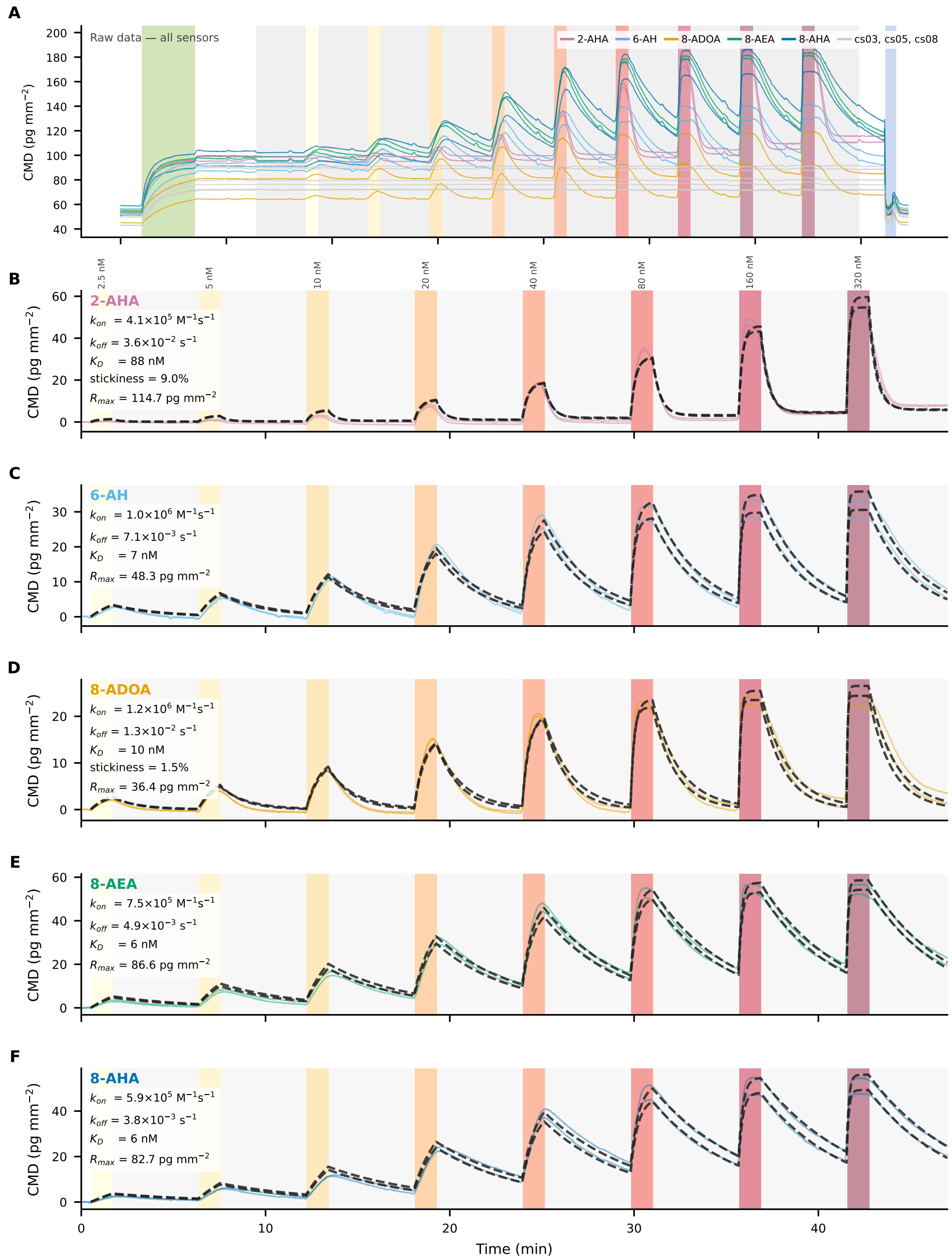

S7 Fig (g) DDI-SCK overview at 20 °C (SCK 3 cycles other way-1 20deg, SCK cycle 2). (A) Raw CMD sensorgrams for all 16 sensors (immobilization, SCK cycle, regeneration). Traces color-coded by compound; gray = control spots (cs03, cs05, cs08). Shading: olive = immobilization, blue = regeneration wash, warm scale = SCK association (low to high concentration), light gray = dissociation. **(B–F)** Per-sensor kinetic traces and Langmuir 1:1 fits (dashed black) at 20 °C; k_{on}, k_{off}, K_D, and $R_{\max}$ annotated in each panel.

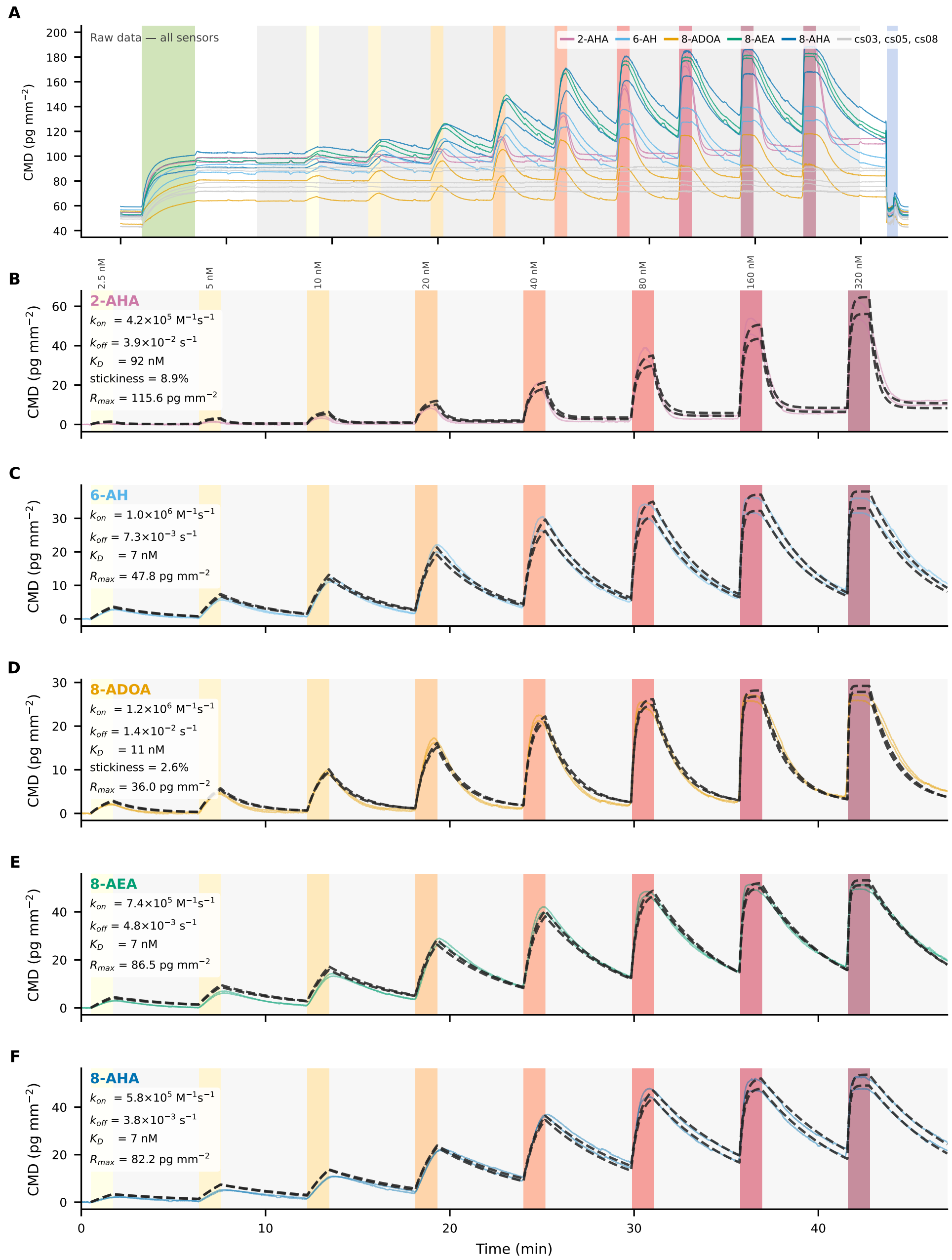

S7 Fig (h) DDI-SCK overview at 20 °C (SCK 3 cycles other way-1 20deg, SCK cycle 3). (A) Raw CMD sensorgrams for all 16 sensors (immobilization, SCK cycle, regeneration). Traces color-coded by compound; gray = control spots (cs03, cs05, cs08). Shading: olive = immobilization, blue = regeneration wash, warm scale = SCK association (low to high concentration), light gray = dissociation. **(B–F)** Per-sensor kinetic traces and Langmuir 1:1 fits (dashed black) at 20 °C; k_{on}, k_{off}, K_D, and $R_{\max}$ annotated in each panel.

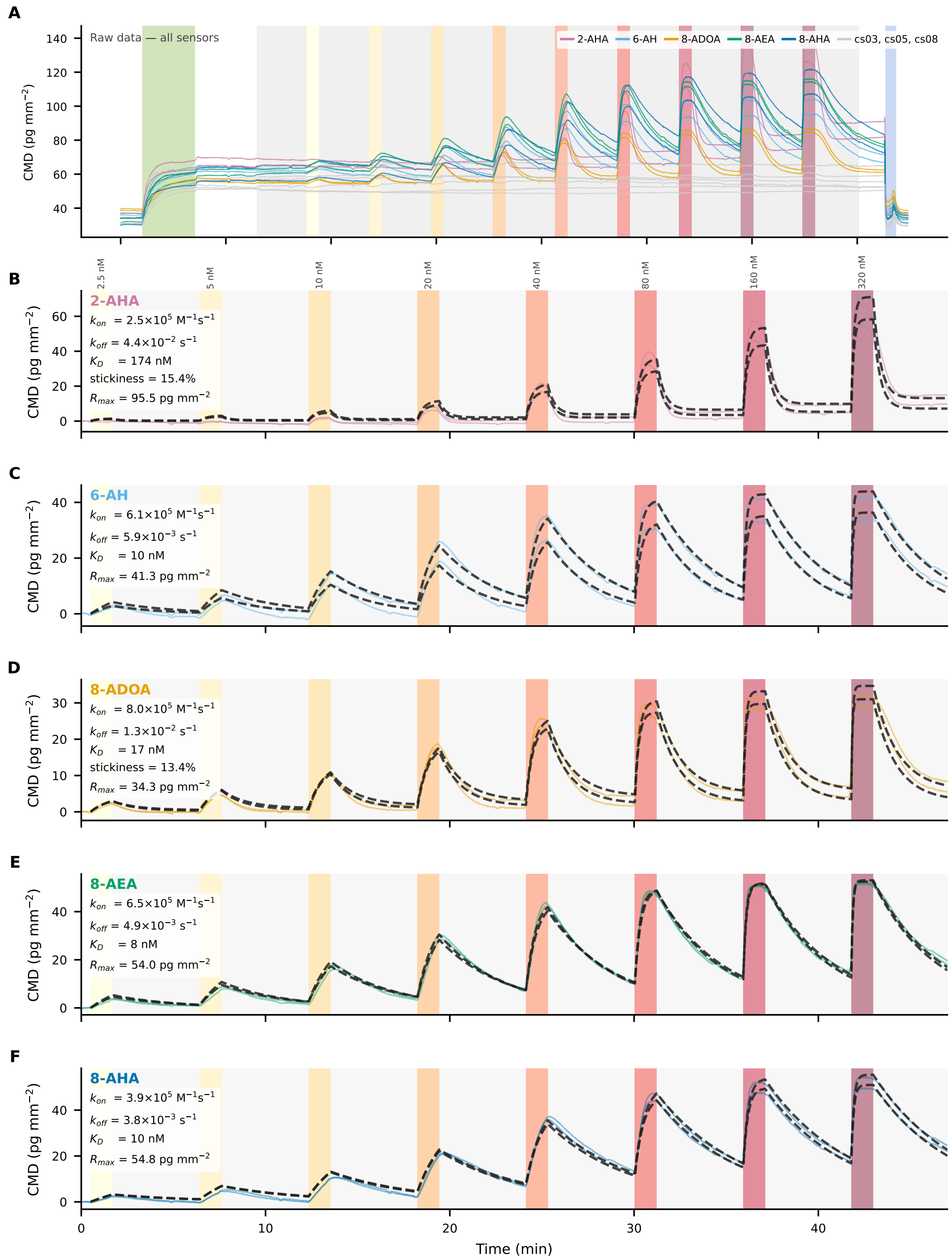

S7 Fig (i) DDI-SCK overview at 20 °C (SCK 3 cycles other way-2, SCK cycle 1). (A) Raw CMD sensorgrams for all 16 sensors (immobilization, SCK cycle, regeneration). Traces color-coded by compound; gray = control spots (cs03, cs05, cs08). Shading: olive = immobilization, blue = regeneration wash, warm scale = SCK association (low to high concentration), light gray = dissociation. **(B–F)** Per-sensor kinetic traces and Langmuir 1:1 fits (dashed black) at 20 °C; k_{on}, k_{off}, K_D, and $R_{\max}$ annotated in each panel.

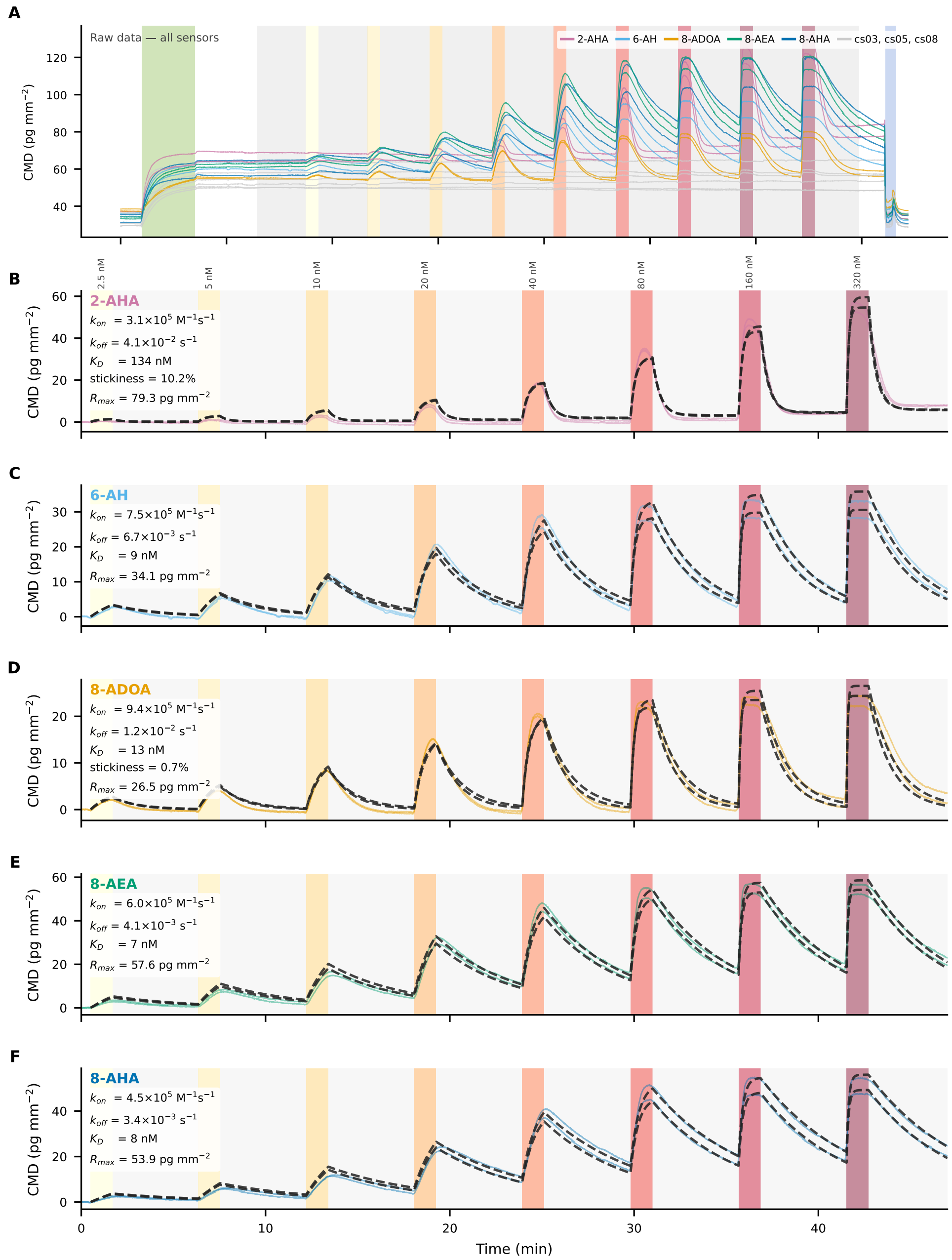

S7 Fig (j) DDI-SCK overview at 20 °C (SCK 3 cycles other way-2, SCK cycle 2). (A) Raw CMD sensorgrams for all 16 sensors (immobilization, SCK cycle, regeneration). Traces color-coded by compound; gray = control spots (cs03, cs05, cs08). Shading: olive = immobilization, blue = regeneration wash, warm scale = SCK association (low to high concentration), light gray = dissociation. **(B–F)** Per-sensor kinetic traces and Langmuir 1:1 fits (dashed black) at 20 °C; k_{on}, k_{off}, K_D, and R_{max} annotated in each panel.

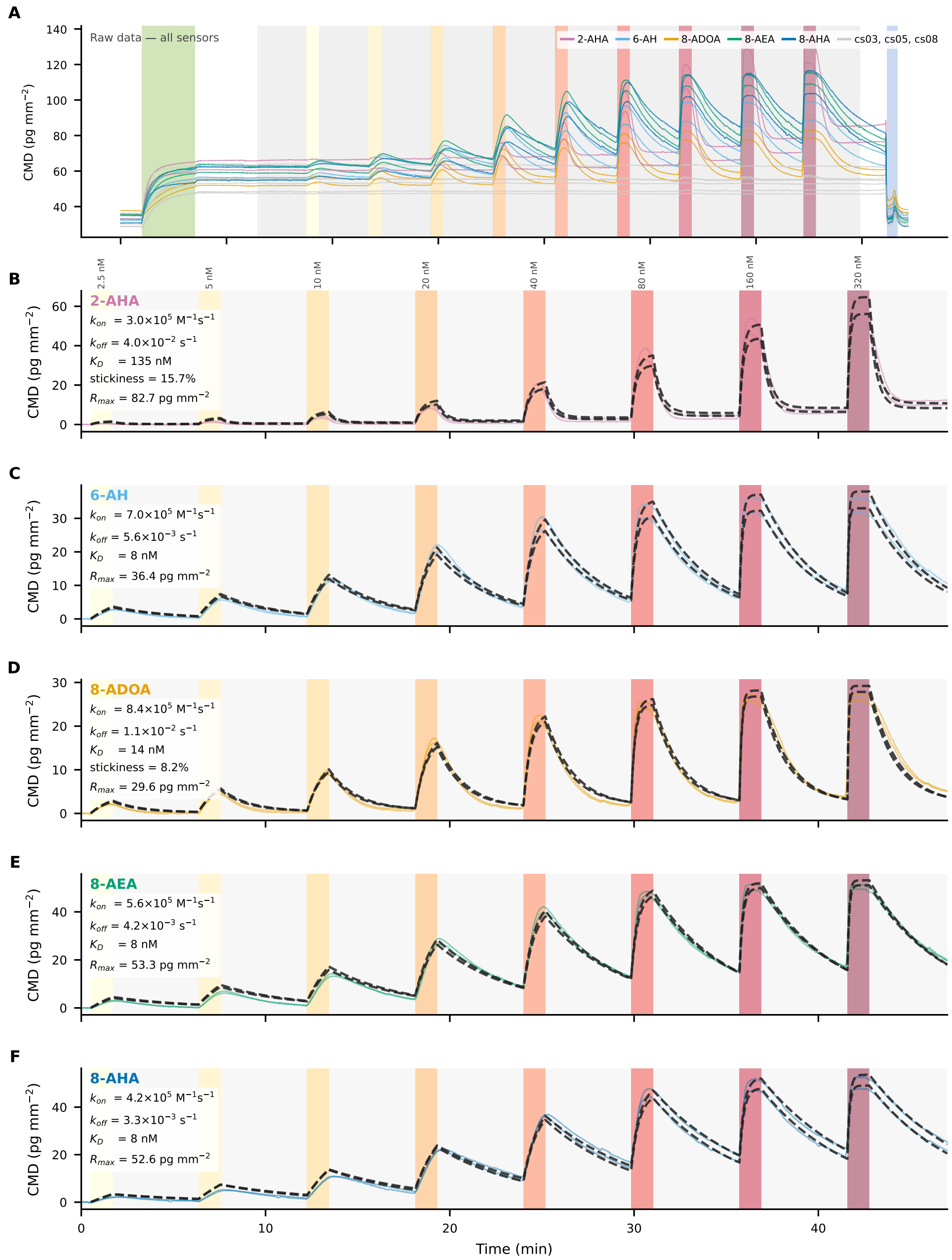

S7 Fig (k) DDI-SCK overview at 20 °C (SCK 3 cycles other way-2, SCK cycle 3). (A) Raw CMD sensorgrams for all 16 sensors (immobilization, SCK cycle, regeneration). Traces color-coded by compound; gray = control spots (cs03, cs05, cs08). Shading: olive = immobilization, blue = regeneration wash, warm scale = SCK association (low to high concentration), light gray = dissociation. **(B–F)** Per-sensor kinetic traces and Langmuir 1:1 fits (dashed black) at 20 °C; k_{on}, k_{off}, K_D, and R_{max} annotated in each panel.

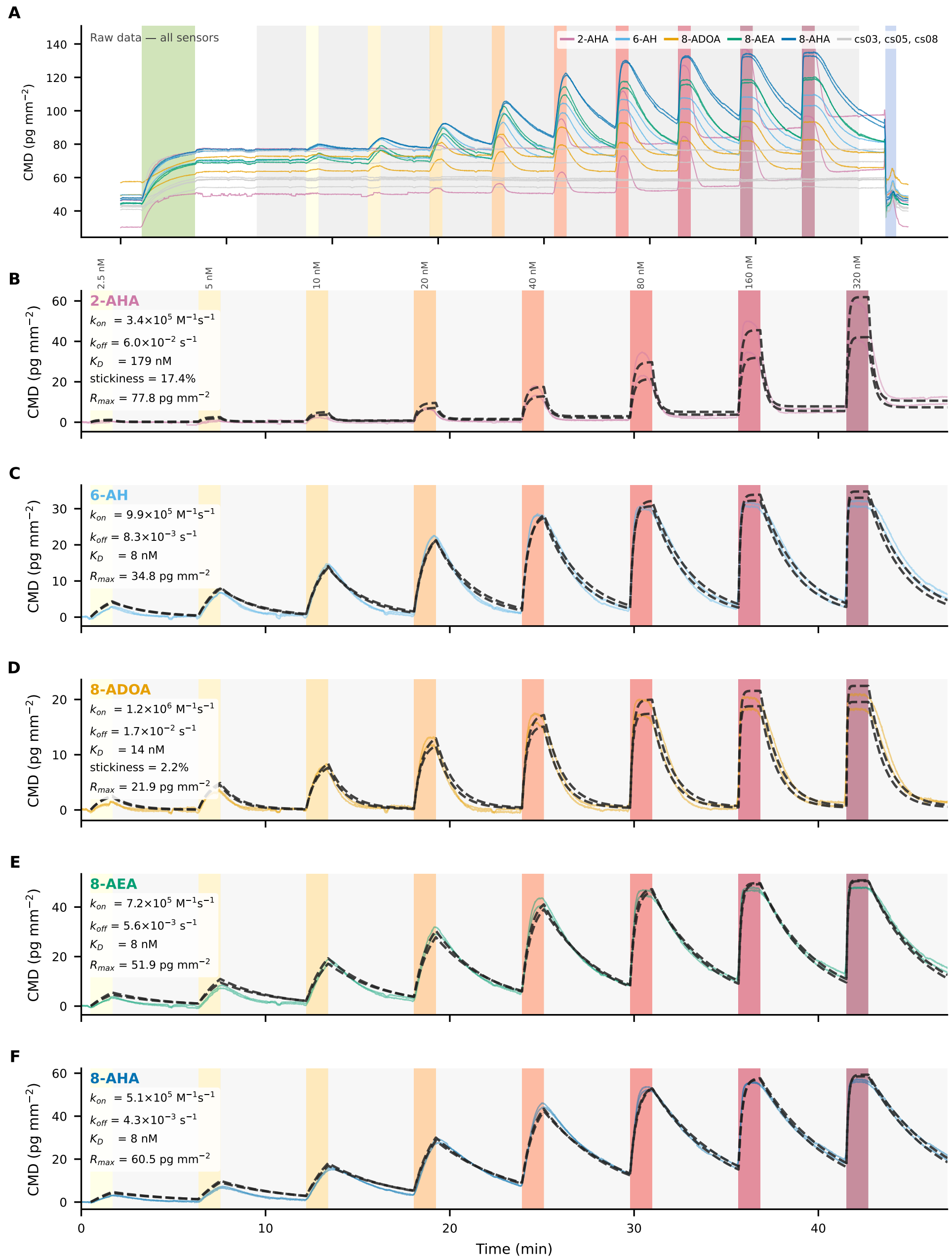

S7 Fig (l) DDI-SCK overview at 20 °C (SCK 3 cycles other way 20deg, SCK cycle 1). (A) Raw CMD sensorgrams for all 16 sensors (immobilization, SCK cycle, regeneration). Traces color-coded by compound; gray = control spots (cs03, cs05, cs08). Shading: olive = immobilization, blue = regeneration wash, warm scale = SCK association (low to high concentration), light gray = dissociation. **(B–F)** Per-sensor kinetic traces and Langmuir 1:1 fits (dashed black) at 20 °C; k_{on}, k_{off}, K_D, and R_{max} annotated in each panel.

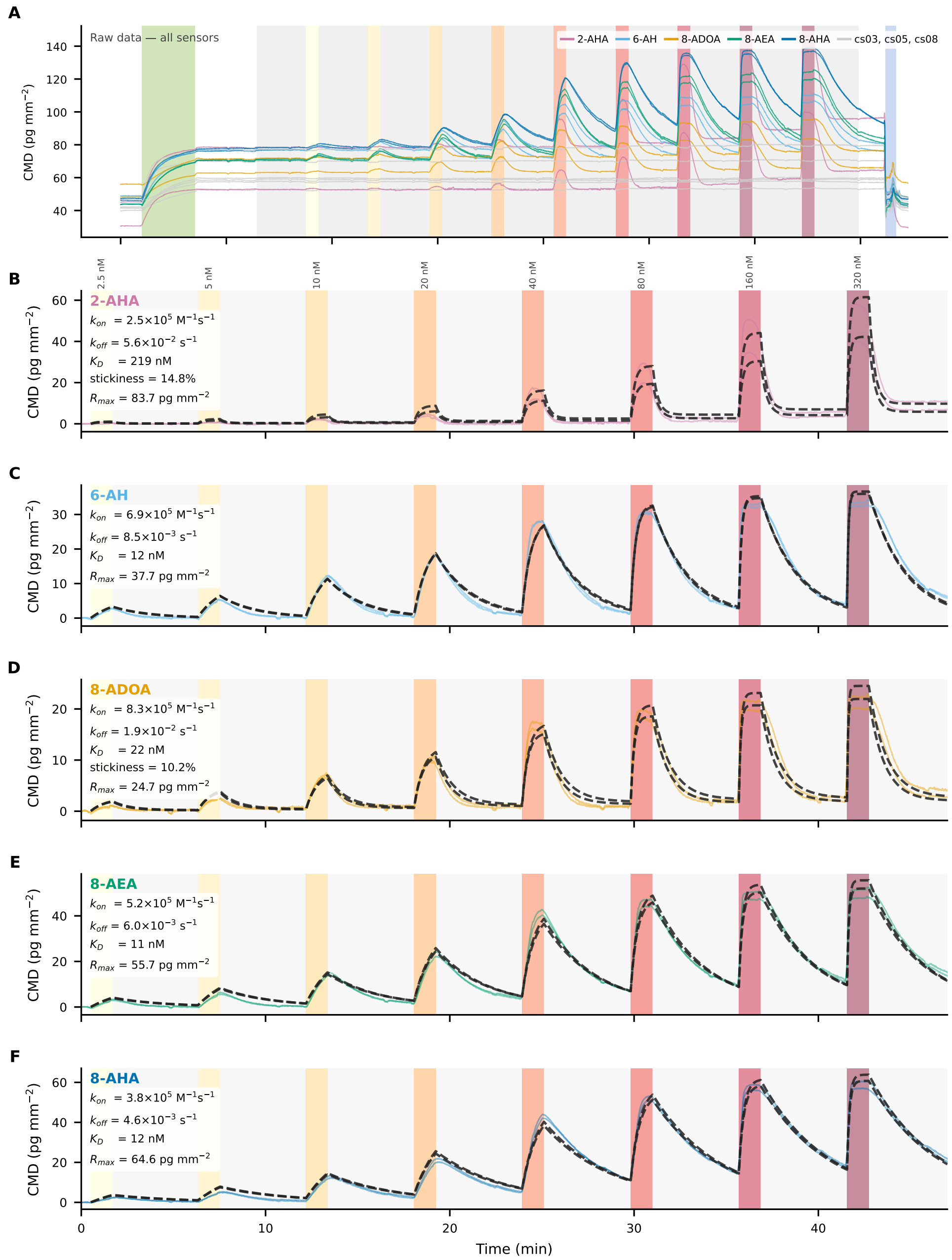

S7 Fig (m) DDI-SCK overview at 20 °C (SCK 3 cycles other way 20deg, SCK cycle 2). (A) Raw CMD sensorgrams for all 16 sensors (immobilization, SCK cycle, regeneration). Traces color-coded by compound; gray = control spots (cs03, cs05, cs08). Shading: olive = immobilization, blue = regeneration wash, warm scale = SCK association (low to high concentration), light gray = dissociation. **(B–F)** Per-sensor kinetic traces and Langmuir 1:1 fits (dashed black) at 20 °C; k_{on}, k_{off}, K_D, and $R_{\max}$ annotated in each panel.

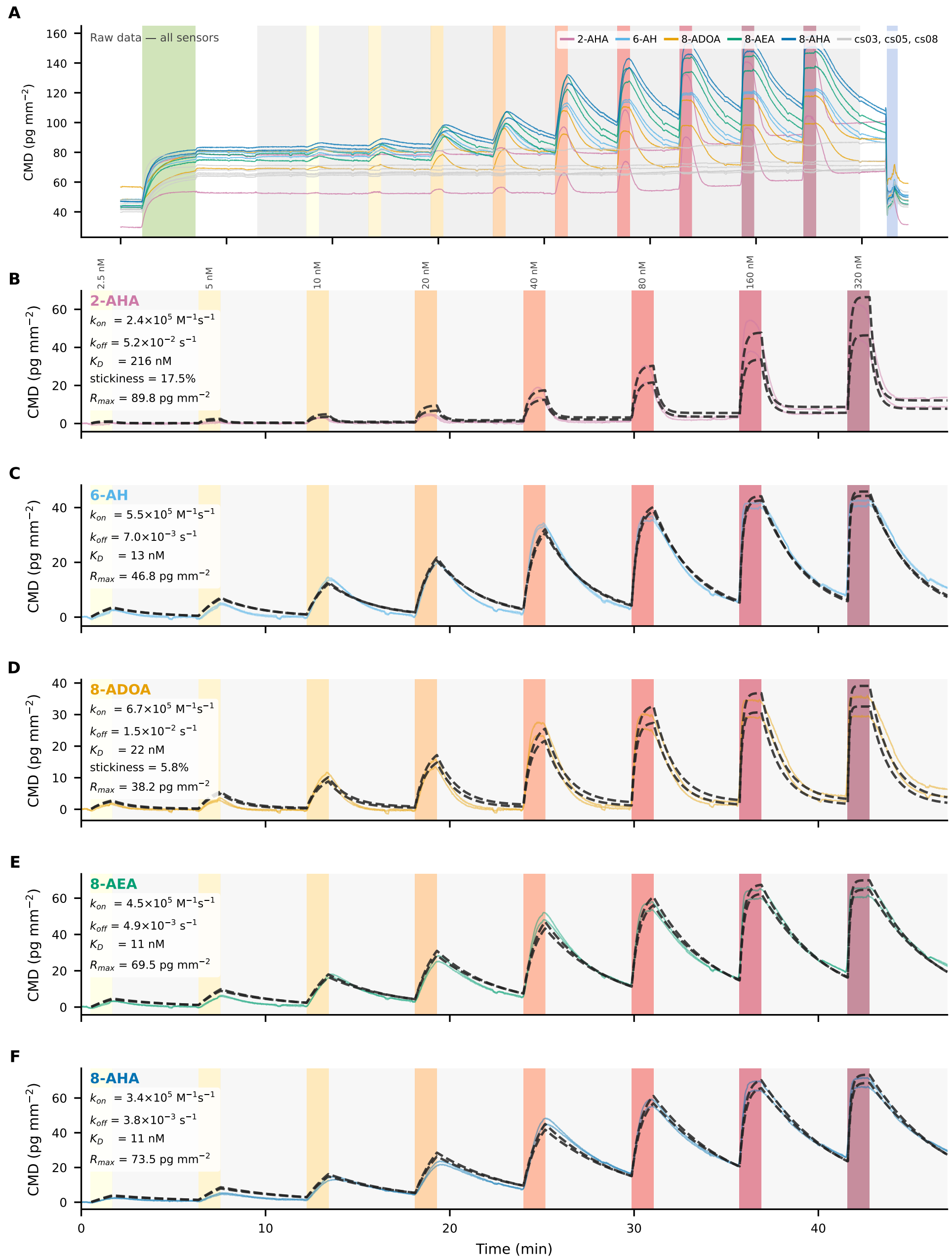

S7 Fig (n) DDI-SCK overview at 20 °C (SCK 3 cycles other way 20deg, SCK cycle 3). (A) Raw CMD sensorgrams for all 16 sensors (immobilization, SCK cycle, regeneration). Traces color-coded by compound; gray = control spots (cs03, cs05, cs08). Shading: olive = immobilization, blue = regeneration wash, warm scale = SCK association (low to high concentration), light gray = dissociation. **(B–F)** Per-sensor kinetic traces and Langmuir 1:1 fits (dashed black) at 20 °C; k_{on}, k_{off}, K_D, and $R_{\max}$ annotated in each panel.

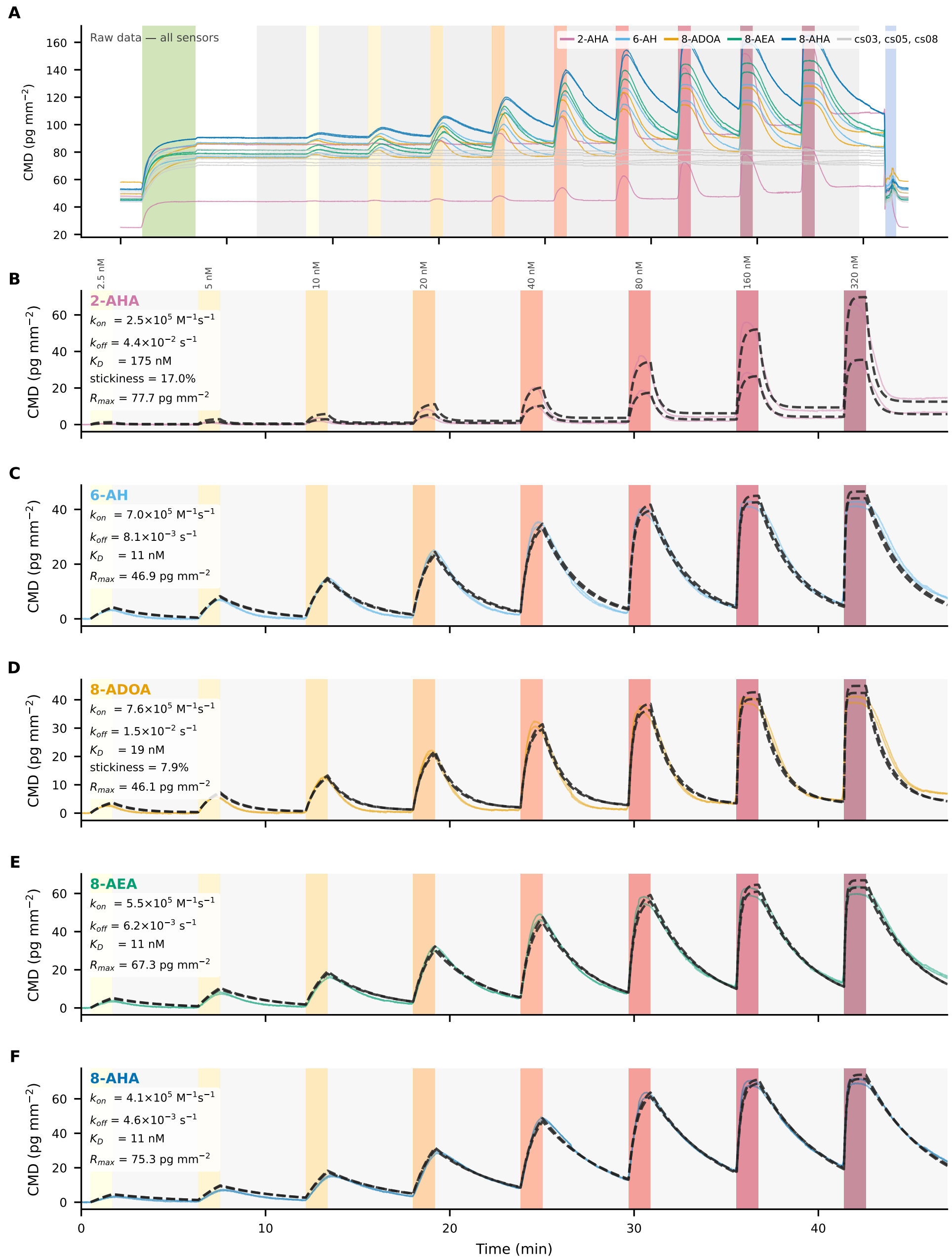

S7 Fig (o) DDI-SCK overview at 25 °C (SCK 3 combined 25 15 30 deg-1, SCK cycle 2). (A) Raw CMD sensorgrams for all 16 sensors (immobilization, SCK cycle, regeneration). Traces color-coded by compound; gray = control spots (cs03, cs05, cs08). Shading: olive = immobilization, blue = regeneration wash, warm scale = SCK association (low to high concentration), light gray = dissociation. **(B–F)** Per-sensor kinetic traces and Langmuir 1:1 fits (dashed black) at 25 °C; k_{on}, k_{off}, K_D, and $R_{\max}$ annotated in each panel.

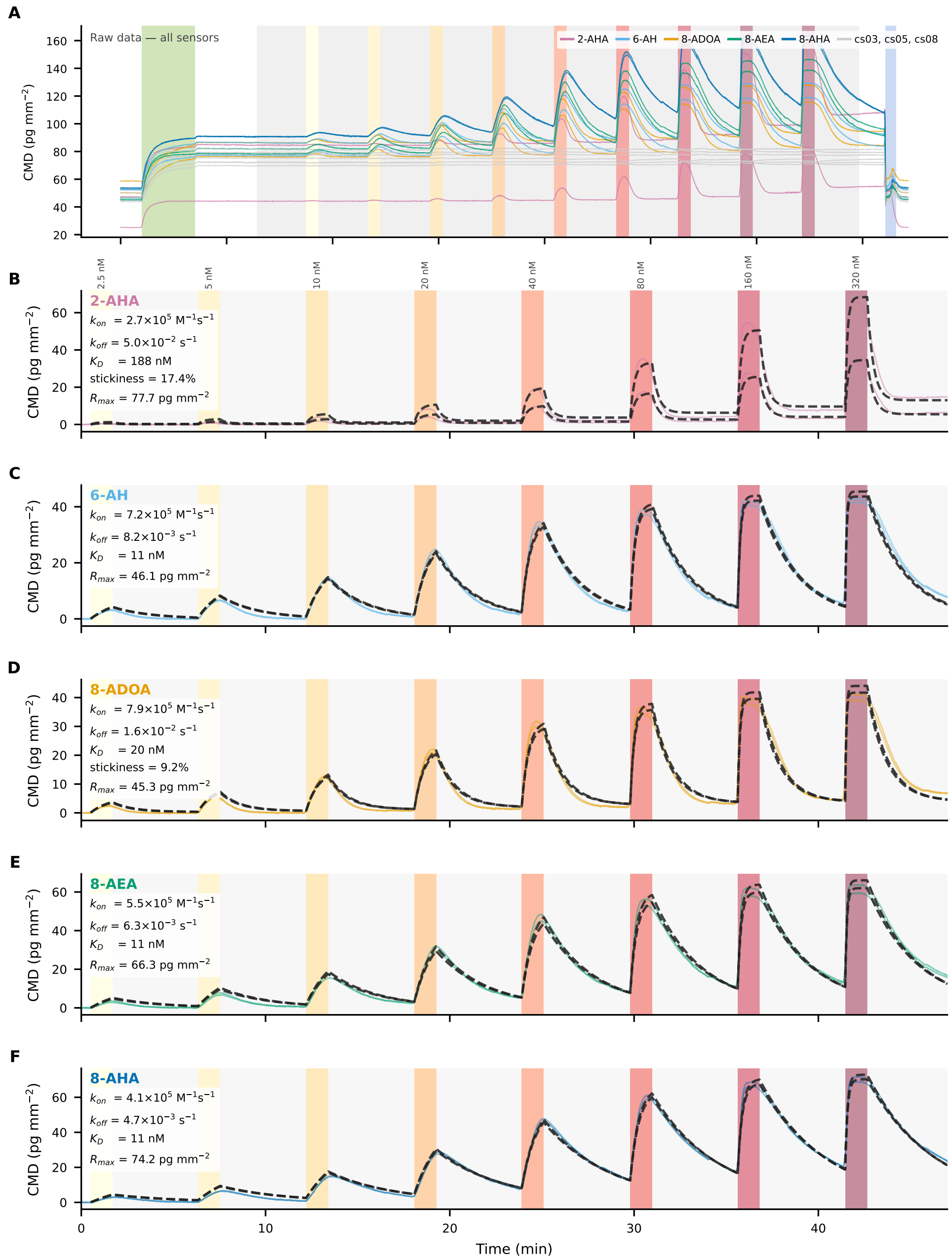

S7 Fig (p) DDI-SCK overview at 25 °C (SCK 3 combined 25 15 30 deg-1, SCK cycle 3). (A) Raw CMD sensorgrams for all 16 sensors (immobilization, SCK cycle, regeneration). Traces color-coded by compound; gray = control spots (cs03, cs05, cs08). Shading: olive = immobilization, blue = regeneration wash, warm scale = SCK association (low to high concentration), light gray = dissociation. **(B–F)** Per-sensor kinetic traces and Langmuir 1:1 fits (dashed black) at 25 °C; k_{on}, k_{off}, K_D, and $R_{\max}$ annotated in each panel.

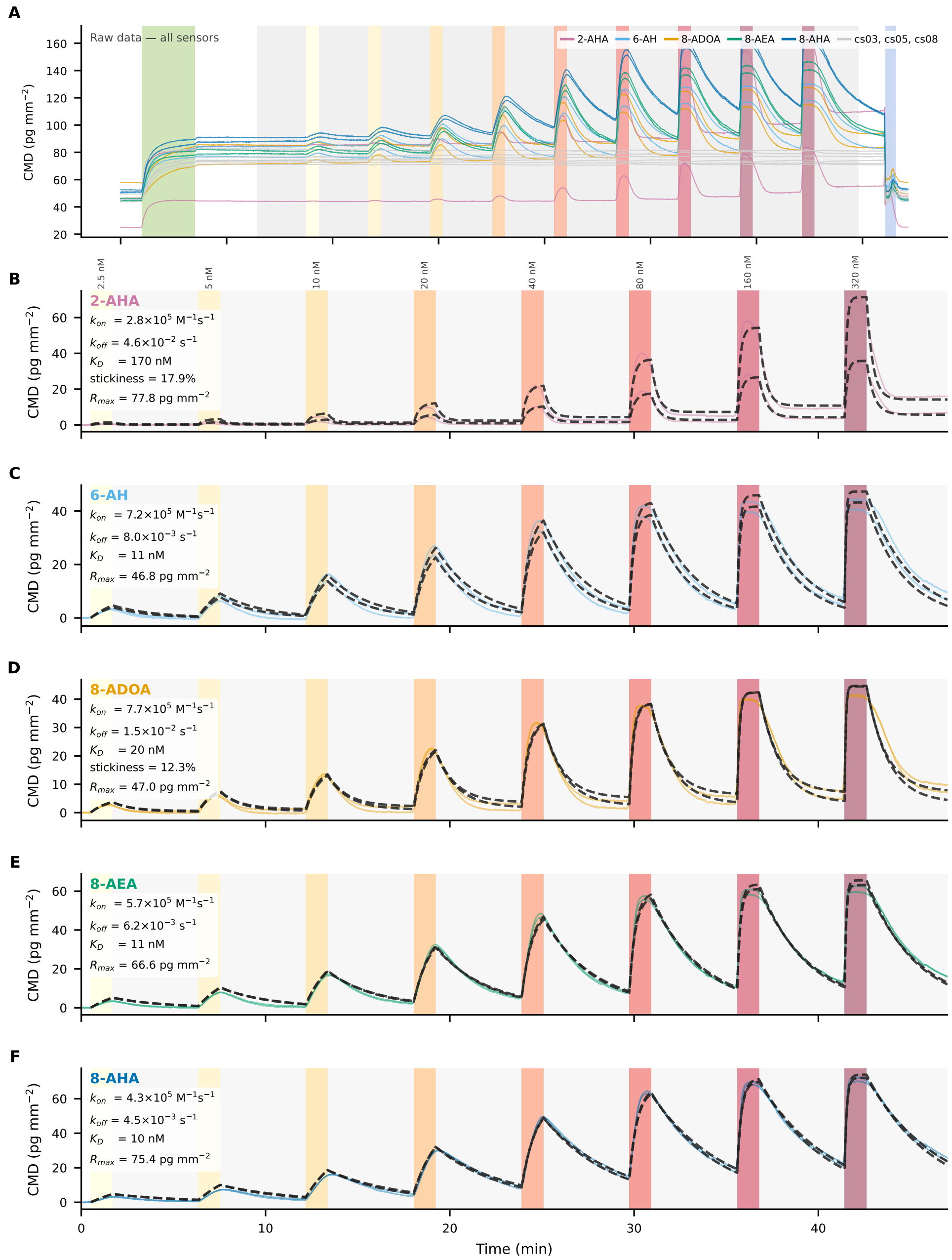

S7 Fig (q) DDI-SCK overview at 25 °C (SCK 3 combined 25 15 30 deg-1, SCK cycle 1). (A) Raw CMD sensorgrams for all 16 sensors (immobilization, SCK cycle, regeneration). Traces color-coded by compound; gray = control spots (cs03, cs05, cs08). Shading: olive = immobilization, blue = regeneration wash, warm scale = SCK association (low to high concentration), light gray = dissociation. **(B–F)** Per-sensor kinetic traces and Langmuir 1:1 fits (dashed black) at 25 °C; k_{on}, k_{off}, K_D, and $R_{\max}$ annotated in each panel.

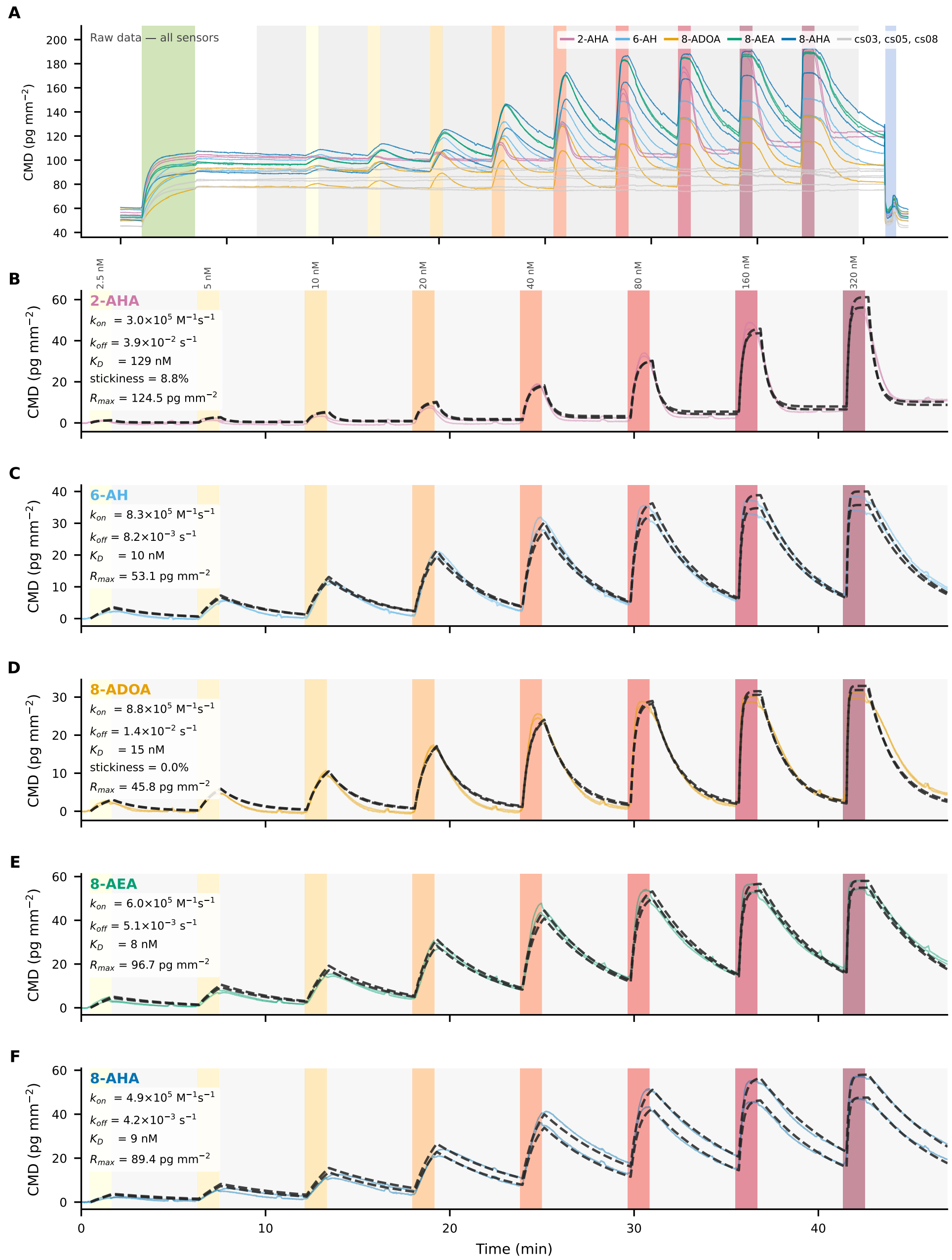

S7 Fig (r) DDI-SCK overview at 25 °C (SCK 3 cycles-1 25deg, SCK cycle 1). (A) Raw CMD sensorgrams for all 16 sensors (immobilization, SCK cycle, regeneration). Traces color-coded by compound; gray = control spots (cs03, cs05, cs08). Shading: olive = immobilization, blue = regeneration wash, warm scale = SCK association (low to high concentration), light gray = dissociation. **(B–F)** Per-sensor kinetic traces and Langmuir 1:1 fits (dashed black) at 25 °C; k_{on}, k_{off}, K_D, and R_{max} annotated in each panel.

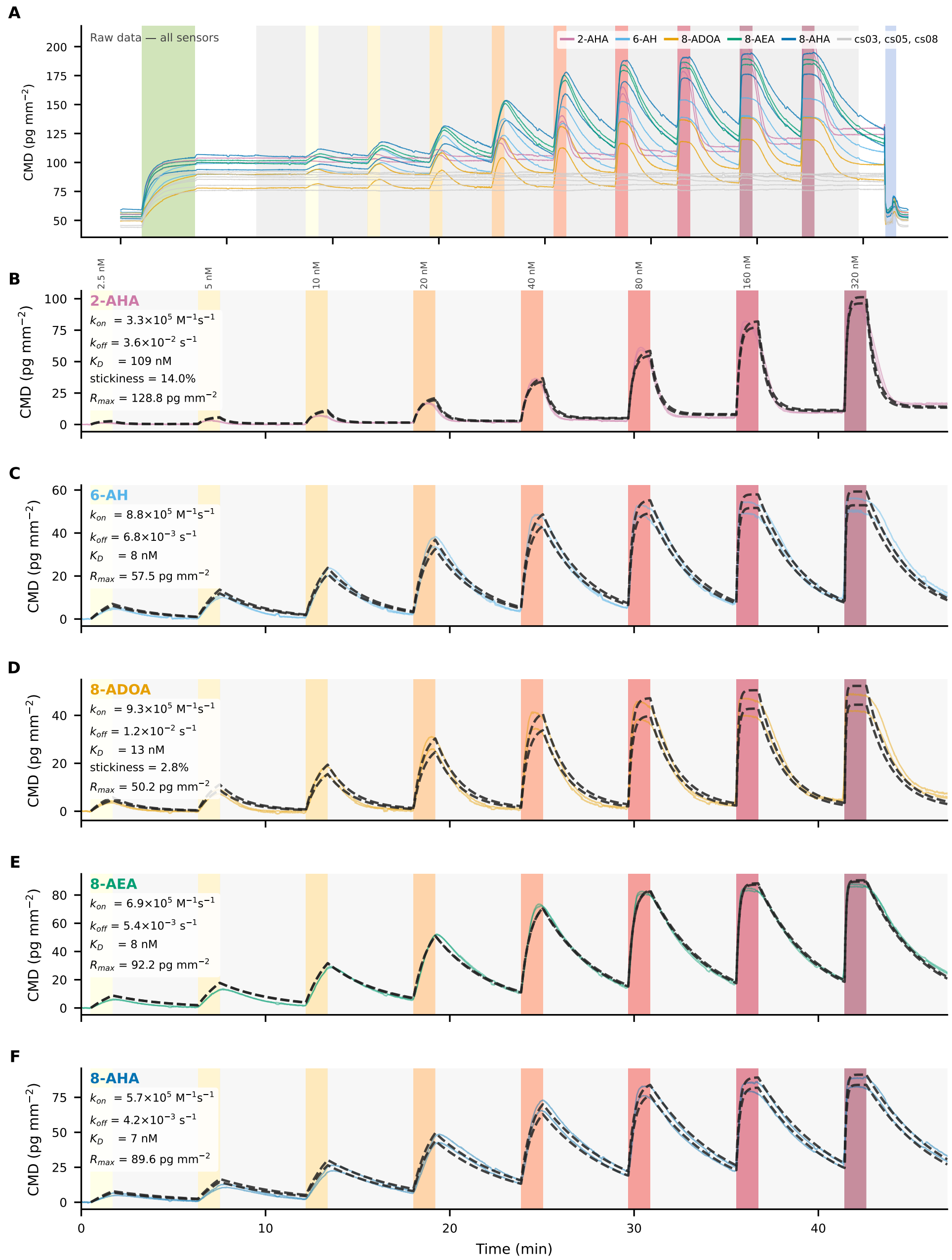

S7 Fig (s) DDI-SCK overview at 25 °C (SCK 3 cycles-1 25deg, SCK cycle 2). (A) Raw CMD sensorgrams for all 16 sensors (immobilization, SCK cycle, regeneration). Traces color-coded by compound; gray = control spots (cs03, cs05, cs08). Shading: olive = immobilization, blue = regeneration wash, warm scale = SCK association (low to high concentration), light gray = dissociation. **(B–F)** Per-sensor kinetic traces and Langmuir 1:1 fits (dashed black) at 25 °C; k_{on}, k_{off}, K_D, and $R_{\max}$ annotated in each panel.

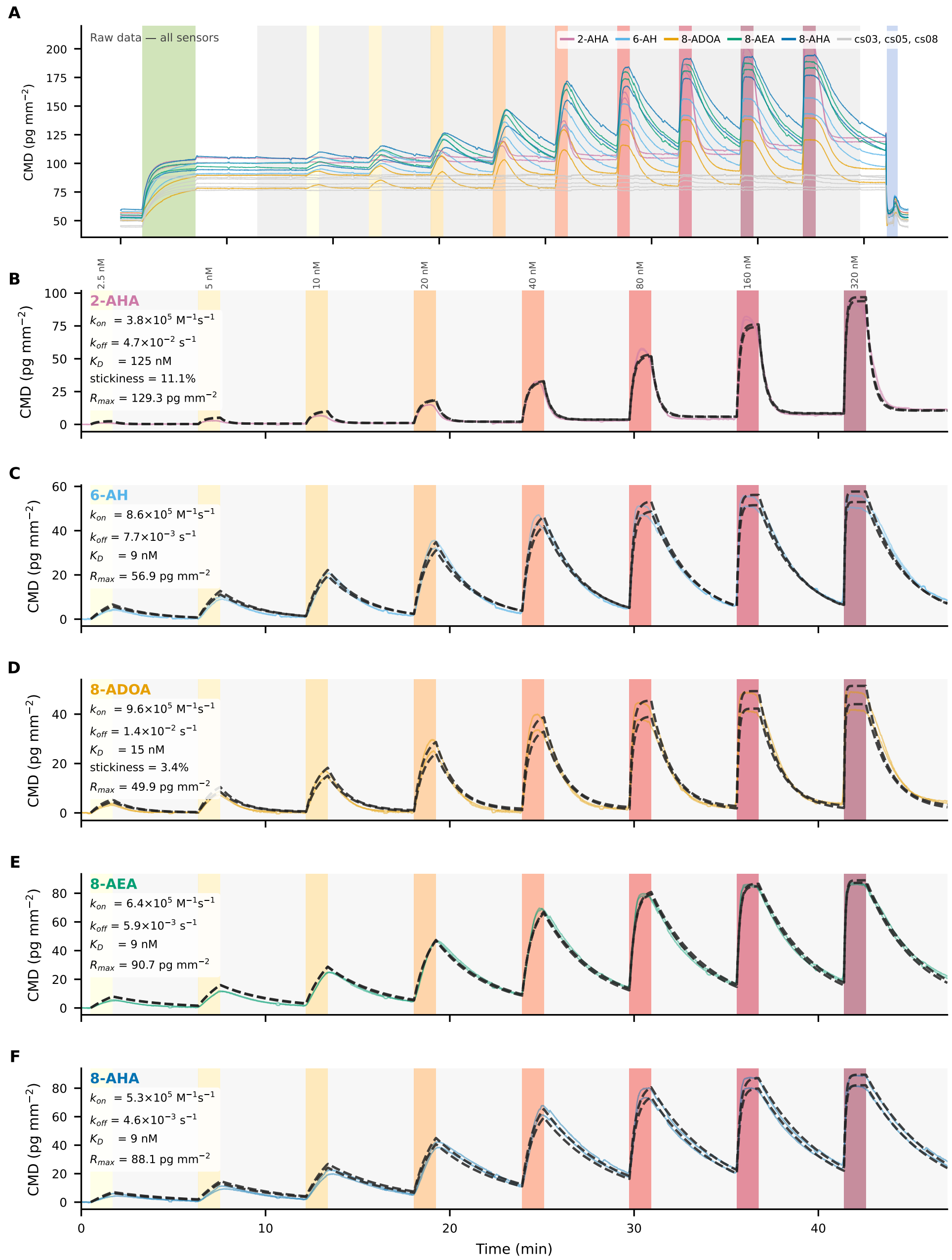

S7 Fig (t) DDI-SCK overview at 25 °C (SCK 3 cycles-1 25deg, SCK cycle 3). (A) Raw CMD sensorgrams for all 16 sensors (immobilization, SCK cycle, regeneration). Traces color-coded by compound; gray = control spots (cs03, cs05, cs08). Shading: olive = immobilization, blue = regeneration wash, warm scale = SCK association (low to high concentration), light gray = dissociation. **(B–F)** Per-sensor kinetic traces and Langmuir 1:1 fits (dashed black) at 25 °C; k_{on}, k_{off}, K_D, and $R_{\max}$ annotated in each panel.

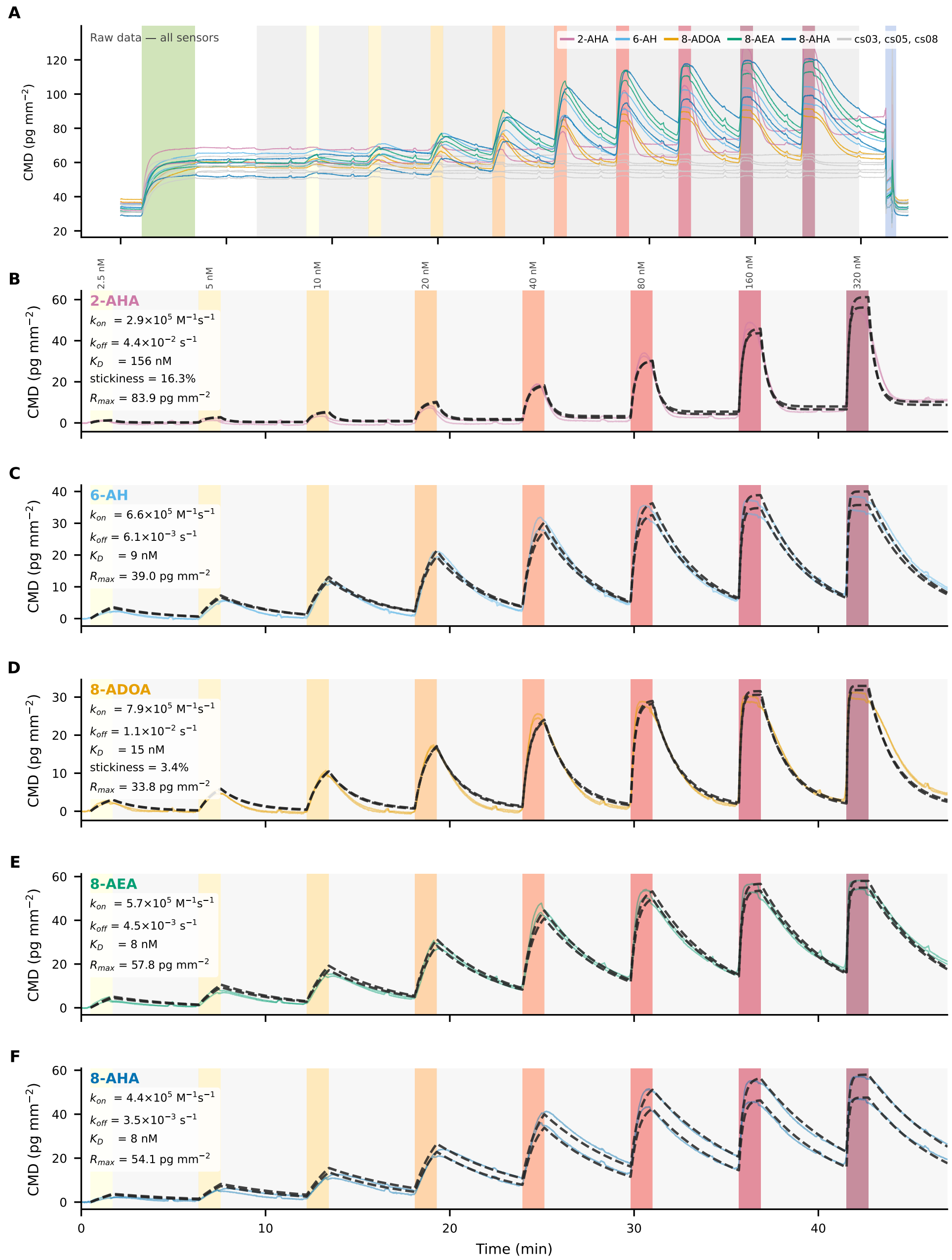

S7 Fig (u) DDI-SCK overview at 25 °C (SCK 3 cycles other way 25 deg, SCK cycle 1). (A) Raw CMD sensorgrams for all 16 sensors (immobilization, SCK cycle, regeneration). Traces color-coded by compound; gray = control spots (cs03, cs05, cs08). Shading: olive = immobilization, blue = regeneration wash, warm scale = SCK association (low to high concentration), light gray = dissociation. **(B–F)** Per-sensor kinetic traces and Langmuir 1:1 fits (dashed black) at 25 °C; k_{on}, k_{off}, K_D, and R_{max} annotated in each panel.

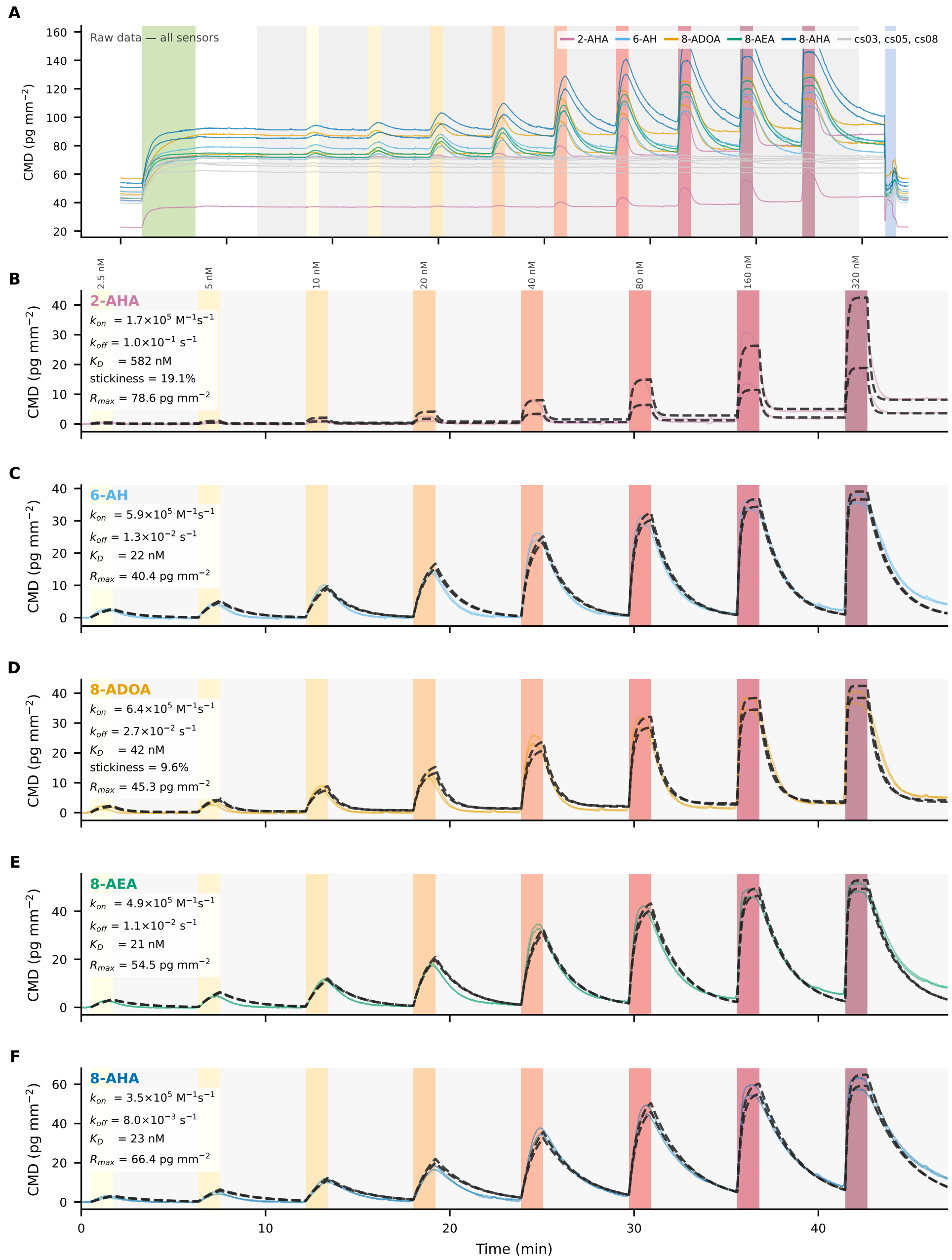

S7 Fig (v) DDI-SCK overview at 30 °C (SCK 3 combined 25 15 30 deg-1, SCK cycle 2). (A) Raw CMD sensorgrams for all 16 sensors (immobilization, SCK cycle, regeneration). Traces color-coded by compound; gray = control spots (cs03, cs05, cs08). Shading: olive = immobilization, blue = regeneration wash, warm scale = SCK association (low to high concentration), light gray = dissociation. **(B–F)** Per-sensor kinetic traces and Langmuir 1:1 fits (dashed black) at 30 °C; k_{on}, k_{off}, K_D, and $R_{\max}$ annotated in each panel.

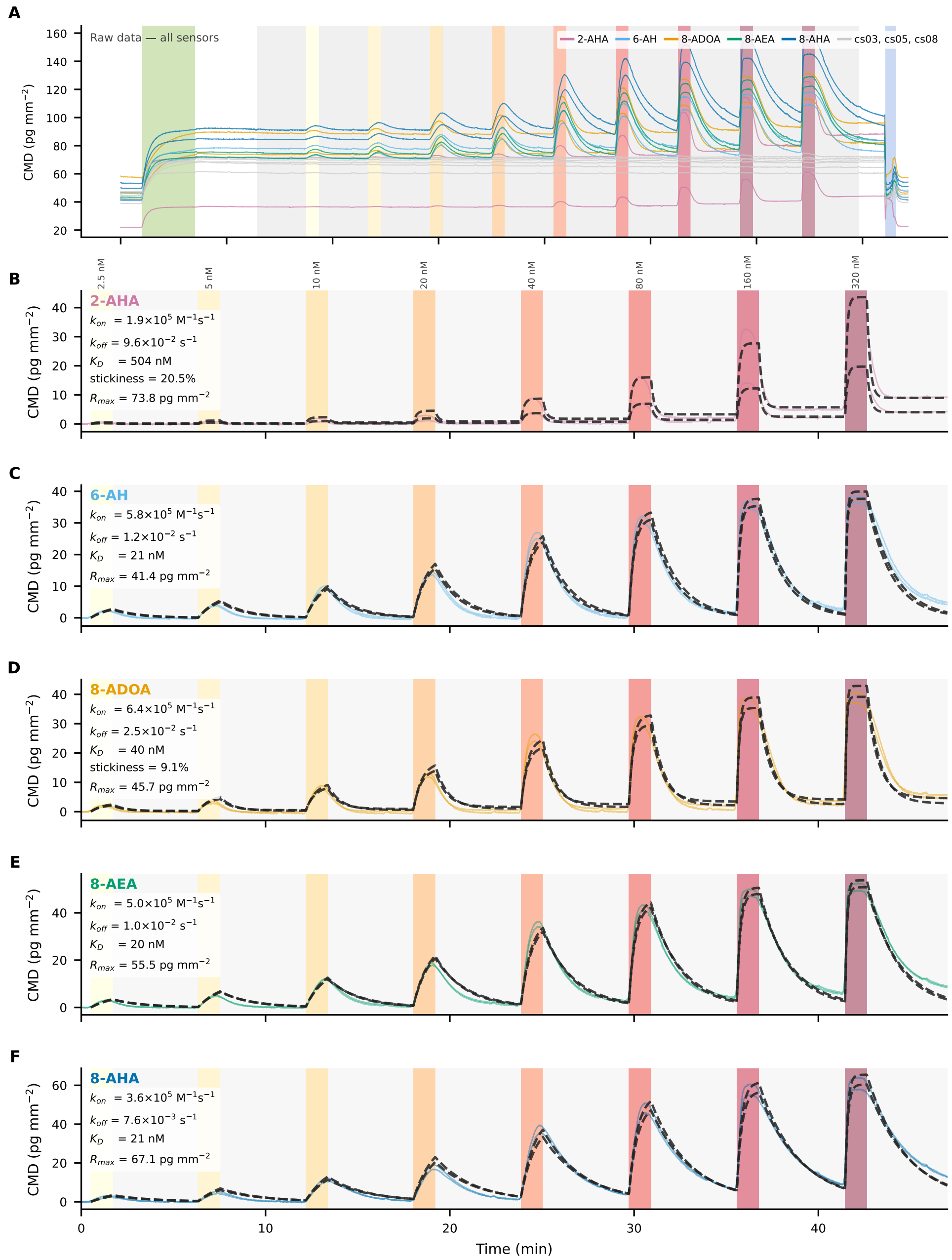

S7 Fig (w) DDI-SCK overview at 30 °C (SCK 3 combined 25 15 30 deg-1, SCK cycle 1). (A) Raw CMD sensorgrams for all 16 sensors (immobilization, SCK cycle, regeneration). Traces color-coded by compound; gray = control spots (cs03, cs05, cs08). Shading: olive = immobilization, blue = regeneration wash, warm scale = SCK association (low to high concentration), light gray = dissociation. **(B–F)** Per-sensor kinetic traces and Langmuir 1:1 fits (dashed black) at 30 °C; k_{on}, k_{off}, K_D, and $R_{\max}$ annotated in each panel.

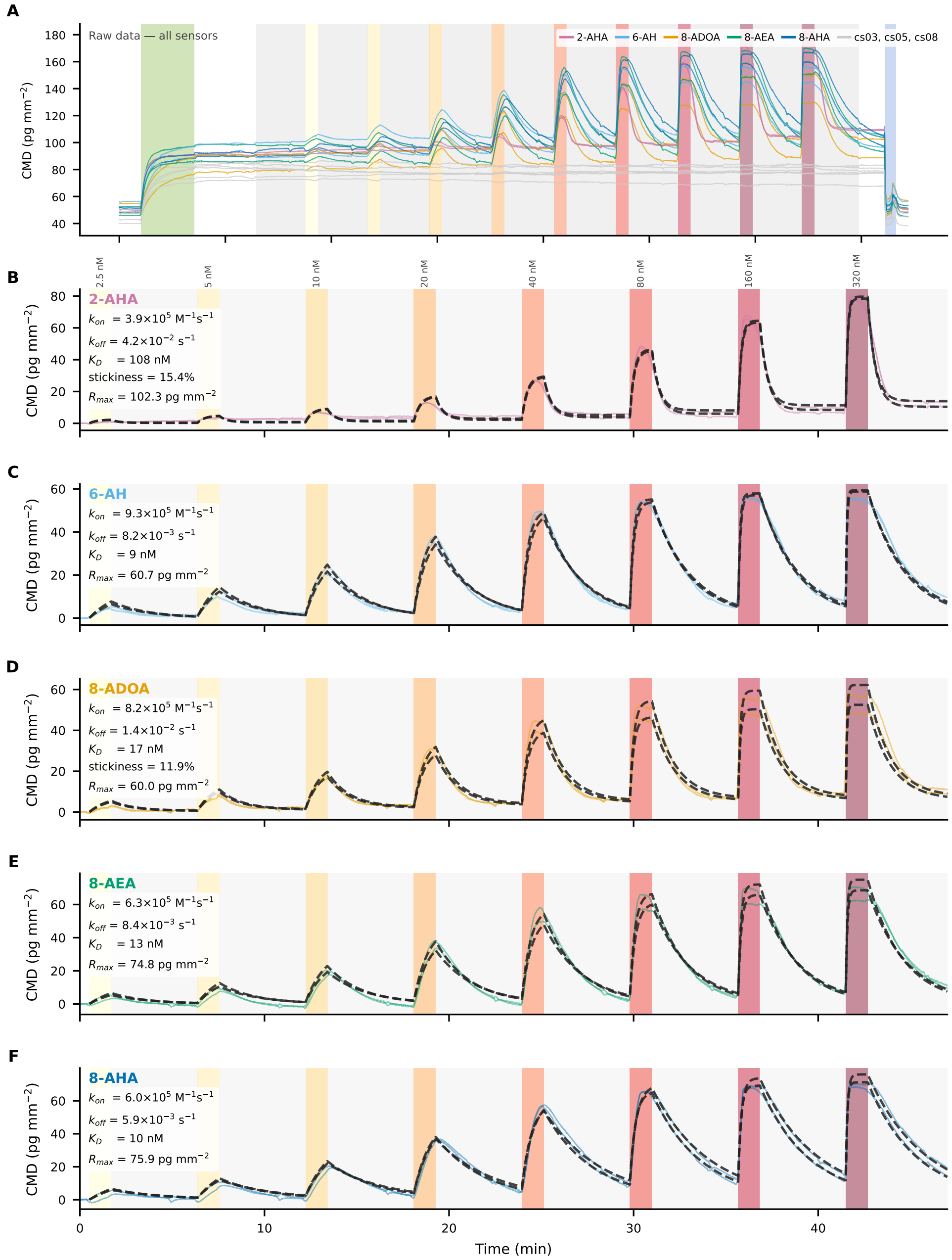

S7 Fig (x) DDI-SCK overview at 30 °C (SCK 3 cycles-2 30deg, SCK cycle 1). (A) Raw CMD sensorgrams for all 16 sensors (immobilization, SCK cycle, regeneration). Traces color-coded by compound; gray = control spots (cs03, cs05, cs08). Shading: olive = immobilization, blue = regeneration wash, warm scale = SCK association (low to high concentration), light gray = dissociation. **(B–F)** Per-sensor kinetic traces and Langmuir 1:1 fits (dashed black) at 30 °C; k_{on}, k_{off}, K_D, and R_{max} annotated in each panel.

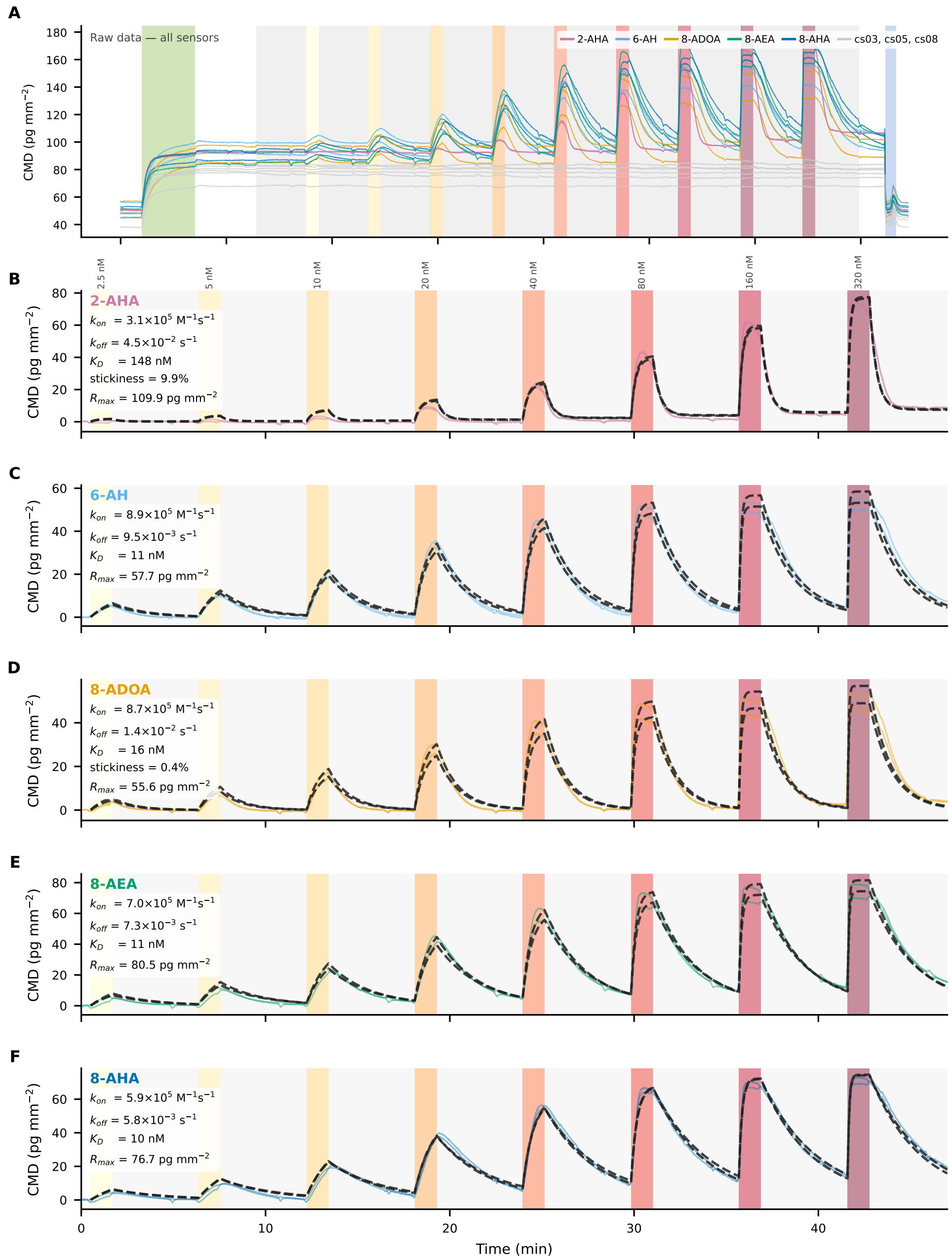

S7 Fig (y) DDI-SCK overview at 30 °C (SCK 3 cycles-2 30deg, SCK cycle 2). (A) Raw CMD sensorgrams for all 16 sensors (immobilization, SCK cycle, regeneration). Traces color-coded by compound; gray = control spots (cs03, cs05, cs08). Shading: olive = immobilization, blue = regeneration wash, warm scale = SCK association (low to high concentration), light gray = dissociation. **(B–F)** Per-sensor kinetic traces and Langmuir 1:1 fits (dashed black) at 30 °C; k_{on}, k_{off}, K_D, and $R_{\max}$ annotated in each panel.

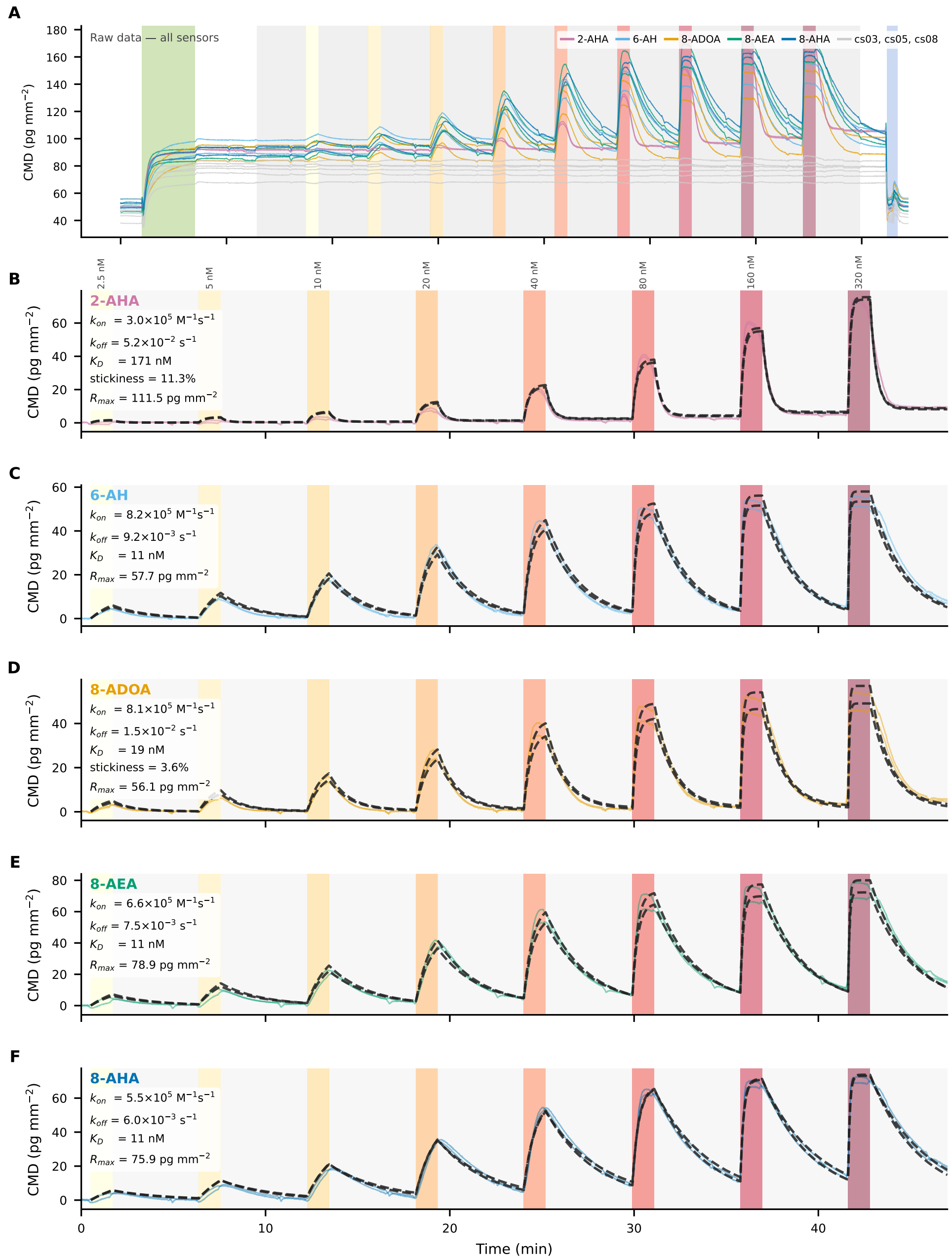

S7 Fig (z) DDI-SCK overview at 30 °C (SCK 3 cycles-2 30deg, SCK cycle 3). (A) Raw CMD sensorgrams for all 16 sensors (immobilization, SCK cycle, regeneration). Traces color-coded by compound; gray = control spots (cs03, cs05, cs08). Shading: olive = immobilization, blue = regeneration wash, warm scale = SCK association (low to high concentration), light gray = dissociation. **(B–F)** Per-sensor kinetic traces and Langmuir 1:1 fits (dashed black) at 30 °C; $k_{\rm on}$, $k_{\rm off}$, K_D, and $R_{\rm max}$ annotated in each panel.

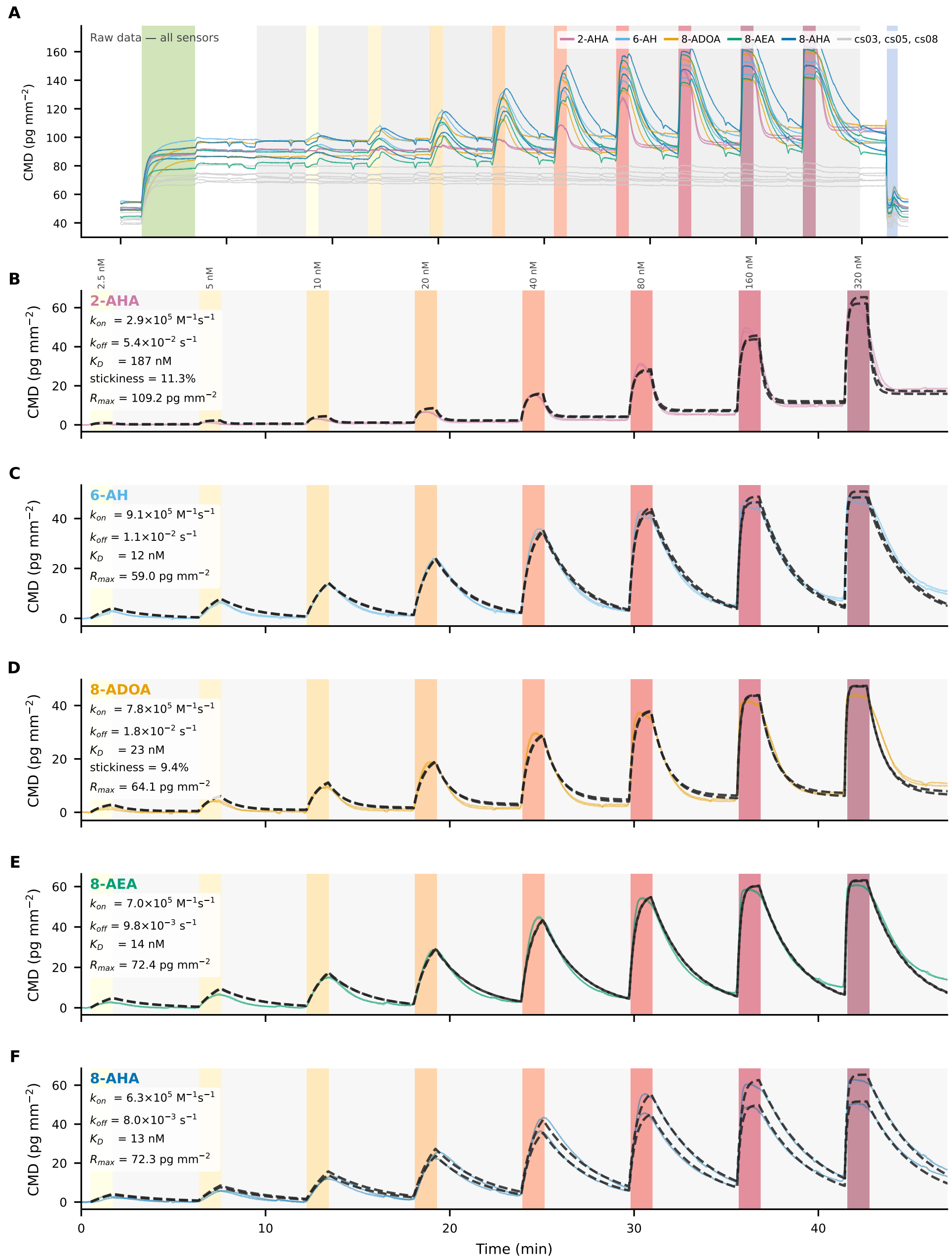

S7 Fig (aa) DDI-SCK overview at 35 °C (SCK Copy -2, SCK cycle 1). **(A)** Raw CMD sensorgrams for all 16 sensors (immobilization, SCK cycle, regeneration). Traces color-coded by compound; gray = control spots (cs03, cs05, cs08). Shading: olive = immobilization, blue = regeneration wash, warm scale = SCK association (low to high concentration), light gray = dissociation. **(B–F)** Per-sensor kinetic traces and Langmuir 1:1 fits (dashed black) at 35 °C; k_{on}, k_{off}, K_D, and $R_{\max}$ annotated in each panel.

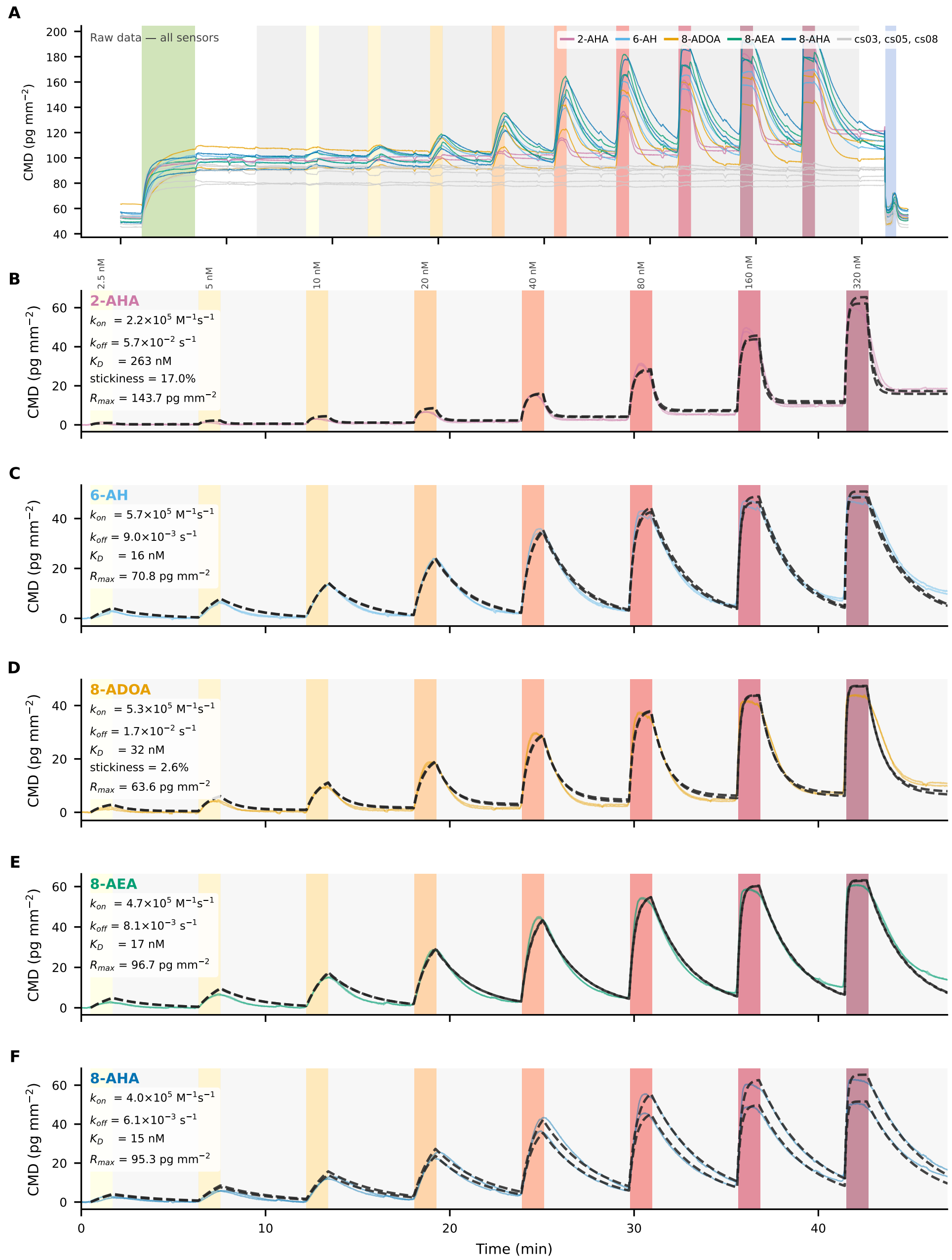

S7 Fig (ab) DDI-SCK overview at 35 °C (SCK-1 35 deg, SCK cycle 1). **(A)** Raw CMD sensorgrams for all 16 sensors (immobilization, SCK cycle, regeneration). Traces color-coded by compound; gray = control spots (cs03, cs05, cs08). Shading: olive = immobilization, blue = regeneration wash, warm scale = SCK association (low to high concentration), light gray = dissociation. **(B–F)** Per-sensor kinetic traces and Langmuir 1:1 fits (dashed black) at 35 °C; k_{on}, k_{off}, K_D, and $R_{\max}$ annotated in each panel.

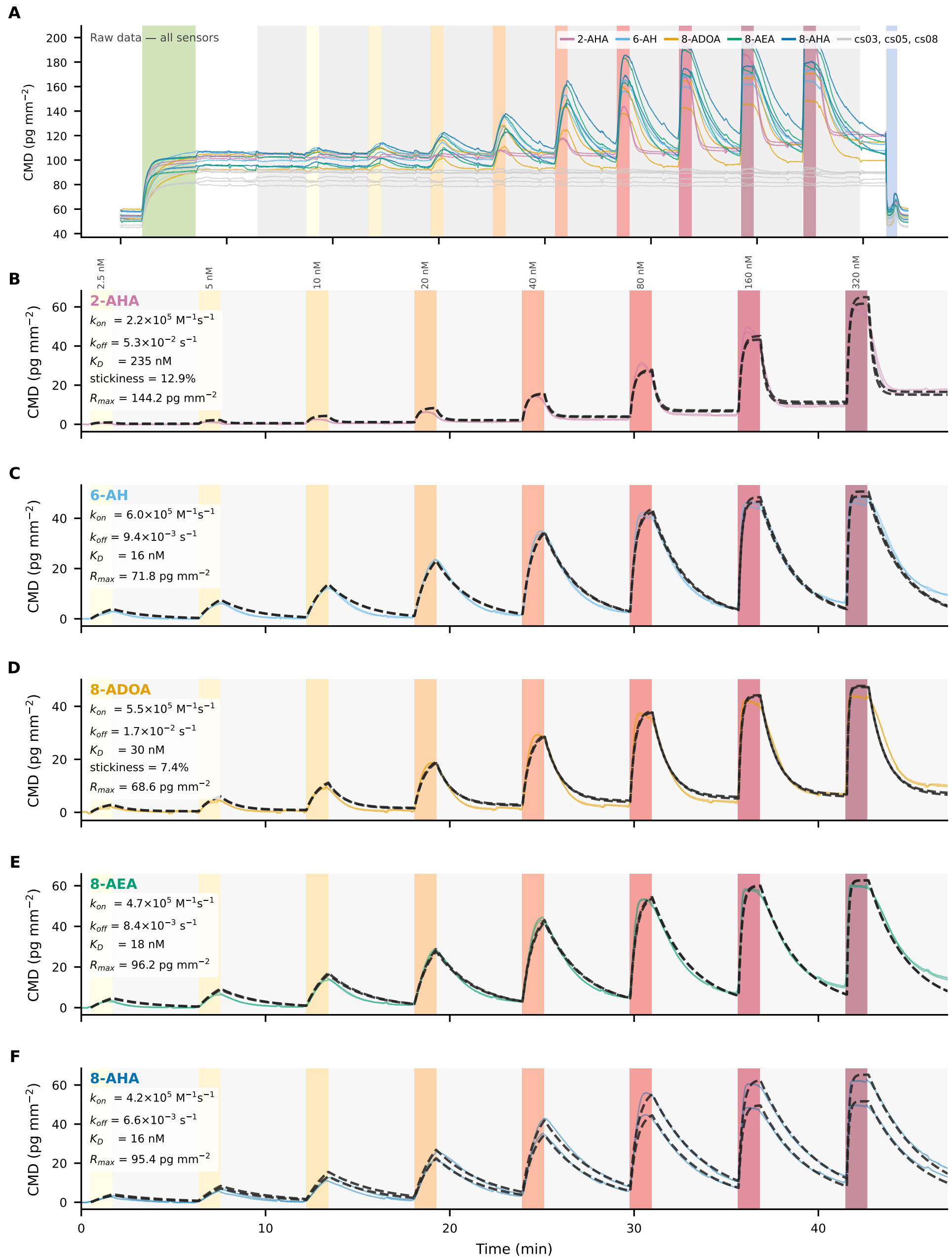

S7 Fig (ac) DDI-SCK overview at 35 °C (SCK-1 35 deg, SCK cycle 2). **(A)** Raw CMD sensorgrams for all 16 sensors (immobilization, SCK cycle, regeneration). Traces color-coded by compound; gray = control spots (cs03, cs05, cs08). Shading: olive = immobilization, blue = regeneration wash, warm scale = SCK association (low to high concentration), light gray = dissociation. **(B–F)** Per-sensor kinetic traces and Langmuir 1:1 fits (dashed black) at 35 °C; k_{on}, k_{off}, K_D, and $R_{\max}$ annotated in each panel.

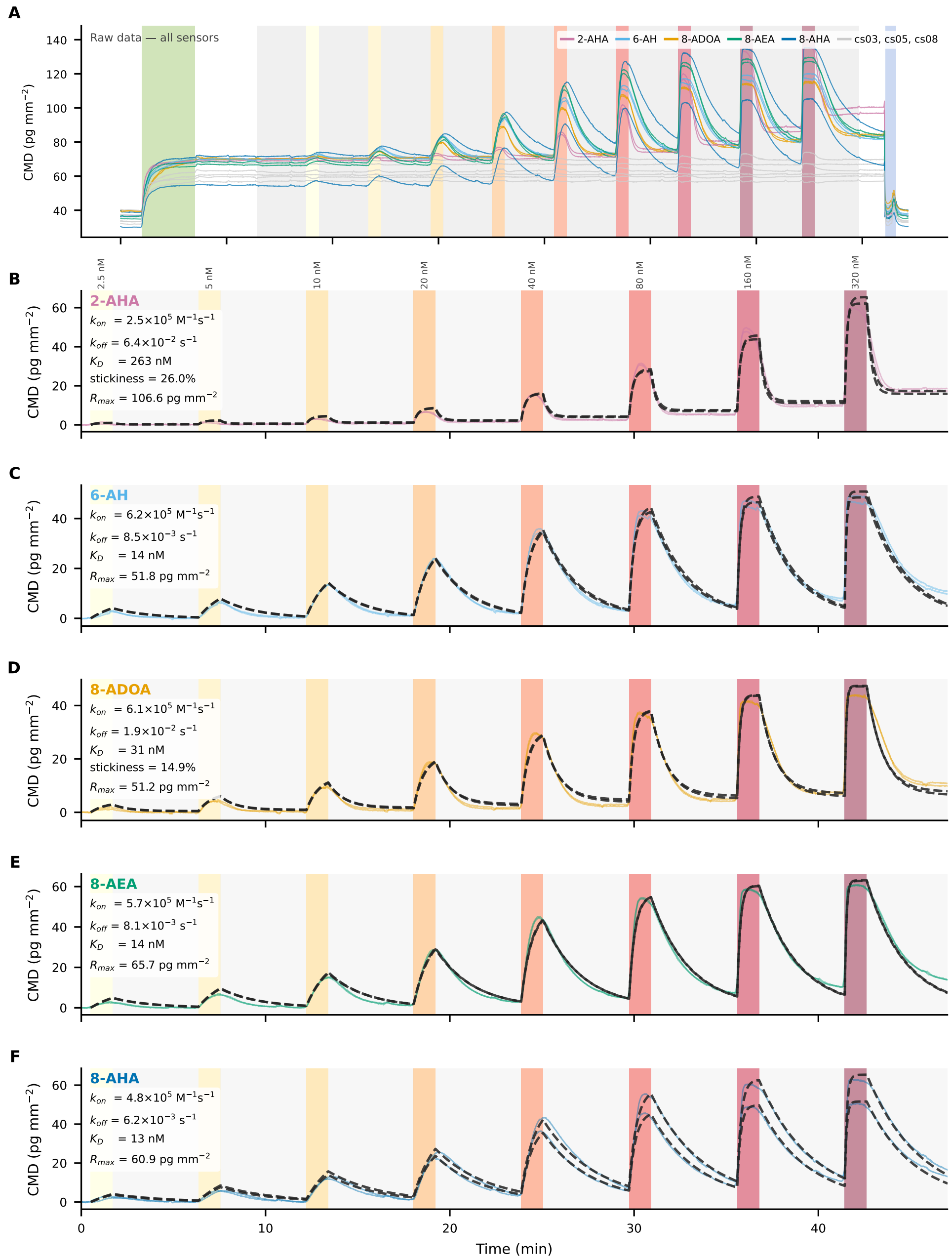

S7 Fig (ad) DDI-SCK overview at 35 °C (SCK 3 cycles 35deg 1, SCK cycle 1). (A) Raw CMD sensorgrams for all 16 sensors (immobilization, SCK cycle, regeneration). Traces color-coded by compound; gray = control spots (cs03, cs05, cs08). Shading: olive = immobilization, blue = regeneration wash, warm scale = SCK association (low to high concentration), light gray = dissociation. **(B–F)** Per-sensor kinetic traces and Langmuir 1:1 fits (dashed black) at 35 °C; k_{on}, k_{off}, K_D, and $R_{\max}$ annotated in each panel.

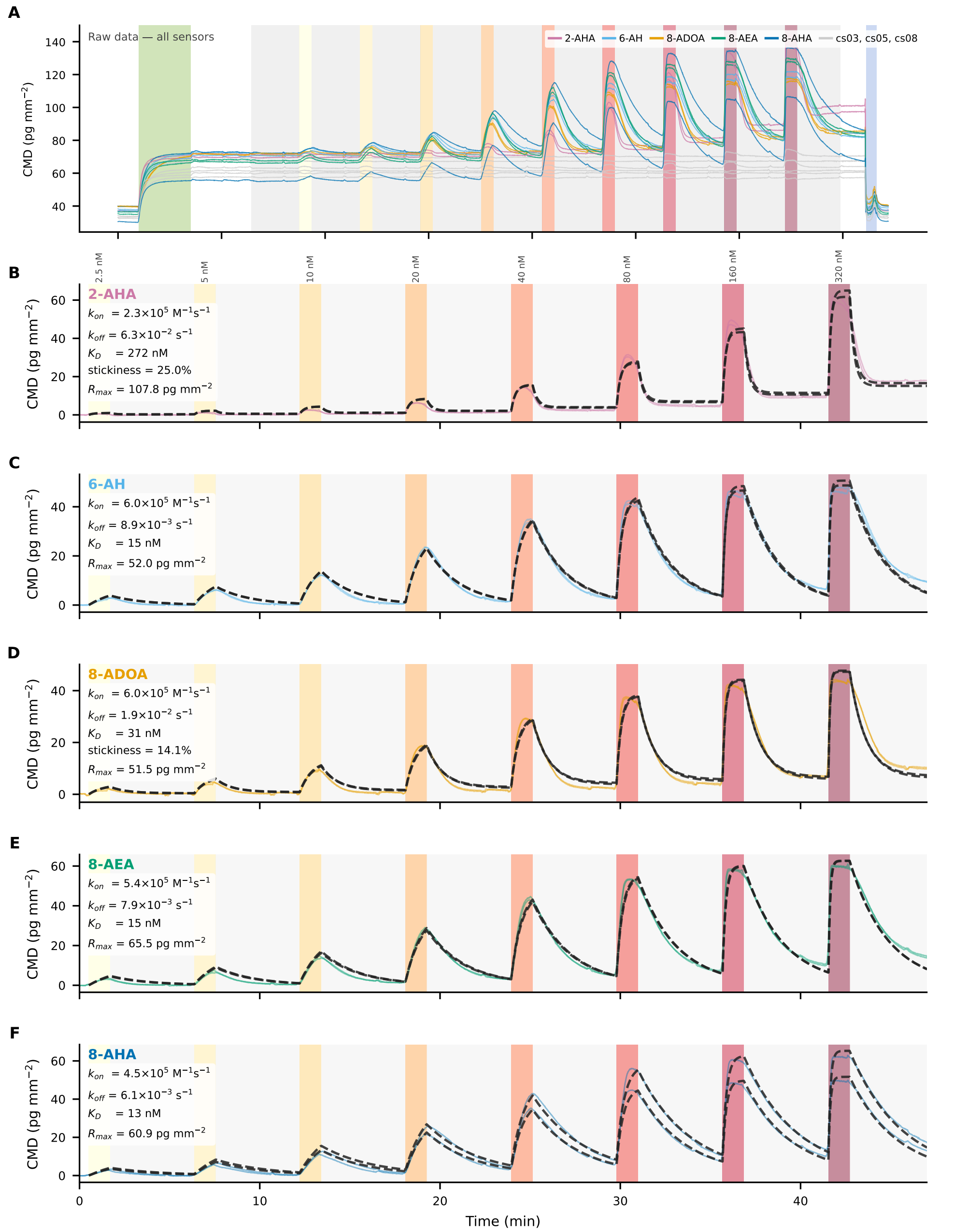

S7 Fig (ae) DDI-SCK overview at 35 °C (SCK 3 cycles 35deg 1, SCK cycle 2). (A) Raw CMD sensorgrams for all 16 sensors (immobilization, SCK cycle, regeneration). Traces color-coded by compound; gray = control spots (cs03, cs05, cs08). Shading: olive = immobilization, blue = regeneration wash, warm scale = SCK association (low to high concentration), light gray = dissociation. **(B–F)** Per-sensor kinetic traces and Langmuir 1:1 fits (dashed black) at 35 °C; k_{on}, k_{off}, K_D, and $R_{\max}$ annotated in each panel.

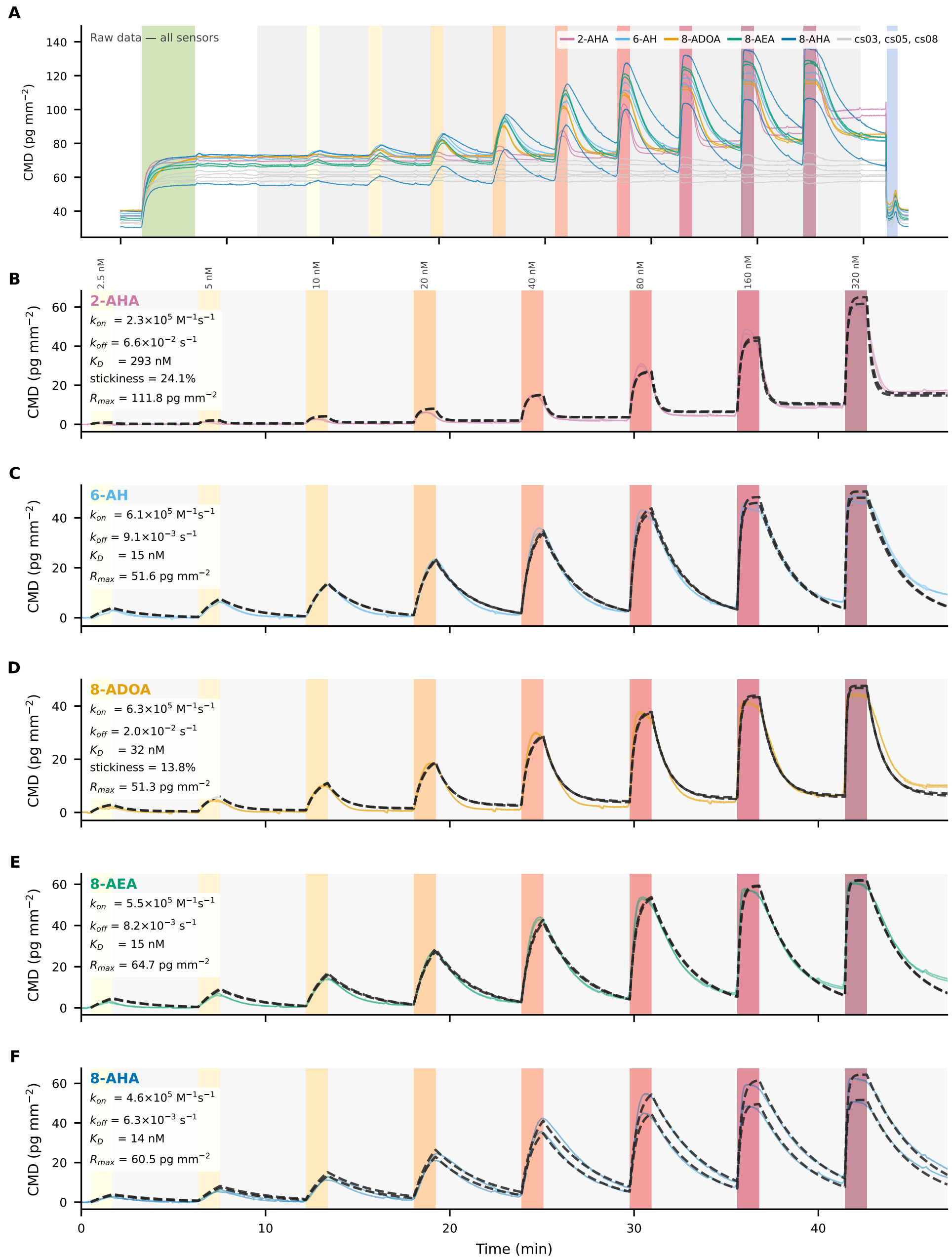

S7 Fig (af) DDI-SCK overview at 35 °C (SCK 3 cycles 35deg 1, SCK cycle 3). (A) Raw CMD sensorgrams for all 16 sensors (immobilization, SCK cycle, regeneration). Traces color-coded by compound; gray = control spots (cs03, cs05, cs08). Shading: olive = immobilization, blue = regeneration wash, warm scale = SCK association (low to high concentration), light gray = dissociation. **(B–F)** Per-sensor kinetic traces and Langmuir 1:1 fits (dashed black) at 35 °C; k_{on}, k_{off}, K_D, and $R_{\max}$ annotated in each panel.

S4 Table. DDI immobilization levels (CMD signal increase after cAMP–oligonucleotide capture). Mean ± SD of the coherent mass density increase (ΔCMD, pg mm^{-2}) measured at the end of the equilibration period following DNA-directed immobilization, relative to the pre-immobilization baseline. Values are reported per

compound and temperature across all DDI-SCK cycles and runs.

Compound	15 °C (n)	20 °C (n)	25 °C (n)	30 °C (n)	35 °C (n)
6-AH-cAMP	37.2 ± 4.6 (6)	31.1 ± 5.2 (12)	24.7 ± 19.1 (12)	41.4 ± 2.4 (6)	39.1 ± 5.9 (12)
8-AHA-cAMP	41.1 ± 2.2 (6)	34.5 ± 8.3 (12)	25.8 ± 20.6 (12)	40.3 ± 1.4 (6)	35.9 ± 8.4 (12)
2-AHA-cAMP	44.3 ± 2.9 (6)	37.1 ± 7.1 (12)	28.4 ± 22.5 (12)	41.6 ± 0.6 (6)	38.8 ± 6.6 (12)
8-AEA-cAMP	40.8 ± 2.9 (6)	36.1 ± 8.8 (12)	26.8 ± 21.5 (12)	41.0 ± 2.6 (6)	37.6 ± 7.2 (12)
8-ADOA-cAMP	20.8 ± 5.3 (6)	19.9 ± 3.6 (12)	18.9 ± 14.8 (12)	36.2 ± 3.4 (6)	36.6 ± 5.8 (12)

All values in pg mm^{-2}. n = number of individual immobilization-level measurements: two positive mologram per compound for each immobilization cycle included in this summary. These counts summarize immobilization levels before kinetic-fit quality exclusions and therefore are not identical to the retained DDI-SCK kinetic-fit counts reported in the main text. The elevated standard deviations at 25 °C reflect contributions from a combined multi-temperature experiment in which immobilization levels were lower. 8-ADOA-cAMP shows consistently lower immobilization levels than the other four derivatives, possibly reflecting differences in conjugation efficiency or hybridization kinetics.

S8. Trypsin-EDTA surface regeneration

Between the 50% human serum and buffer A MCK titrations at 20 °C on the covalently immobilized chip, the surface was regenerated by enzymatic digestion with trypsin to remove non-specifically adsorbed serum material. The regeneration protocol consisted of: (i) a short pulse of 3 M GuHCl / 125 mM NaOH (0.5 min, 400 µL/min) to denature residual analyte; (ii) a buffer wash (3 min, 300 µL/min); (iii) a 50-minute injection of 1× Trypsin-EDTA solution (10 µL/min); (iv) a buffer wash (10 min, 300 µL/min); (v) a second GuHCl/NaOH pulse; and (vi) a final buffer rinse. The trypsin solution was prepared by diluting StableCell™ Trypsin Solution 5× (Sigma-Aldrich, cat. no. T2605, lot 0000308412; 2.5 g porcine trypsin and 0.2 g EDTA·4Na per liter of Hanks' Balanced Salt Solution) to 1× working concentration in PBS-T (0.05% Tween-20). A representative regeneration sensorgram is shown in S8 Fig.

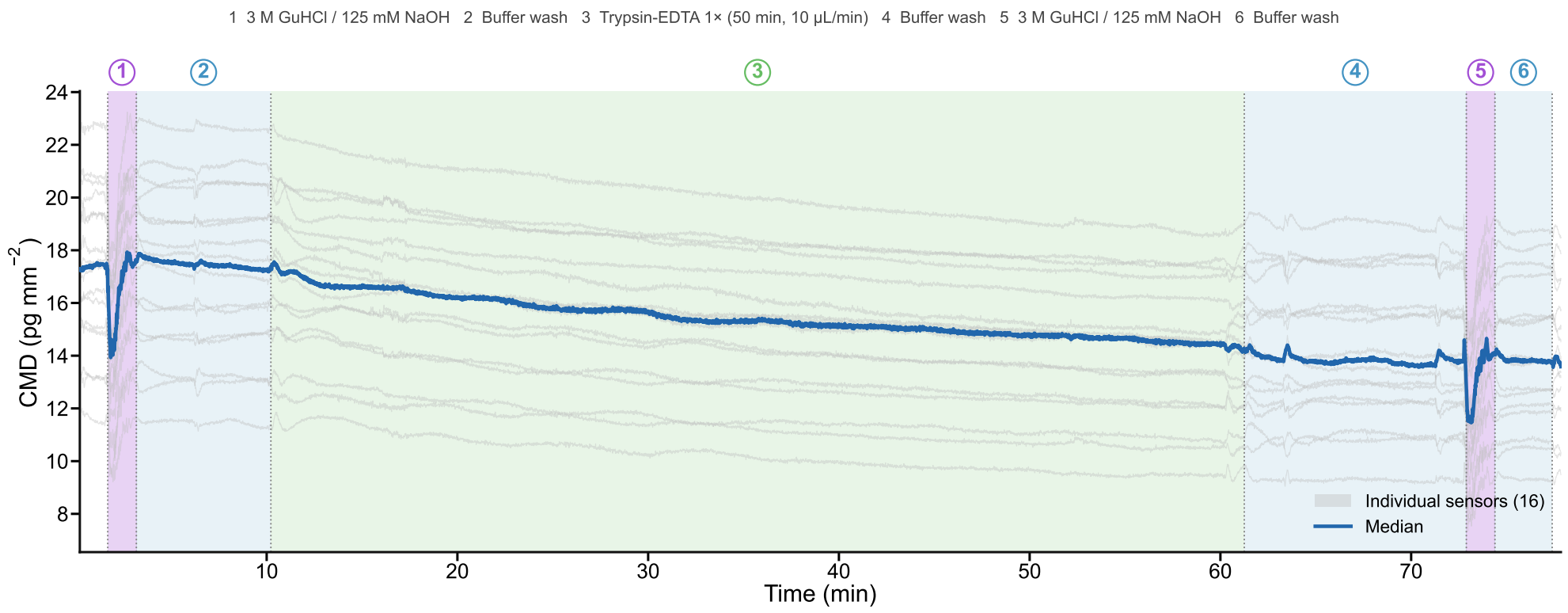

S8 Fig. Representative trypsin-EDTA regeneration sensorgram. Coherent mass density (CMD) traces recorded simultaneously from 16 sensor spots during a single regeneration cycle between the serum and buffer MCK titrations at 20 °C. Phase 1: 3 M GuHCl / 125 mM NaOH pulse (0.5 min, 400 µL/min); Phase 2: buffer wash (3 min, 300 µL/min); Phase 3: Trypsin-EDTA 1× digestion (50 min, 10 µL/min); Phase 4: buffer wash (10 min, 300 µL/min); Phase 5: second GuHCl/NaOH pulse; Phase 6: final buffer rinse. Individual sensor traces are shown in gray; the blue line indicates the median across all 16 sensors.

References

1. Moll D, Schweinsberg S, Hammann C, Herberg FW. Comparative thermodynamic analysis of cyclic nucleotide binding to protein kinase A. Biological Chemistry. 2007;388(2). doi:10.1515/BC.2007.018.

2. Frutiger A, Tanno A, Hwu S, Tiefenauer RF, Vörös J, Nakatsuka N. Nonspecific Binding—Fundamental Concepts and Consequences for Biosensing Applications. Chemical Reviews. 2021;121(13):8095–8160. doi:10.1021/acs.chemrev.1c00044.

3. Gatterdam V, Frutiger A, Stengele KP, Heindl D, Lübbers T, Vörös J, et al. Focal molography is a new method for the in situ analysis of molecular interactions in biological samples. Nature Nanotechnology. 2017;12(11):1089–1095. doi:10.1038/nnano.2017.168.

4. Frutiger A, Blickenstorfer Y, Bischof S, Forró C, Lauer M, Gatterdam V, et al. Principles for Sensitive and Robust Biomolecular Interaction Analysis: The Limits of Detection and Resolution of Diffraction-Limited Focal Molography. Physical Review Applied. 2019;11(1):014056. doi:10.1103/PhysRevApplied.11.014056.

5. Frutiger A, Tschannen CD, Blickenstorfer Y, Reichmuth AM, Fattinger C, Vörös J. Image reversal reactive immersion lithography improves the detection limit of focal molography. Optics Letters. 2018;43(23):5801. doi:10.1364/OL.43.005801.
