# Supplementary material for: High-throughput thermodynamic fingerprinting of protein–ligand interactions by DNA-directed focal molography
Source: PLoS One. 2026 Oct 22;21(10):e0354008. doi: 10.1371/journal.pone.0354008 (PMC13690234; doi:10.1371/journal.pone.0354008)
Supplement: Supplementary file 2 [file pone.0354008.s003.pdf]

Response to Reviewers

Manuscript PONE-D-26-32924: High-throughput thermodynamic fingerprinting of protein–ligand interactions by DNA-directed focal molography

Oehninger, Notova, and Frutiger

August 26, 2026

Dear Dr. Paci and Reviewer,

We thank the Academic Editor and Reviewer #1 for their careful and constructive evaluation of our manuscript, and for recognizing the work as a "technically sound and potentially valuable methodological contribution." We have revised the manuscript to address every point raised. In brief, we have:

1. **Reframed all extracted thermodynamic quantities as apparent, format-specific, qualitative descriptors** of the focal molography measurement—rather than intrinsic properties of the molecular interaction—consistently across the abstract, Results, Methods, table/figure captions, and Discussion (Reviewer #1's principal point).
2. **Expanded the limitations discussion** to treat explicitly the effects of surface immobilization, DNA-directed ligand presentation, and the non-dissociating-fraction model on the kinetic and thermodynamic parameters.
3. **Clarified the statistical basis of the reported uncertainties**, making explicit that they capture within-assay (technical and assay) variability rather than independent chip-to-chip, protein-preparation, or conjugate-preparation reproducibility.
4. **Revised the Funding statement** to reflect that the funder and the authors' employer are the same organization, replacing the previous blanket "no role" wording.
5. **Reconciled the Data Availability statement** with the submission system, designating the Zenodo repository as the single source of all underlying data.
6. **Addressed all editorial requirements**: the ethics statement now appears only in the Methods, every author is linked to an affiliation (including a current-affiliation entry for J. Oehninger), and the copyright status of Figures S2 and S6 is clarified as original, author-generated content.

Below we address each comment in detail. Reviewer and editor comments are shown in **blue**; our responses are in black. Changes are marked in the "Revised Manuscript with Track Changes" file; unmarked text is quoted here for convenience.

Academic Editor — Journal Requirements

1. Please ensure that your manuscript meets PLOS ONE's style requirements, including those for file naming.

The manuscript has been prepared using the PLOS ONE templates, and all files are named according to the submission guidelines (Manuscript; Revised Manuscript with Track Changes; Response to Reviewers; Supporting Information).

2. Your ethics statement should only appear in the Methods section of your manuscript.

The ethics statement previously appeared as a stand-alone section in the back matter. We have moved it into the Materials and methods section (now a subsection, "Ethics statement," at the end of Methods) and removed it from all other locations; it therefore now appears only within the Methods.

3. We note that the grant information you provided in the 'Funding Information' and 'Financial Disclosure' sections do not match.

This study was funded solely by lino Biotech AG as internal (company) funding; there is no external grant and therefore no associated grant number. We have ensured that the 'Funding Information' and 'Financial Disclosure' fields in the submission system state this consistently, and we have reworded the manuscript Funding section accordingly (see Reviewer #1, point 4).

4. Please amend your list of authors on the manuscript to ensure that each author is linked to an affiliation.

All three authors are now linked to an affiliation. The work was performed at the R & D department of lino Biotech AG (affiliation 1), the affiliation of all authors. As J. Oehninger has since moved institutions, his present affiliation is additionally indicated with a current-affiliation marker: "Current affiliation: Eawag, Swiss Federal Institute of Aquatic Science and Technology, Dübendorf, Switzerland." The affiliation where the work was performed (lino Biotech AG) is retained for all authors.

5. We are unable to open your Supporting Information file [manuscript_plos.tex].

The file in question is the LaTeX source of the main manuscript (`manuscript_plos.tex`), which had been uploaded as the LaTeX Source item without the auxiliary files (`.bib`, `.bst`, `.bbl`) needed to compile it. As the manuscript is reviewed from the compiled PDF at this stage, we have removed the incomplete LaTeX source item to resolve the issue; we will provide the complete, compilable LaTeX source upon acceptance, in line with PLOS guidelines. The Supporting Information file itself (`S1_File.pdf`) was unaffected and opens normally.

6. We note that Figure S2, S6 in your submission contain copyrighted images.

Figures S2 and S6 are original figures prepared by the authors specifically for this manuscript. They consist of photographs and schematics of the instrumentation used in this study, taken and produced by the authors, and have not been published elsewhere; they contain no third-party or previously published copyrighted material. The copyright holder, lino Biotech AG, consents to their publication under the Creative Commons Attribution License (CC BY 4.0).

We note for completeness that the images depict commercial instrumentation bearing the MACS® trademark (a registered trademark of Miltenyi Biotec) and incidental third-party laboratory hardware (e.g. a Thorlabs temperature controller). These appear only as a factual record of the experimental setup; no copyrighted image belonging to those parties is reproduced, and the trademark is used descriptively.

7. If the reviewer comments include a recommendation to cite specific previously published works, please review and evaluate these publications...

Reviewer #1 did not request the citation of any specific works. No citations were added on this basis.

Reviewer #1

This manuscript presents an application of focal molography... to measure temperature-dependent protein–ligand binding kinetics and derive apparent thermodynamic parameters. ... The work is well executed and addresses an interesting technological development. However, I believe the manuscript requires major revision before publication.

We thank the reviewer for this positive and constructive assessment, and for recognizing the advantages of focal molography for measurements under changing temperature and in complex matrices. We have addressed each of the concerns raised, as detailed below.

Major Points

1. The principal weakness is the interpretation of the thermodynamic analysis. While the authors appropriately describe the extracted quantities as "apparent" thermodynamic fingerprints, the manuscript still tends to overstate their physical significance. The comparison with published SPR and ITC data clearly shows that the enthalpy–entropy decomposition depends strongly on the assay format and surface presentation. Consequently, these quantities should be presented as qualitative descriptors within the focal molography platform rather than as intrinsic thermodynamic properties of the molecular interaction.

We fully agree and have revised the manuscript so that the extracted quantities are consistently presented as **apparent, format-specific, qualitative descriptors** of the focal molography measurement, not as intrinsic thermodynamic properties of the cAMP–PKA-R interaction:

- **Results narrative.** We removed the remaining categorical, physical-property phrasings. For example, "all five show exothermic binding... but the balance between enthalpic and entropic contributions differs" now reads "all five show an apparently exothermic binding signature (apparent ΔH°...), but the apparent balance between enthalpic and entropic contributions differs between ligands"; "2-AHA-cAMP is more strongly enthalpy-driven" now reads "2-AHA-cAMP shows a more strongly enthalpy-weighted apparent profile"; and the summary sentence now refers to a "distinct but internally consistent *apparent* thermodynamic profile."
- **Methods.** The van 't Hoff parameters are now introduced as "*Apparent* standard-state thermodynamic parameters," with an explicit statement that the "°" superscript denotes only the 1 M concentration reference and that these are "apparent, format-specific parameters of the surface-based assay rather than solution-phase constants."
- **Captions.** The summary tables are now titled "apparent equilibrium thermodynamic parameters" and "apparent activation parameters."
- **Introduction.** References to the output now read "distinct, apparent thermodynamic fingerprints" / "apparent enthalpic and entropic fingerprints."
- **Discussion.** We retained and reinforced the statements that the parameters "are apparent, format-specific quantities, most powerful for ranking and contrasting related ligands rather than as substitutes for calorimetric reference values," that they are "apparent properties of the DNA-tethered, surface-confined presentation rather than of the free-solution interaction," and that "the absolute enthalpy–entropy split is the least transferable part of the comparison." The cross-method (SPR/ITC) comparison is explicitly framed as "external context rather than... a test of thermodynamic equivalence."

Together these changes ensure the thermodynamic quantities are consistently framed as qualitative, platform-specific descriptors for comparative ranking, as the reviewer recommends.

2. A second concern is the limited statistical independence of the data. Although a large number of kinetic fits are reported, these arise predominantly from technical replicates rather than independent chip preparations, ligand conjugations, or protein batches. The uncertainty associated with the reported thermodynamic parameters is therefore likely underestimated.

We agree, and we note that the manuscript already makes this limitation explicit; we have ensured it is unambiguous. In the Methods ("Thermodynamic parameter uncertainties"), we state that the bootstrap standard deviations "capture within-assay variability among retained molograms, cycles, and experimental sessions, but not independent chip-to-chip, protein-preparation, or conjugate-preparation variability." In the Discussion ("Interpretation boundaries and measurement limitations"), we state that the measurements underlying each analysis ($N = 64$ per compound) "are technical and assay replicates... rather than fully independent biochemical replicates, so the bootstrap uncertainties mainly capture within-assay measurement and surface variability, not between-preparation reproducibility." We therefore explicitly caution that the reported uncertainties reflect within-assay precision, and we treat the parameters as comparative rather than absolute throughout.

3. The manuscript would also benefit from a clearer discussion of the limitations imposed by surface immobilization, DNA-directed ligand presentation, and the use of a non-dissociating fraction model for some ligands. These factors may influence both the kinetic and thermodynamic parameters and deserve more explicit consideration.

We have substantially expanded the limitations discussion to treat each of these three factors explicitly:

- **Surface immobilization (new).** Immobilization itself—independently of the coupling chemistry—removes the ligand's translational and rotational freedom relative to the ITC solution reference, confines the interaction to a two-dimensional, locally crowded layer that can promote rebinding and thereby slow the apparent dissociation, and embeds it in the hydrated PEG-brush environment rather than in bulk solvent; each of these effects can shift the measured rate constants and, through them, the enthalpic and (especially) entropic terms, and is a generic reason why surface-based and solution-based decompositions need not agree.
- **DNA-directed ligand presentation (expanded).** The DNA tether sets the ligand's distance from the surface and carries a dense negative charge that modifies the local electrostatics, hydration, and accessible orientational ensemble, while spot-to-spot variation in hybridization density adds a further source of heterogeneity—so the reported parameters are apparent properties of the DNA-tethered, surface-confined presentation.
- **Non-dissociating fraction model (expanded).** The Langmuir 1:1 NonDissRelative model represents the sensorgram as a dissociating Langmuir population superposed on an apparently static, non-releasing fraction; such a fraction is itself a signature of surface heterogeneity (e.g. a slow-off subpopulation, avidity/rebinding-trapped complexes, or partial irreversible capture); the fitted parameters describe the dissociating subpopulation only; and a temperature-dependent non-dissociating fraction could bias the apparent van 't Hoff and Eyring temperature dependence for 8-ADOA-cAMP and 2-AHA-cAMP specifically, making their parameters the most model-dependent in the panel.

4. There is also a disclosure/consistency issue. The competing interest is properly declared... But the statement that the funder had "no role" is awkward because the funder and the authors' employer are effectively the same organization. The data availability statement also appears inconsistent: the submission form mentions a Zenodo DOI, whereas the manuscript text says data are provided as Supporting Information.

We agree on both counts and have corrected them.

- **Funding statement.** We removed the blanket "no role" wording. The Funding section now acknowledges that the funder and the authors' employer are the same organization, and states that "the funder was necessarily involved in the study through the authors themselves—in study design, data collection and analysis, the decision to publish, and preparation of the manuscript," while clarifying that "no external sponsor or third party had any role in the study." It cross-references the Author contributions and Conflict of Interest sections.
- **Data availability.** We reconciled the statement with the submission form. The manuscript now states that all data underlying the findings—raw measurement exports, processed data tables, fitted-trace summaries, and the analysis and figure-generation scripts—are openly available in the Zenodo repository (DOI: 10.5281/zenodo.21103143), "consistent with the Data Availability entry in the submission system," and that the Supporting Information files "provide supplementary methods, figures, and tables only and do not contain additional underlying data."

Summary of Changes

1. **Apparent-parameter framing**: thermodynamic and activation quantities are consistently presented as apparent, format-specific, qualitative descriptors throughout the manuscript.
2. **Expanded limitations**: explicit treatment of surface immobilization, DNA-directed ligand presentation, and the non-dissociating-fraction model.
3. **Statistical basis clarified**: reported uncertainties are stated to capture within-assay, not between-preparation, variability.
4. **Funding statement** revised to reflect that the funder and the authors' employer are the same organization.
5. **Data availability** reconciled with the submission system (Zenodo as the single source of underlying data).
6. **Editorial requirements**: ethics statement moved into Methods; all authors linked to affiliations (with a current-affiliation entry for J. Oehninger); Figures S2/S6 copyright status clarified as original author-generated content.

We believe these revisions fully address the comments raised and have improved the manuscript, particularly by ensuring the thermodynamic quantities are consistently and appropriately framed as apparent, comparative descriptors. We thank the reviewer and editor again for their time and constructive input.

Sincerely,
John Oehninger, Simona Notova, and Andreas Frutiger
