# Supplementary material for: High-throughput thermodynamic fingerprinting of protein–ligand interactions by DNA-directed focal molography
Source: PLoS One. 2026 Oct 22;21(10):e0354008. doi: 10.1371/journal.pone.0354008 (PMC13690234; doi:10.1371/journal.pone.0354008)
Supplement: Peer review history

# Peer review: Decision Letter 0

**DOI:** 10.1371/journal.pone.0354008.r001

**Authors:** Emanuele Paci

24 Aug 2026

Dear Dr. Frutiger,

Thank you for submitting your manuscript to PLOS One. After careful consideration, we feel that it has merit but does not fully meet PLOS One’s publication criteria as it currently stands. Therefore, we invite you to submit a revised version of the manuscript that addresses the points raised during the review process.

Please submit your revised manuscript by Oct 23 2026 11:59PM. If you will need more time than this to complete your revisions, please reply to this message or contact the journal office at plosone@plos.org. When you're ready to submit your revision, log on to https://www.editorialmanager.com/pone/ and select the 'Submissions Needing Revision' folder to locate your manuscript file.

- A letter that responds to each point raised by the academic editor and reviewer(s). You should upload this letter as a separate file labeled 'Response to Reviewers'.
- A marked-up copy of your manuscript that highlights changes made to the original version. You should upload this as a separate file labeled 'Revised Manuscript with Track Changes'.
- An unmarked version of your revised paper without tracked changes. You should upload this as a separate file labeled 'Manuscript'.

If you would like to make changes to your financial disclosure, please include your updated statement in your cover letter. Guidelines for resubmitting your figure files are available below the reviewer comments at the end of this letter.

If applicable, we recommend that you deposit your laboratory protocols in protocols.io to enhance the reproducibility of your results. Protocols.io assigns your protocol its own identifier (DOI) so that it can be cited independently in the future. For instructions see: https://journals.plos.org/plosone/s/submission-guidelines#loc-laboratory-protocols. Additionally, PLOS One offers an option for publishing peer-reviewed Lab Protocol articles, which describe protocols hosted on protocols.io. Read more information on sharing protocols at https://plos.org/protocols?utm_medium=editorial-email&utm_source=authorletters&utm_campaign=protocols.

As the corresponding author, your ORCID iD is verified in the submission system and will appear in the published article. PLOS supports the use of ORCID, and we encourage all coauthors to register for an ORCID iD and use it as well. Please encourage your coauthors to verify their ORCID iD within the submission system before final acceptance, as unverified ORCID iDs will not appear in the published article. *Only*  the individual author can complete the verification step; PLOS staff *cannot*  verify ORCID iDs on behalf of authors.

We look forward to receiving your revised manuscript.

Kind regards,

Emanuele Paci

Academic Editor

PLOS One

Journal Requirements:

When submitting your revision, we need you to address these additional requirements.

1. Please ensure that your manuscript meets PLOS ONE's style requirements, including those for file naming. The PLOS ONE style templates can be found at

https://journals.plos.org/plosone/s/file?id=wjVg/PLOSOne_formatting_sample_main_body.pdf and https://journals.plos.org/plosone/s/file?id=ba62/PLOSOne_formatting_sample_title_authors_affiliations.pdf

2. Your ethics statement should only appear in the Methods section of your manuscript. If your ethics statement is written in any section besides the Methods, please delete it from any other section.

3. We note that the grant information you provided in the ‘Funding Information’ and ‘Financial Disclosure’ sections do not match.

When you resubmit, please ensure that you provide the correct grant numbers for the awards you received for your study in the ‘Funding Information’ section.

4. Please amend your list of authors on the manuscript to ensure that each author is linked to an affiliation. Authors’ affiliations should reflect the institution where the work was done (if authors moved subsequently, you can also list the new affiliation stating “current affiliation:….” as necessary).

5. We are unable to open your Supporting Information file [manuscript_plos.tex]. Please kindly revise as necessary and re-upload.

6. We note that Figure S2, S6, in your submission contain copyrighted images. All PLOS content is published under the Creative Commons Attribution License (CC BY 4.0), which means that the manuscript, images, and Supporting Information files will be freely available online, and any third party is permitted to access, download, copy, distribute, and use these materials in any way, even commercially, with proper attribution. For more information, see our copyright guidelines: http://journals.plos.org/plosone/s/licenses-and-copyright.

We require you to either (1) present written permission from the copyright holder to publish these figures specifically under the CC BY 4.0 license, or (2) remove the figures from your submission:

a. You may seek permission from the original copyright holder of Figure S2, S6, to publish the content specifically under the CC BY 4.0 license.

We recommend that you contact the original copyright holder with the Content Permission Form (http://journals.plos.org/plosone/s/file?id=7c09/content-permission-form.pdf) and the following text:

“I request permission for the open-access journal PLOS ONE to publish XXX under the Creative Commons Attribution License (CCAL) CC BY 4.0 (http://creativecommons.org/licenses/by/4.0/). Please be aware that this license allows unrestricted use and distribution, even commercially, by third parties. Please reply and provide explicit written permission to publish XXX under a CC BY license and complete the attached form.”

Please upload the completed Content Permission Form or other proof of granted permissions as an "Other" file with your submission.

In the figure caption of the copyrighted figure, please include the following text: “Reprinted from [ref] under a CC BY license, with permission from [name of publisher], original copyright [original copyright year].”

b. If you are unable to obtain permission from the original copyright holder to publish these figures under the CC BY 4.0 license or if the copyright holder’s requirements are incompatible with the CC BY 4.0 license, please either i) remove the figure or ii) supply a replacement figure that complies with the CC BY 4.0 license. Please check copyright information on all replacement figures and update the figure caption with source information. If applicable, please specify in the figure caption text when a figure is similar but not identical to the original image and is therefore for illustrative purposes only.

7. If the reviewer comments include a recommendation to cite specific previously published works, please review and evaluate these publications to determine whether they are relevant and should be cited. There is no requirement to cite these works unless the editor has indicated otherwise.

[Note: HTML markup is below. Please do not edit.]

Reviewers' comments:

Reviewer's Responses to Questions

**Comments to the Author**

1. Is the manuscript technically sound, and do the data support the conclusions?

Reviewer #1: Yes

**********

2. Has the statistical analysis been performed appropriately and rigorously?

Reviewer #1: Yes

**********

3. Have the authors made all data underlying the findings in their manuscript fully available?

The PLOS Data policy

Reviewer #1: Yes

**********

4. Is the manuscript presented in an intelligible fashion and written in standard English?

Reviewer #1: Yes

**********

Reviewer #1: This manuscript presents an application of focal molography, a relatively new label-free optical biosensing technique, to measure temperature-dependent protein–ligand binding kinetics and derive apparent thermodynamic parameters. Unlike established methods such as surface plasmon resonance (SPR), which detect changes in refractive index near a sensor surface, focal molography measures coherent light scattered from molecules bound to a periodically patterned surface. This makes it intrinsically less sensitive to homogeneous refractive-index changes caused by temperature fluctuations, buffer exchange, or complex biological media. The manuscript demonstrates these advantages, particularly reduced thermal drift and measurements in serum. The combination with DNA-directed immobilization for multiplexed measurements is also technically attractive.

The work is well executed and addresses an interesting technological development. However, I believe the manuscript requires major revision before publication.

The principal weakness is the interpretation of the thermodynamic analysis. While the authors appropriately describe the extracted quantities as "apparent" thermodynamic fingerprints, the manuscript still tends to overstate their physical significance. The comparison with published SPR and ITC data clearly shows that the enthalpy–entropy decomposition depends strongly on the assay format and surface presentation. Consequently, these quantities should be presented as qualitative descriptors within the focal molography platform rather than as intrinsic thermodynamic properties of the molecular interaction.

A second concern is the limited statistical independence of the data. Although a large number of kinetic fits are reported, these arise predominantly from technical replicates rather than independent chip preparations, ligand conjugations, or protein batches. The uncertainty associated with the reported thermodynamic parameters is therefore likely underestimated.

The manuscript would also benefit from a clearer discussion of the limitations imposed by surface immobilization, DNA-directed ligand presentation, and the use of a non-dissociating fraction model for some ligands. These factors may influence both the kinetic and thermodynamic parameters and deserve more explicit consideration.

There is also a disclosure/consistency issue. The competing interest is properly declared: two authors are employees of lino Biotech AG, which commercializes focal molography, and the work was funded by the same company. But the statement that the funder had “no role” is awkward because the funder and the authors’ employer are effectively the same organization. The data availability statement also appears inconsistent: the submission form mentions a Zenodo DOI, whereas the manuscript text says data are provided as Supporting Information.

In summary, this is a technically sound and potentially valuable methodological contribution. Focal molography is not yet an established alternative to SPR or related label-free biosensing techniques, but it offers genuine advantages for measurements under changing temperature and complex sample conditions. However, the thermodynamic conclusions require more cautious interpretation, and several methodological limitations should be discussed more explicitly.

Recommendation: Major revision.

**********

what does this mean?). If published, this will include your full peer review and any attached files.

If you choose “no”, your identity will remain anonymous but your review may still be made public.

**Do you want your identity to be public for this peer review?**  For information about this choice, including consent withdrawal, please see our Privacy Policy

Reviewer #1: No

**********

[NOTE: If reviewer comments were submitted as an attachment file, they will be attached to this email and accessible via the submission site. Please log into your account, locate the manuscript record, and check for the action link "View Attachments". If this link does not appear, there are no attachment files.]

To ensure your figures meet our technical requirements, please review our figure guidelines: https://journals.plos.org/plosone/s/figures

You may also use PLOS’s free figure tool, NAAS, to help you prepare publication quality figures: https://journals.plos.org/plosone/s/figures#loc-tools-for-figure-preparation.

NAAS will assess whether your figures meet our technical requirements by comparing each figure against our figure specifications.

# Author comment: Author response to Decision Letter 1

**DOI:** 10.1371/journal.pone.0354008.r002

15 Sep 2026

Dear Dr. Paci and Reviewer,

We thank the Academic Editor and Reviewer #1 for their careful and constructive evaluation of

our manuscript, and for recognizing the work as a “technically sound and potentially valuable

methodological contribution.” We have revised the manuscript to address every point raised.

In brief, we have:

1. Reframed all extracted thermodynamic quantities as apparent, format-specific,

qualitative descriptors of the focal molography measurement—rather than intrinsic

properties of the molecular interaction—consistently across the abstract, Results, Methods,

table/figure captions, and Discussion (Reviewer #1’s principal point).

2. Expanded the limitations discussion to treat explicitly the eﬀects of surface immobi-

lization, DNA-directed ligand presentation, and the non-dissociating-fraction model on the

kinetic and thermodynamic parameters.

3. Clarified the statistical basis of the reported uncertainties, making explicit that

they capture within-assay (technical and assay) variability rather than independent chip-

to-chip, protein-preparation, or conjugate-preparation reproducibility.

4. Revised the Funding statement to reflect that the funder and the authors’ employer

are the same organization, replacing the previous blanket “no role” wording.

5. Reconciled the Data Availability statement with the submission system, designating

the Zenodo repository as the single source of all underlying data.

6. Addressed all editorial requirements: the ethics statement now appears only in the

Methods, every author is linked to an aﬃliation (including a current-aﬃliation entry for J.

Oehninger), and the copyright status of Figures S2 and S6 is clarified as original, author-

generated content.

Below we address each comment in detail. Reviewer and editor comments are shown in blue; our

responses are in black. Changes are marked in the “Revised Manuscript with Track Changes”

file; unmarked text is quoted here for convenience.

Academic Editor — Journal Requirements

1. Please ensure that your manuscript meets PLOS ONE’s style requirements,

including those for file naming.

The manuscript has been prepared using the PLOS ONE templates, and all files are named

according to the submission guidelines (Manuscript; Revised Manuscript with Track Changes;

Response to Reviewers; Supporting Information).

1

2. Your ethics statement should only appear in the Methods section of your

manuscript.

The ethics statement previously appeared as a stand-alone section in the back matter. We have

moved it into the Materials and methods section (now a subsection, “Ethics statement,” at the

end of Methods) and removed it from all other locations; it therefore now appears only within

the Methods.

3. We note that the grant information you provided in the ‘Funding Information’

and ‘Financial Disclosure’ sections do not match.

This study was funded solely by lino Biotech AG as internal (company) funding; there is no

external grant and therefore no associated grant number. We have ensured that the ‘Funding

Information’ and ‘Financial Disclosure’ fields in the submission system state this consistently,

and we have reworded the manuscript Funding section accordingly (see Reviewer #1, point 4).

4. Please amend your list of authors on the manuscript to ensure that each author

is linked to an aﬃliation.

All three authors are now linked to an aﬃliation. The work was performed at the R & D

department of lino Biotech AG (aﬃliation 1), the aﬃliation of all authors. As J. Oehninger has

since moved institutions, his present aﬃliation is additionally indicated with a current-aﬃliation

marker: “Current aﬃliation: Eawag, Swiss Federal Institute of Aquatic Science and Technology,

D¨ubendorf, Switzerland.” The aﬃliation where the work was performed (lino Biotech AG) is

retained for all authors.

5. We are unable to open your Supporting Information file [manuscript plos.tex].

The file in question is the LaTeX source of the main manuscript (manuscript plos.tex), which

had been uploaded as the LaTeX Source item without the auxiliary files (.bib, .bst, .bbl)

needed to compile it. As the manuscript is reviewed from the compiled PDF at this stage, we

have removed the incomplete LaTeX source item to resolve the issue; we will provide the com-

plete, compilable LaTeX source upon acceptance, in line with PLOS guidelines. The Supporting

Information file itself (S1 File.pdf) was unaﬀected and opens normally.

6. We note that Figure S2, S6 in your submission contain copyrighted images.

Figures S2 and S6 are original figures prepared by the authors specifically for this manuscript.

They consist of photographs and schematics of the instrumentation used in this study, taken and

produced by the authors, and have not been published elsewhere; they contain no third-party

or previously published copyrighted material. The copyright holder, lino Biotech AG, consents

to their publication under the Creative Commons Attribution License (CC BY 4.0).

We note for completeness that the images depict commercial instrumentation bearing the

MACS® trademark (a registered trademark of Miltenyi Biotec) and incidental third-party labo-

ratory hardware (e.g. a Thorlabs temperature controller). These appear only as a factual record

of the experimental setup; no copyrighted image belonging to those parties is reproduced, and

the trademark is used descriptively.

7. If the reviewer comments include a recommendation to cite specific previously

published works, please review and evaluate these publications. . .

Reviewer #1 did not request the citation of any specific works. No citations were added on this

basis.

2

Reviewer #1

This manuscript presents an application of focal molography. . . to measure temperature-

dependent protein–ligand binding kinetics and derive apparent thermodynamic pa-

rameters. . . . The work is well executed and addresses an interesting technological

development. However, I believe the manuscript requires major revision before

publication.

We thank the reviewer for this positive and constructive assessment, and for recognizing the

advantages of focal molography for measurements under changing temperature and in complex

matrices. We have addressed each of the concerns raised, as detailed below.

Major Points

1. The principal weakness is the interpretation of the thermodynamic analysis.

While the authors appropriately describe the extracted quantities as “apparent”

thermodynamic fingerprints, the manuscript still tends to overstate their physical

significance. The comparison with published SPR and ITC data clearly shows

that the enthalpy–entropy decomposition depends strongly on the assay format

and surface presentation. Consequently, these quantities should be presented as

qualitative descriptors within the focal molography platform rather than as intrinsic

thermodynamic properties of the molecular interaction.

We fully agree and have revised the manuscript so that the extracted quantities are consistently

presented as apparent, format-specific, qualitative descriptors of the focal molography

measurement, not as intrinsic thermodynamic properties of the cAMP–PKA-R interaction:

• Results narrative. We removed the remaining categorical, physical-property phrasings.

For example, “all five show exothermic binding. . . but the balance between enthalpic and

entropic contributions diﬀers” now reads “all five show an apparently exothermic binding

signature (apparent ∆H◦. . . ), but the apparent balance between enthalpic and entropic

contributions diﬀers between ligands”; “2-AHA-cAMP is more strongly enthalpy-driven”

now reads “2-AHA-cAMP shows a more strongly enthalpy-weighted apparent profile”;

and the summary sentence now refers to a “distinct but internally consistent apparent

thermodynamic profile.”

• Methods. The van ’t Hoﬀ parameters are now introduced as “Apparent standard-state

thermodynamic parameters,” with an explicit statement that the “◦” superscript denotes

only the 1 M concentration reference and that these are “apparent, format-specific pa-

rameters of the surface-based assay rather than solution-phase constants.”

• Captions. The summary tables are now titled “apparent equilibrium thermodynamic

parameters” and “apparent activation parameters.”

• Introduction. References to the output now read “distinct, apparent thermodynamic

fingerprints” / “apparent enthalpic and entropic fingerprints.”

• Discussion. We retained and reinforced the statements that the parameters “are appar-

ent, format-specific quantities, most powerful for ranking and contrasting related ligands

rather than as substitutes for calorimetric reference values,” that they are “apparent prop-

erties of the DNA-tethered, surface-confined presentation rather than of the free-solution

interaction,” and that “the absolute enthalpy–entropy split is the least transferable part

of the comparison.” The cross-method (SPR/ITC) comparison is explicitly framed as

“external context rather than. . . a test of thermodynamic equivalence.”

3

Together these changes ensure the thermodynamic quantities are consistently framed as quali-

tative, platform-specific descriptors for comparative ranking, as the reviewer recommends.

2. A second concern is the limited statistical independence of the data. Although

a large number of kinetic fits are reported, these arise predominantly from tech-

nical replicates rather than independent chip preparations, ligand conjugations,

or protein batches. The uncertainty associated with the reported thermodynamic

parameters is therefore likely underestimated.

We agree, and we note that the manuscript already makes this limitation explicit; we have en-

sured it is unambiguous. In the Methods (“Thermodynamic parameter uncertainties”), we state

that the bootstrap standard deviations “capture within-assay variability among retained molo-

grams, cycles, and experimental sessions, but not independent chip-to-chip, protein-preparation,

or conjugate-preparation variability.” In the Discussion (“Interpretation boundaries and mea-

surement limitations”), we state that the measurements underlying each analysis (N = 64 per

compound) “are technical and assay replicates. . . rather than fully independent biochemical

replicates, so the bootstrap uncertainties mainly capture within-assay measurement and surface

variability, not between-preparation reproducibility.” We therefore explicitly caution that the

reported uncertainties reflect within-assay precision, and we treat the parameters as compara-

tive rather than absolute throughout.

3. The manuscript would also benefit from a clearer discussion of the limitations

imposed by surface immobilization, DNA-directed ligand presentation, and the use

of a non-dissociating fraction model for some ligands. These factors may influ-

ence both the kinetic and thermodynamic parameters and deserve more explicit

consideration.

We have substantially expanded the limitations discussion to treat each of these three factors

explicitly:

• Surface immobilization (new). Immobilization itself—independently of the coupling

chemistry—removes the ligand’s translational and rotational freedom relative to the ITC

solution reference, confines the interaction to a two-dimensional, locally crowded layer

that can promote rebinding and thereby slow the apparent dissociation, and embeds it in

the hydrated PEG-brush environment rather than in bulk solvent; each of these eﬀects can

shift the measured rate constants and, through them, the enthalpic and (especially) en-

tropic terms, and is a generic reason why surface-based and solution-based decompositions

need not agree.

• DNA-directed ligand presentation (expanded). The DNA tether sets the ligand’s

distance from the surface and carries a dense negative charge that modifies the local elec-

trostatics, hydration, and accessible orientational ensemble, while spot-to-spot variation in

hybridization density adds a further source of heterogeneity—so the reported parameters

are apparent properties of the DNA-tethered, surface-confined presentation.

• Non-dissociating fraction model (expanded). The Langmuir 1:1 NonDissRelative

model represents the sensorgram as a dissociating Langmuir population superposed on an

apparently static, non-releasing fraction; such a fraction is itself a signature of surface het-

erogeneity (e.g. a slow-oﬀ subpopulation, avidity/rebinding-trapped complexes, or partial

irreversible capture); the fitted parameters describe the dissociating subpopulation only;

and a temperature-dependent non-dissociating fraction could bias the apparent van ’t Hoﬀ

and Eyring temperature dependence for 8-ADOA-cAMP and 2-AHA-cAMP specifically,

making their parameters the most model-dependent in the panel.

4

4. There is also a disclosure/consistency issue. The competing interest is properly

declared. . . But the statement that the funder had “no role” is awkward because

the funder and the authors’ employer are eﬀectively the same organization. The

data availability statement also appears inconsistent: the submission form mentions

a Zenodo DOI, whereas the manuscript text says data are provided as Supporting

Information.

We agree on both counts and have corrected them.

• Funding statement. We removed the blanket “no role” wording. The Funding section

now acknowledges that the funder and the authors’ employer are the same organization,

and states that “the funder was necessarily involved in the study through the authors

themselves—in study design, data collection and analysis, the decision to publish, and

preparation of the manuscript,” while clarifying that “no external sponsor or third party

had any role in the study.” It cross-references the Author contributions and Conflict of

Interest sections.

• Data availability. We reconciled the statement with the submission form. The manuscript

now states that all data underlying the findings—raw measurement exports, processed

data tables, fitted-trace summaries, and the analysis and figure-generation scripts—are

openly available in the Zenodo repository (DOI: 10.5281/zenodo.21103143), “consistent

with the Data Availability entry in the submission system,” and that the Supporting

Information files “provide supplementary methods, figures, and tables only and do not

contain additional underlying data.”

Summary of Changes

1. Apparent-parameter framing: thermodynamic and activation quantities are consis-

tently presented as apparent, format-specific, qualitative descriptors throughout the manuscript.

2. Expanded limitations: explicit treatment of surface immobilization, DNA-directed lig-

and presentation, and the non-dissociating-fraction model.

3. Statistical basis clarified: reported uncertainties are stated to capture within-assay, not

between-preparation, variability.

4. Funding statement revised to reflect that the funder and the authors’ employer are the

same organization.

5. Data availability reconciled with the submission system (Zenodo as the single source of

underlying data).

6. Editorial requirements: ethics statement moved into Methods; all authors linked to

aﬃliations (with a current-aﬃliation entry for J. Oehninger); Figures S2/S6 copyright

status clarified as original author-generated content.

We believe these revisions fully address the comments raised and have improved the manuscript,

particularly by ensuring the thermodynamic quantities are consistently and appropriately framed

as apparent, comparative descriptors. We thank the reviewer and editor again for their time

and constructive input.

Sincerely,

John Oehninger, Simona Notova, and Andreas Frutiger

Attachment Submitted filename: Response_to_Reviewers.pdf

# Editor report: Decision Letter 1

**DOI:** 10.1371/journal.pone.0354008.r003

**Authors:** Emanuele Paci

17 Sep 2026

High-throughput thermodynamic fingerprinting of protein–ligand interactions by DNA-directed focal molography

PONE-D-26-32924R1

Dear Dr. Frutiger,

We’re pleased to inform you that your manuscript has been judged scientifically suitable for publication and will be formally accepted for publication once it meets all outstanding technical requirements.

Within one week, you’ll receive an e-mail detailing the required amendments. When these have been addressed, you’ll receive a formal acceptance letter and your manuscript will be scheduled for publication.

An invoice will be generated when your article is formally accepted. Please note, if your institution has a publishing partnership with PLOS and your article meets the relevant criteria, all or part of your publication costs will be covered. Please make sure your user information is up-to-date by logging into Editorial Manager at Editorial Manager® and clicking the ‘Update My Information' link at the top of the page. For questions related to billing, please contact billing support.

If your institution or institutions have a press office, please notify them about your upcoming paper to help maximize its impact. If they’ll be preparing press materials, please inform our press team as soon as possible -- no later than 48 hours after receiving the formal acceptance. Your manuscript will remain under strict press embargo until 2 pm Eastern Time on the date of publication. For more information, please contact onepress@plos.org.

Kind regards,

Emanuele Paci

Academic Editor

PLOS One

Additional Editor Comments (optional):

Reviewers' comments:

# Editor report: Acceptance letter

**DOI:** 10.1371/journal.pone.0354008.r004

**Authors:** Emanuele Paci

PONE-D-26-32924R1

PLOS One

Dear Dr. Frutiger,

I'm pleased to inform you that your manuscript has been deemed suitable for publication in PLOS One. Congratulations! Your manuscript is now being handed over to our production team.

At this stage, our production department will prepare your paper for publication. This includes ensuring the following:

* All references, tables, and figures are properly cited

* All relevant supporting information is included in the manuscript submission,

* There are no issues that prevent the paper from being properly typeset

You will receive further instructions from the production team, including instructions on how to review your proof when it is ready. Please keep in mind that we are working through a large volume of accepted articles, so please give us a few days to review your paper and let you know the next and final steps.

Lastly, if your institution or institutions have a press office, please let them know about your upcoming paper now to help maximize its impact. If they'll be preparing press materials, please inform our press team within the next 48 hours. Your manuscript will remain under strict press embargo until 2 pm Eastern Time on the date of publication. For more information, please contact onepress@plos.org.

You will receive an invoice from PLOS for your publication fee after your manuscript has reached the completed accept phase. If you receive an email requesting payment before acceptance or for any other service, this may be a phishing scheme. Learn how to identify phishing emails and protect your accounts at https://explore.plos.org/phishing.

If we can help with anything else, please email us at customercare@plos.org.

Thank you for submitting your work to PLOS One and supporting open access.

Kind regards,

PLOS One Editorial Office Staff

on behalf of

Dr. Emanuele Paci

Academic Editor

PLOS One
